# New insights into the mechanism of nickel superoxide degradation from studies of model peptides

**DOI:** 10.1038/s41598-017-17446-3

**Published:** 2017-12-08

**Authors:** Daniel Tietze, Jana Sartorius, Banabithi Koley Seth, Kevin Herr, Pascal Heimer, Diana Imhof, Doreen Mollenhauer, Gerd Buntkowsky

**Affiliations:** 1 Eduard-Zintl Institute for Physical and Inorganic Chemistry, Darmstadt University of Technology, Alarich-Weiss-Str. 8, 64287 Darmstadt, Germany; 2 Pharmaceutical Biochemistry and Bioanalytics, Pharmaceutical Institute, University of Bonn, An der Immenburg 4, D-53119 Bonn, Germany; 3 Institute of Physical Chemistry, Justus Liebig University Giessen, Heinrich-Buff-Ring 17, D-35392 Giessen, Germany

## Abstract

A series of small, catalytically active metallopeptides, which were derived from the nickel superoxide dismutase (NiSOD) active site were employed to study the mechanism of superoxide degradation especially focusing on the role of the axial imidazole ligand. In the literature, there are contradicting propositions about the catalytic importance of the N-terminal histidine. Therefore, we studied the stability and activity of a set of eight NiSOD model peptides, which represent the major model systems discussed in the literature to date, yet differing in their length and their Ni-coordination. UV-Vis-coupled stopped-flow kinetic measurements and mass spectrometry analysis unveiled their high oxidation sensitivity in the presence of oxygen and superoxide resulting into a much faster Ni(II)-peptide degradation for the amine/amide Ni(II) coordination than for the catalytically inactive bis-amidate Ni(II) coordination. With respect to these results we determined the catalytic activities for all NiSOD mimics studied herein, which turned out to be in almost the same range of about 2 × 10^6^ M^−1^ s^−1^. From these experiments, we concluded that the amine/amide Ni(II) coordination is clearly the key factor for catalytic activity. Finally, we were able to clarify the role of the N-terminal histidine and to resolve the contradictory literature propositions, reported in previous studies.

## Introduction

Superoxide is constantly formed in aerobic organisms as a byproduct of cellular respiration and represents a highly reactive oxygen species which can cause severe damage to the cell^1^. Superoxide dismutases (SOD) are the key factors in nature’s defense strategy against superoxide. Although superoxide is cytotoxic and a major reason of aging, it is involved in diverse cell signaling processes^1^. So far, its regulatory character relies on the ability to interact with reactive cysteine residues and influences their redox state in order to regulate enzymatic activities resulting in a so-called redox-dependent signaling^2^. Even though SODs are known for many decades, a novel class of SODs was discovered only 20 years ago, which is mainly present in distinct *Streptomyces* strains and cyanobacteria^3–6^. This novel class of SODs carries Ni as a cofactor and catalyzes superoxide degradation forming H_2_O_2_ and O_2_^4,7^. Thereby, superoxide dismutases generally operate under diffusion limiting conditions with rate constants for the superoxide degradation of about 2 × 10^9^ M^−1^ s^−1^ ^8^. As a redox-active enzyme NiSOD exhibits a quite unusual active site, which is formed by a square planar Ni^2+^ coordination environment in which the N-terminal residues His1, Cys2 and Cys6 serve as ligands for the central Ni-ion providing two nitrogen and two sulfur donor atoms^9,10^. In 2004, Barondeau *et al*. postulated a catalytic mechanism of superoxide degradation by NiSOD which is still under intense investigation^11,12^. However, the study of small peptide models derived from the native enzyme led to a substantial progress in understanding the mechanism of superoxide degradation (e.g. see ref.^13^ and work cited therein). Concerning the source of the proton for the hydrogen peroxide formation, recent studies on NiSOD model peptides indicate that aside from the phenolic proton of Tyr9 or the amide proton of Cys6 an active site bound water molecule was suggested to be the proton source^14^.

Furthermore, Shearer *et al*. proposed that the mechanism of superoxide degradation induced by these small model peptides may be substantially different from the enzyme^11,12^.

Earlier studies on the crystal structure of the native enzyme revealed a hydrogen bond network which is believed to fine-tune the electronic properties of the imidazole ring of His1 and keep it in place to avoid large structural rearrangements at the active site when coordinating/un-coordinating during the catalytic cycle of superoxide degradation^9^.

The putative influence of this hydrogen bond network was investigated through a series of small model peptides with N_ε_-substituted histidines yielding a significantly increased catalytic activity when the axial imidazole was less Lewis basic^15^. Albeit these results clearly claim an important role for the N-terminal His residue, Schmidt and co-workers observed almost no changes of the catalytic activity, when this residue was mutated to Ala for their Nim^9^SOD model peptides^16^. In contrast, Shearer *et al*. observed a significant reduction of the catalytic activity for the same mutation within a slightly shorter peptide (Nim^7^SOD H1A) in an earlier study. However, the catalytic activity of several *S. seoulensis* NiSOD H1X mutations such as H1A and H1Q determined in cell extracts was below the detection limit (~5%) of the standard cytochrome C assay^9^. More precise activity measurements through direct observation of pulse radiolytically generated superoxide revealed a residual activity of ~8 × 10^6^ M^−1^ s^−1^ (pH 8.0) for the NiSOD H1Q^8^ mutant and 47 × 10^6^ M^−1^ s^−1^ (pH 7.5) for the NiSOD H1A^17^ mutant. While the electronic environment of the central Ni(II) ion influences the activity significantly, no major impact on the activity was found for model peptides which are composed of the first 7, 9 or 12 N-terminal residues (in the following abbreviated as Nim^7^SOD, Nim^9^SOD, Nim^12^SOD) of the wild type enzyme thus exceeding the Ni-binding hook^15,16,18^.

**Figure 1:**
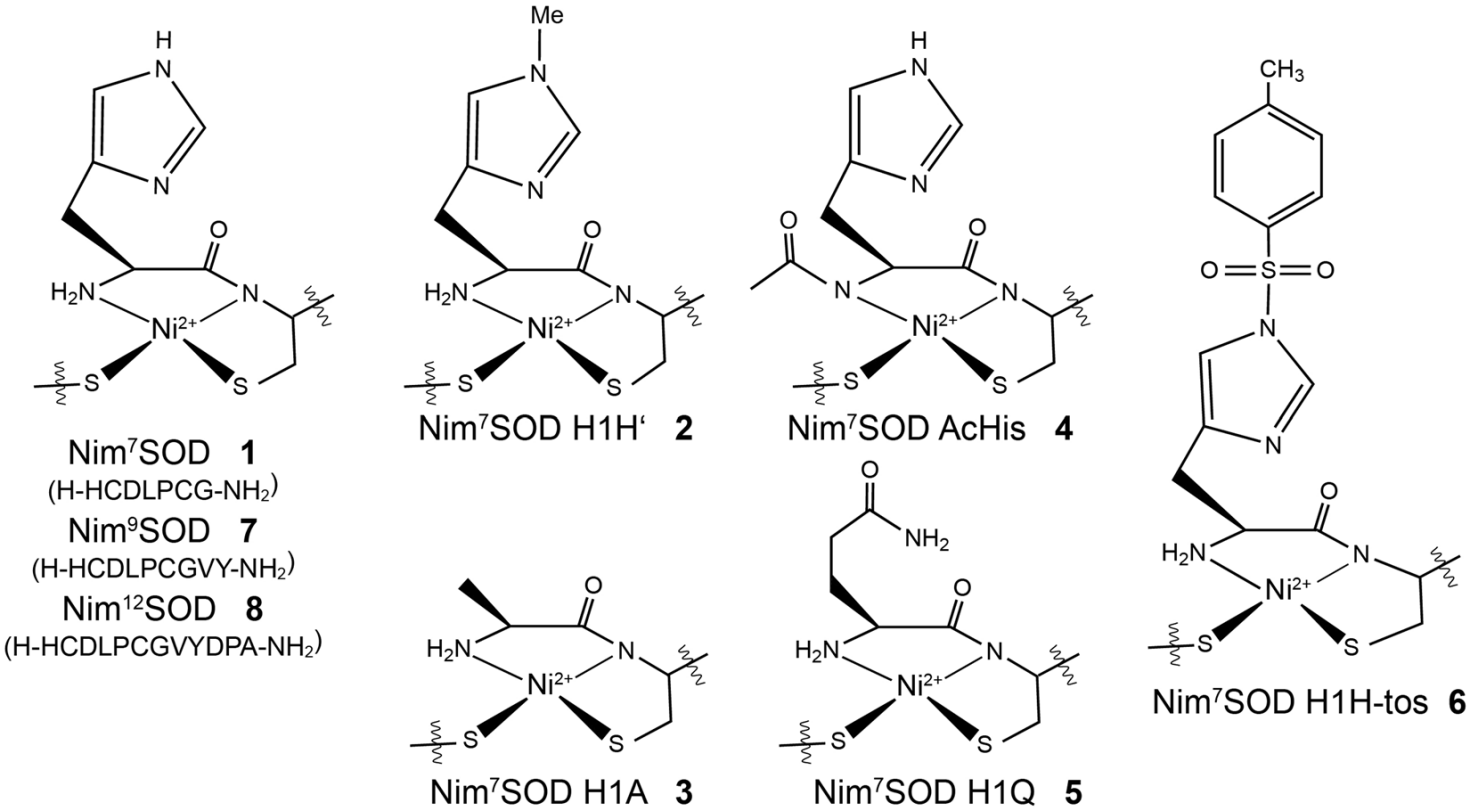
Overview of the peptide-based NiSOD mimics studied in this work.

As already mentioned NiSOD is a redox-active enzyme and reveals a rather unusual active site. Hence, the question arises how the enzyme protects the coordinating cysteine residues from being oxidized during superoxide degradation. How this is achieved by the enzyme is still not clear. While non-peptidic NiSOD model systems are almost exclusively catalytically inactive and prone to ligand-based redox processes (see ref.^19^ and work cited therein), peptide-based NiSOD maquettes were assumed to be stable against oxygen^20^ and predicted to be unstable against H_2_O_2_ on the basis of theoretical calculations^21^. In a more recent study a bis-amidate NiSOD model peptide was found to show rapid ligand-based sulfur oxidation which was thought to cause its inactivity while no such oxidation was observed for the native-like amine/amide metallopeptide^20,22,23^. This is rather surprising since the same native-like amine/amide NiSOD mimic was observed to degrade rapidly on air in an early study from the same author^24^. From these results and, taking into account the aforementioned theoretical calculations the authors concluded, that the asymmetric amine/amide ligand sphere of the enzyme and the NiSOD metallopeptides must be the key factor in protecting the cysteinate sulfur atoms from oxidation^20^. However, except for these two studies no systematic analysis of the stability of the NiSOD mimic was performed thus far^13,20^. Beside the fact, that many hypothesis are still based on activity data obtained from different methods and under non-comparable conditions, air sensitivity of the Ni(II) model peptides was not yet considered for the activity measurements. Therefore, we determined the stability and activity of a set of eight NiSOD model peptides, which represent the major model systems discussed in the literature to date, yet differing in their length and their Ni-coordination (Fig. 1, peptides **1**, **7**, **8** and **4**). From these experiments, we were finally able to unveil the role of the N-terminal histidine (Fig. 1, peptides **2**, **3**, **5** and **6**) clarifying contradictory literature reports^15–17^.

In order to study the stability of these NiSOD mimics against KO_2_, peptide oxidation was observed photometrically using a stopped-flow system and electrospray ionization (ESI) mass spectrometry. Our experiments indicate that all NiSOD mimics are highly unstable showing sulfur oxidation through the formation of disulfide-bridges between monomeric peptide units and sulfur oxygenation. Rate constants for peptide oxidation were all in the same range except for the bis-amidate Ni-coordinating peptide which was about an order of magnitude more stable contradicting recent literature observations. In order to correlate peptide stability and catalytic activity, we re-investigated the catalytic activity of these NiSOD model peptides, confirming earlier literature findings that the bis-amidate Ni-peptide was catalytically inactive. Moreover, and in contrast to literature reports, we found that catalytic activities for all other NiSOD mimics studied herein were in almost the same range of about 2 × 10^6^ M^−1^ s^−1^ allowing to conclude that the N-terminal His is not required for catalytic activity, solving a long-standing dispute in the literature. Consequently, the mechanism of superoxide degradation by the NiSOD model peptides must be different from the enzyme. Furthermore, our experiments unveil that the ability to degrade superoxide by the model peptides is coupled to an increased sensitivity for oxidation, which results from the asymmetric amine/amide Ni(II) coordination.

## Results

### Synthesis

After the synthesis of the linear peptides (Fig. 1, Supporting Information Figs S1–S8) 1 equiv. NiCl_2_ (aq.) was added to a freshly prepared solution (150 mM phosphate buffer, pH 8) of each peptide to yield the Ni-peptide complex whose formation was ensured via UV-Vis spectroscopy^25^. The resulting UV-Vis spectra of the NiSOD metallopeptides reproduced earlier literature findings (*see* Supporting Information Fig. S9) and confirmed the almost identical electronic structure for **1**–**3** and **5**–**8** ^14,15,24–26^. However, a rapid loss of the reddish color of Ni(II)-peptide **7** was observed, which turned into a slight yellow or almost colorless solution after exposure to air for 24 h when peptide **7** was prepared NiSOD under aerobic conditions. HPLC and high-resolution mass spectrometry analysis of the matured Ni(II)-peptide solution revealed a complete conversion of the peptide indicating the formation of disulfide-bridged di-, tri- and tetramers (*see* Supporting Information Fig. S10). These, observations obliged us to use the Ni(II)-peptides under fully anaerobic conditions. Moreover, the oxidative environment created by the products of superoxide degradation under which the catalytic activity of these peptides is typically determined, enforced us to question the stability of these model peptides. Additionally, we were interested if an increased stability would also result in an increased activity. Thus, we decided to study the oxidation process of NiSOD model peptides under the conditions typically employed to assess their catalytic activity.

### Ni(II)-peptide degradation investigated by stopped-flow and LC-MS techniques

#### Stopped-flow experiments

**Figure 2:**
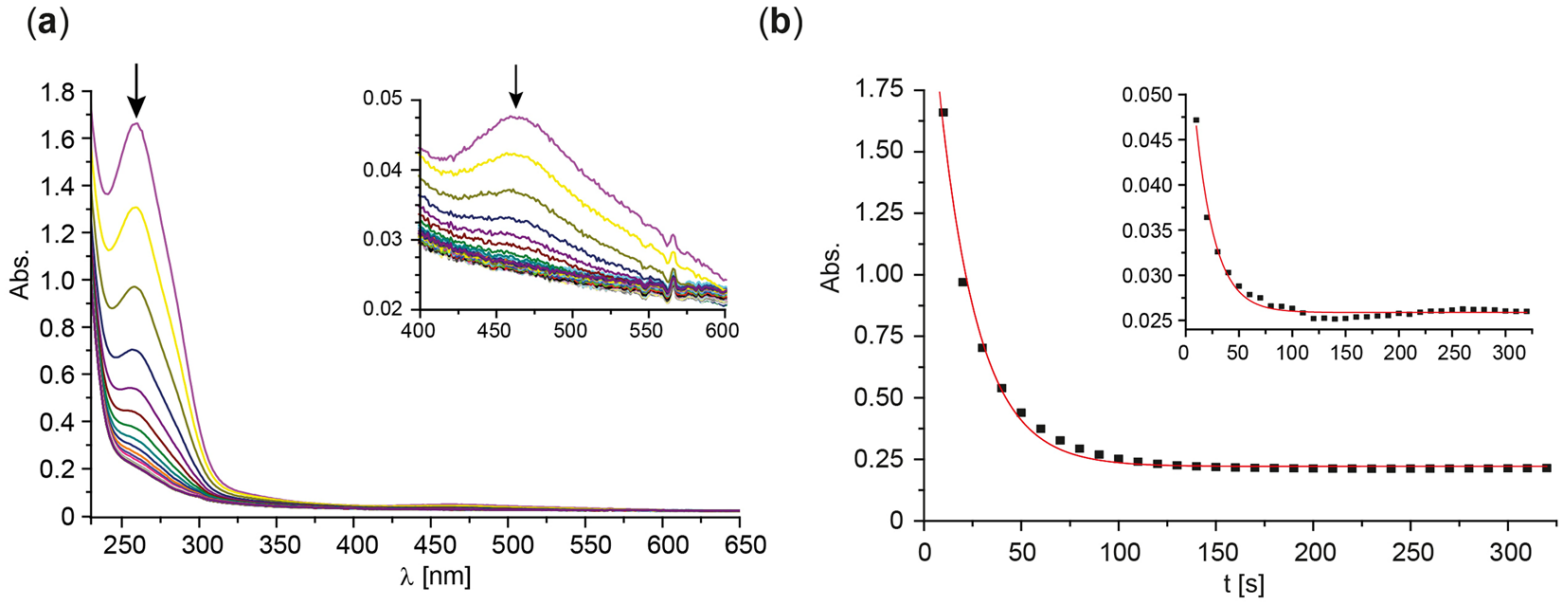
KO_2_ (5 mM) induced decomposition of Nim^7^SOD (1) (0.316 mM, 150 mM phosphate buffer, pH 8.0, 25 °C) (**a**) monitored via UV-Vis/stopped flow experiments (for clarity, spectra are shown at a 10 s interval for 10 to 350 s). (**b**) time traces (black squares) of Ni(II)-peptide decomposition extracted at 258 nm and 460 nm (inset). The data were fitted via a 1^st^ order decay (red line).

According to literature reports, decomposition of the peptide/Ni(II)-complex through oxidative damage of the peptide can be observed following the changes of the characteristic sulfur-to-Ni(II) ligand field transition around 460 nm which indicates the loss of the central Ni(II) ion^16,24^. According to the procedure reported therein for assessing the catalytic activity of the NiSOD mimics decomposition was determined by mixing a buffered solution of the Ni(II)-peptide (~0.16 mM or 0.04 mM, phosphate buffer 150 mM, pH 8.0) with a superoxide solution (50 mM, DMSO/18-crown-6) in a 10:1 ratio utilizing a stopped-flow system recording the UV-Vis spectrum of the mixture over a time course of about 20 min (Fig. 2a, Supporting Information Figs S11–S17).

As shown for Nim^7^SOD (**1**) the characteristic sulfur-to-Ni(II) ligand field transition at 460 nm^25–27^ completely disappeared within the first 100 s (Fig. 2). Additionally, the strong spectral feature at 260 nm (ε ≈ 10000 to 13000 M^−1^ cm^−1^) which is not present in the metal-free peptide, also disappeared on a similar time scale indicating that this transition is also characteristic for Ni(II)-binding. An analogous electronic feature at 260 nm with comparable extinction coefficients was also present in Ni(II)-dithiothreitol complexes and attributed to Ni(II)-binding to sulfur-based ligands which further supports the Ni(II)-binding character of the 260 nm transition^28^.

Consequently, the decomposition of the Ni(II)-peptides can be monitored at least at two spectral regions, namely around 260–280 nm and 460–500 nm, as exemplarily shown for **1** (Nim^7^SOD, Fig. 2, Table 1). To extract Ni(II)-peptide half-life a first order degradation process was assumed with respect to the excess of oxygen and hydrogen peroxide present in the buffer solution (approx. 2.5 mM). A first order Ni(II)-peptide degradation process is further supported by the very similar t_1/2_ times calculated for three different peptide concentrations (Table 1, Supporting Information Fig. S18).

**Table 1:** Half-life of KO_2_ treated Nim^7^SOD obtained at different peptide concentrations.

conc. Nim^7^SOD [mM]	detection wavelength [nm]	half-life [s]
0.316	258	14 ± 0.3
0.316	460	13 ± 0.6
0.158	262	17 ± 0.2
0.158	460	15 ± 0.3
0.04	262	21 ± 0.2
	mean	16 ± 3

However, the much higher extinction coefficient at 260 nm in comparison to the 450 nm transition (~300–500 M^−1^ cm^−1^) resulted in a much better signal to noise ratio and was therefore used to calculate the half-life of the Ni(II)-peptides (Table 2) for **2** to **8** (Supporting Information Figs S11–S17).

**Table 2:** Catalytic SOD activity and stability of NiSOD model peptides obtained from stopped-flow experiments in this work and from ref.^15^ (indicated with *).

peptide		stability	activity
		*t*_*1/*2_ [s]	*k*_*cat*_ × 10^6^ [M^−1^ s^−1^]
Nim^7^SOD	**1**	^#^16 ± 3	1.7 ± 0.3
Nim^7^SOD H1H′	**2**	18 ± 0.2	2.8 ± 0.4
Nim^7^SOD H1A	**3**	45 ± 0.5	1.2 ± 0.3
Nim^7^SOD AcHis	**4**	297 ± 0.6	n.d.
Nim^7^SOD H1Q	**5**	25 ± 0.1	1.9 ± 0.3
Nim^7^SOD H1H-tos	**6**	16 ± 0.2	2.2 ± 0.4
Nim^9^SOD	**7**	18 ± 0.7	3.5 ± 0.6
Nim^12^SOD	**8**	13 ± 0.3	2.5 ± 0.4
CuZnSOD			910 ± 30
*NiSOD* ^*M*1^ *			40 ± 30
*NiSOD* ^*M1*^ -Im-Me*			6 ± 1
*NiSOD* ^*M1*^ -Im-Tos*			600 ± 200

^#^Mean value derived from three different concentrations of 1.

As outlined in Table 2, half-life of the Ni(II)-peptides were in the same range, varying between 13 s (for **8**) and 45 s (for **3**) except for **4** (Nim^7^SOD AcHis), whose half life time was almost an order of magnitude longer (~ 300 s). Evidently, experiments indicate that **4**, which coordinates Ni(II) via the symmetric bis-amide motif, is about an order of magnitude more stable against oxidation than the amine/amide Ni(II)-peptides.

In order to analyze the products of the Ni(II)-peptide degradation and to confirm ligand based oxidation, which leads to the loss of the central Ni(II)-ion as observed during the stopped flow experiments we performed a detailed LC-coupled ESI-MS analysis.

#### LC coupled mass spectrometry

**Figure 3:**
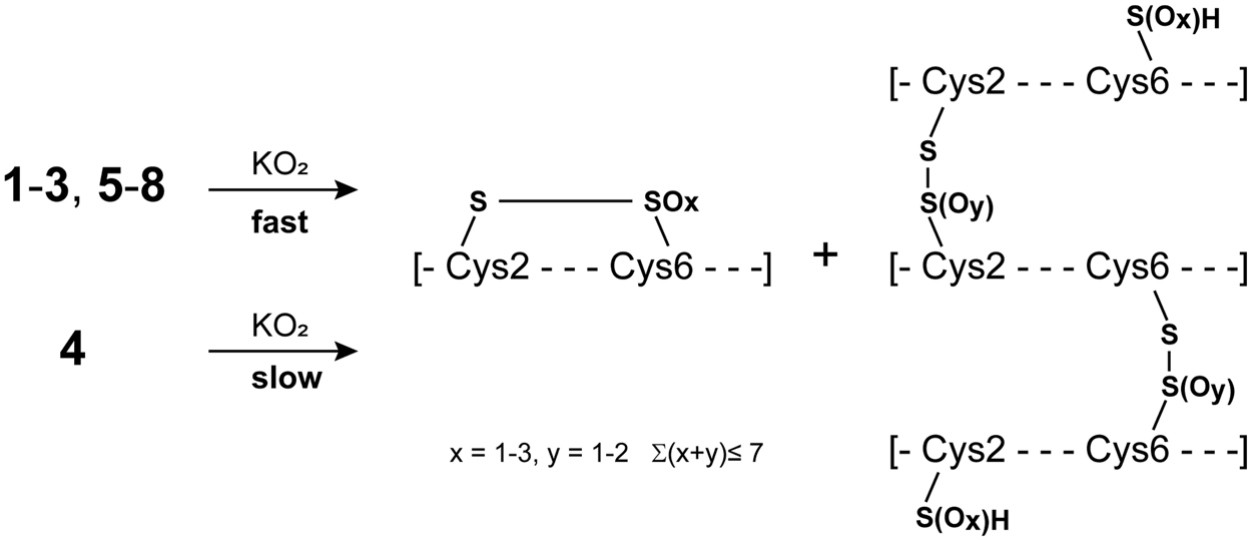
Schematic representation of the reaction of KO_2_ with the NiSOD model peptides and the oxidation products, which were identified by LC/MS analysis.

In analogy to the stopped flow stability experiments solid KO_2_ was added to a buffered Ni(II)-peptide solution. After addition of superoxide the slight-purple Ni(II)-peptide solution turned light-yellow or colorless almost immediately, indicating the destruction of the Ni(II)-peptide complex. After the resulting peptide solution was allowed to settle for 10 to 15 min, it was analyzed via LC/MS (see Supporting Information Figs S19–S26). High resolution ESI-MS analysis of the most intense peaks in the chromatogram after KO_2_ treatment of the Ni(II)-peptides unveiled unspecific oxidation of the peptides and the formation of similar degradation products (Supporting Information Figs S19–S26). Most of the degradation products were identified by comparing the isotope pattern of the experimental mass spectra with the simulated isotope pattern for the proposed species (Supporting Information Figs S19–S26) unveiling disulfide-bridged di-, tri- and tetramers (Fig. 3). Furthermore, we found, that these units can exhibit different degrees of oxygenation most likely at the sulfur atoms potentially forming sulfenates (RSO(H)), sulfinates (RSO_2_(H)), sulfonates (RSO_3_(H)), cystine-S-monoxide (RS-SO) or cystine-S-dioxide^29^. For example, tetramers were identified which were connected via three disulfide bonds and were highly oxygenated carrying seven additional oxygen atoms revealing a high degree of sulfur-based ligand oxidation. For comparison, the linear, Nickel-free peptide **1** was treated with KO_2_ and analyzed via LC/MS (Supporting Information Fig. S27). Interestingly, only disulfide-bridged monomers, dimers and trimers were observed.

#### Radical scavenger experiments

In order to unveil any radical-based Fenton-like mechanism of Ni(II)-peptide degradation, peptide oxidation by KO_2_ in the presence of the radical scavenger ascorbic acid was analyzed for **1**, **4** and **7** followed by a detailed LC/MS analysis (see Supporting Information Figs S28–S30). For **1** and **7** similar degradation products were observed as for the experiments without ascorbic acid, resulting again in disulfides and oxygenated sulfur (Supporting Information Figs S28, S30). For both peptides the relative amount of sulfur oxygenated products compared to the amount of disulfides was much smaller in the presence of ascorbic acid. Interestingly, no sulfur oxygenation was observed for **4** (Supporting Information Fig. S29).

### SOD activity of the Ni(II)-peptides investigated by stopped-flow experiments

After analyzing the oxidation process of the NiSOD model peptides, the SOD activity of these peptides was determined in strict accordance with the literature procedure^15,30,31^ and the results of the stopped-flow-based peptide-stability experiments. As described by Bolann *et al*.^31^ the superoxide decay (see eq. 1 in the method section) practically proceeds via two parallel processes: the second order self-disproportionation (*k*_2_) and the first order catalytic superoxide degradation (*k*_*obs*_, eq. 2). The rate constants for both processes can be derived from the fit of the experimental superoxide decay according to eq. 3. The rate constant *k*_*cat*_ can be derived from the linear plot of *k*_*obs*_ versus the peptide concentration (eq. 4). The superoxide decay was typically recorded over a time course of 4 s at 250 nm. Thereby, after a strong decrease of the absorption during the first 100 ms a continuously slow decreasing baseline was observed (see Supporting Information Fig. S31) which prevents a reliable fit of the superoxide decay. With respect to the strong electronic absorption of the Ni(II)-peptides around 260 nm and the peptides short half-life we concluded that the continuous decrease in the background absorption must originate from the peptide degradation process and should therefore be neglected for the analysis. Consequently, only the first 500 ms were considered for kinetic analysis (Supporting Information Fig. S31). Furthermore, literature reports indicated that this time should be sufficient to observe the complete superoxide decay under our experimental conditions^30,31^. The values of the second order self-disproportionation rates are in the range of 1.0 × 10^4^ to 4 × 10^4^ M^−1^ s^−1^, corroborating previous results from literature^15,31^. Although the peptides were highly unstable, the catalytic activities could be obtained for all of them (Table 2). Except for peptide **4** all other NiSOD mimics were catalytically active and the correlation between k_obs_ and the peptide concentration b (eq. 2) was fully linear (*Supporting Information* Table S1) and no O_2_^•−^ saturation behavior was observable.

Finally, the catalytic activities for the NiSOD model peptides obtained in this work were all in a similar range varying between 1.2 × 10^6^ (for **3**) and 3.5 × 10^6^ M^−1^ s^−1^ (for **7**, Table 2).

As discussed above, the NiSOD mimics were highly unstable and decomposed quickly upon treatment with KO_2_ resulting in a decreasing baseline which needs to be considered for kinetic analysis of the superoxide decay. Nevertheless, for higher peptide concentrations (>30 µM) decomposition of the Ni(II)-peptide complex was already visible within the first 500 ms of the superoxide decay traces which required a further reduction of the time window to 200–350 ms to minimize the impact of this process on the obtained catalytic activities (Supporting Information Fig. S31). Differences among the catalytic activities should not be considered as significant indicating a comparable activity for all NiSOD mimics tested in this work. In addition, we determined the catalytic activity of CuZnSOD (Table 2). The concentration of the CuZnSOD stock solution in phosphate buffer (150 mM, pH 8.0) was determined photometrically at 258 nm (ε = 10300 M^−1^ cm^−1^). The resulting correlation between *k*_*obs*_ and the enzyme concentration was perfectly linear (*Supporting Information* Table S1) yielding a catalytic activity close to the reported literature value (1.3 × 10^9^ M^−1^ s^−1^)^32^. In order to exclude “free” Ni(II) catalysis, the catalytic activity of NiCl_2_ was also assessed, which displayed no detectable activity.

Thus, our data indicate that neither the length of the respective model peptide (peptides **1**, **7** and **8**) nor the substitution of His1 (peptides **3** and **5**) or the modification of the imidazole ring of His1 (peptides **2** and **6**) had an impact on the catalytic activity. Only the acetylation of the N-terminus (peptide **4**) which results in a symmetric bis-amidate Ni(II) coordination had an impact on the catalytic activity yielding an inactive NiSOD mimic.

Regarding the role of the N-terminal histidine our data clearly indicate that the catalytic activity is not altered when His1 is replaced with Ala or Gln (**3** and **5**, Table 2). In contrast, His1 was found to be essential to retain the high catalytic activity of the native enzyme^8,9^. It is important to note that the NiSOD H1Q^8^ and H1A^17^ mutants were not totally inactive retaining a catalytic activity close to that of Nim^7^SOD H1Q (**5**) and the other NiSOD model peptides (Table 2). This indicates that the enzyme is also capable to degrade superoxide even without the imidazole ring of His1 suggesting a mechanistic change upon the loss of imidazole ring, which causes a significantly lower performance^17^.

**Figure 4:**
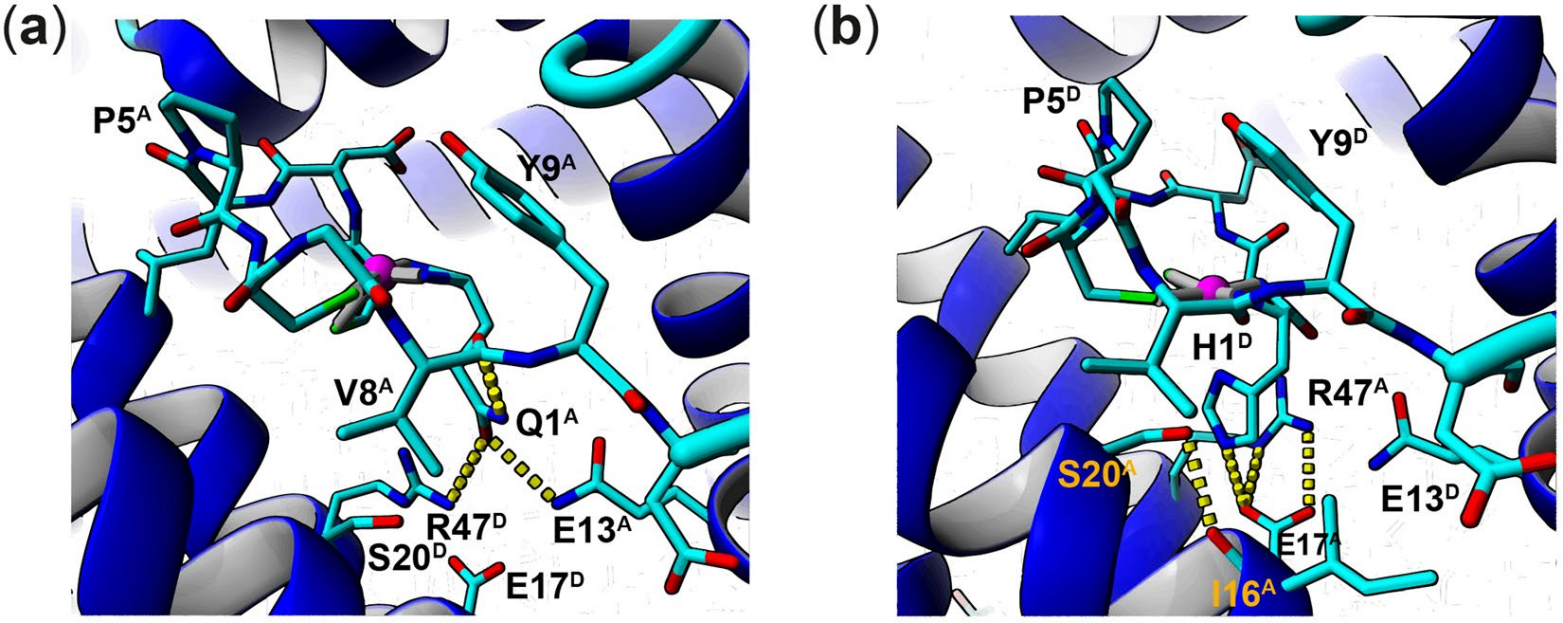
Molecular structure of the Ni(II) - active site of (**a**) NiSOD H1Q and (**b**) wt. NiSOD derived from molecular dynamics simulations showing the N-terminal residues 1–10 (stick representation, hydrogens are omitted for clarity, Ni-coordinating bonds – gray line, carbon – cyan, nitrogen – blue, oxygen – red, Ni(II) – magenta) and the hydrogen bond (yellow dotted line) network between a) E13, R47 and Q1 and (**b**) E17, R47 and H1. Superscript letters indicate the NiSOD subunit.

To clarify whether the Gln side chain of the NiSOD H1Q mutant can act as an axial ligand as molecular dynamics simulations were performed. We analyzed the hydrogen bond network of Gln1 versus His1 to answer the question whether Gln would be locked in a similar position as the His imidazole side chain in the wild type (wt) enzyme. From the simulation data side chain oxygen Oε2 of Glu17 from an adjacent subunit was identified as the main hydrogen bonding partner for the imidazole nitrogen Nε2 of His1 (H-bond probability of 0.824, Table 3, Fig. 4b) retaining the hydrogen bond triad present in the NiSOD crystal structure. Furthermore, after some fluctuations of the imidazole ring between a coordinating (His-on) and non-coordinating (His-off) orientation during the first 20 ns Nδ1 of His1 remains in the His-on position with an average Ni(II) - Nδ1 distance of 2.37 Å ± 0.13 Å (Supporting Information Fig. S32e). In contrast, the side chain oxygen of Gln1 (Oε1) was tightly hydrogen bound to Nε2 of Gln13 (same subunit) pointing away from the Ni(II) ion (Fig. 4a) almost throughout the entire simulation (H-bond probability of 0.912, Table 3, Fig. 4a) causing significant structural changes to the active site geometry (Supporting Information Fig. S32c). Moreover, calculated crystallographic b-factors (Supporting Information Fig. S32a and b) indicate an even lower mobility of Gln1 side chain compared to the imidazole of His1.

**Table 3:** Hydrogen bond partner and binding probability of Gln1 in NiSOD H1Q and His1 in wt. NiSOD derived from molecular dynamics simulations. Superscript letters indicate the respective NiSOD subunit.

	Oε1 **Gln1** ^A^		Nε2 **Gln1** ^A^			Nε2 **His1** ^D^		
H-bond partner	Nε2 Gln13^A^	NH2 Arg47^D^	Oε1 Glu17^D^	Oγ Ser20^D^	O Val8^A^	Oε2 Glu17^A^	Oγ Ser20^A^	O Ile16^A^
H-bond probability	0.912	0.087	0.053	0.207	0.303	0.824	0.019	0.001

In conclusion, it seems to be unlikely, that the Gln1 side chain can act as an axial ligand rationalizing the residual catalytic activity of the NiSOD H1Q mutant.

### First principles calculations

First principles calculations were employed in order to understand the fundamental differences between peptides **1** and **4** including different His side chain orientations and backbone amide protonation state in these calculations (*Supporting Information* Table S2). The orientation of the imidazole moiety of His1 towards the central Ni(II) (His1-in conformation) is energetically slightly preferred (see *Supporting Information* Table S3) over the His1-out conformation in all cases even without a direct Ni(II)-coordination (Nδ1-Ni distance always larger than 3.5 Å). Preliminary calculations of the corresponding triplet states of **1** were always about 12 kcal/mol higher in energy and in agreement with experimental observations reported earlier by us and others (data not shown)^13,14^. However, for peptide **4** the triplet state was only about 2 kcal/mol (3 kcal/mol in the gas phase) higher in energy than the singlet state resulting in a change of the square pyramidal coordination to a tetrahedral one. In this tetrahedral Ni(II)-complex, the acetylated N-terminus was no longer coordinating the central Ni(II)-ion and was replaced by the oxygen atom of the Leu4-Pro5 *trans*-peptide bond. Because there is no experimental evidence for such a paramagnetic complex, we have only considered the singlet state structures in this study. The calculations performed in vacuum and solvent resulted in very similar structures as well as relative energy values.

**Figure 5:**
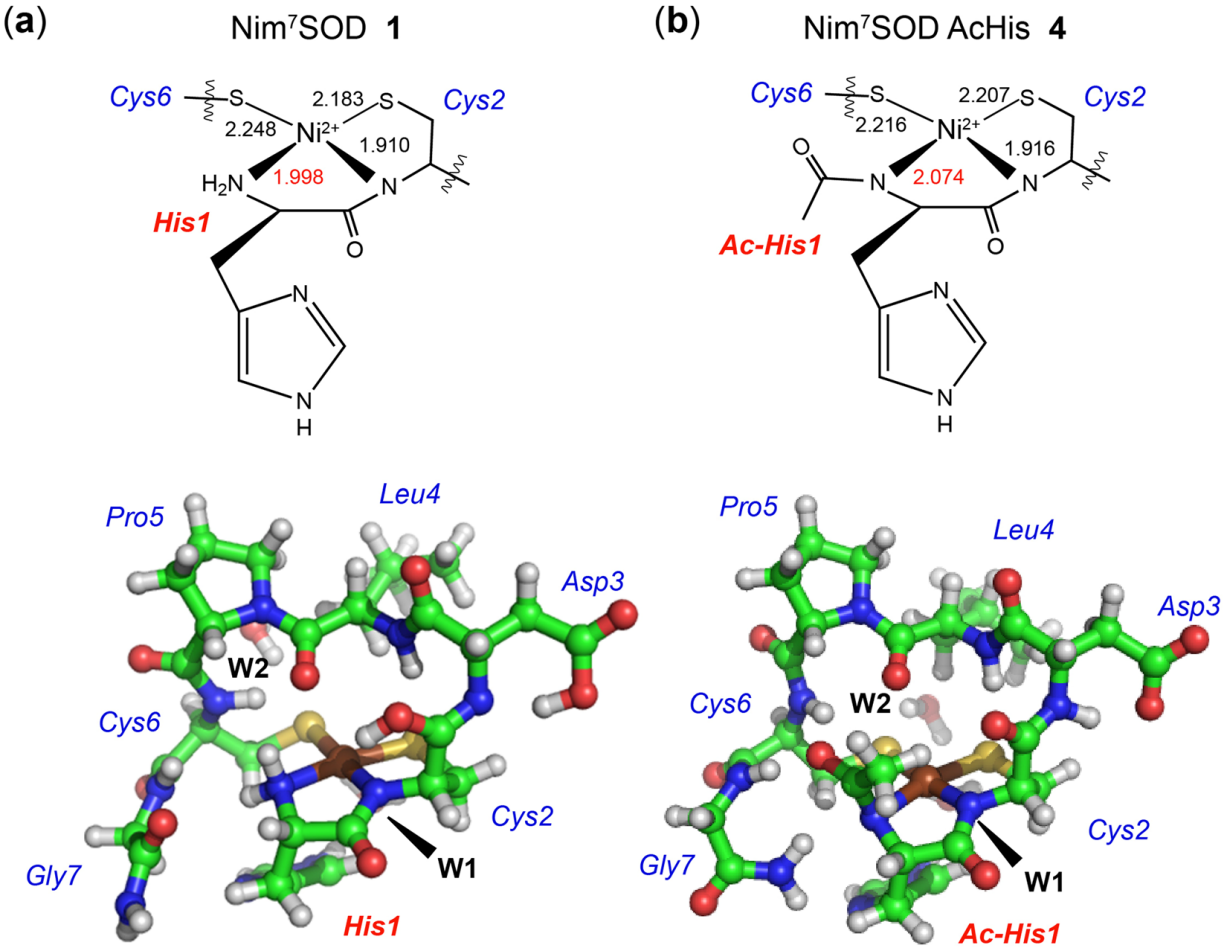
Structurally optimized peptides **1** (**a**) and **4** (**b**) with His1 oriented towards Ni(II) and the amide group deprotonated at BP86-D(MARIJ)/cc-pVTZ level of theory (color code: Ni - brown, carbon - green, sulfur - yellow, oxygen - red, nitrogen – blue, water molecules are indicated as W1 and W2).

According to our calculations the square planar Ni(II)-complex of the bis-amidate model peptide **4** is structurally more symmetric than peptide **1** revealing an elongated bond between the nitrogen of the acetylated amide group and the Ni(II) by 0.06 to 0.08 Å compared to the amine-Ni(II) distance in peptide **1** (Fig. 5, Supporting Information Table S4). In contrast to this the other nickel coordinating bonds are only weakly affected by the acetylation of the N-terminus (Fig. 5, Supporting Information Table S4). The variation of the protonation state of the amides (His1, Cys2, Supporting Information Table S2) leads only to small changes in the Ni-amide bond length (Supporting Information Table S4). The hydrogen bond networks of the ligands with the peptide and water environment were similar for both peptides **1** and **4**. Small differences occurred such as that for peptide **1** the S(Cys6)-HN(backbone) bond was stronger than the S(Cys2)-HN(backbone) bond, whereas the opposite was found for peptide **4**. Furthermore, the hydrogen bond of the Ni-coordinating N-terminus and the oxygen atom of the Leu4-Pro5 *trans*-peptide bond of peptide **1** changed to a hydrogen bond of the methyl group of the Ni-coordinating acetylated N-terminus and the oxygen atom of the Leu4-Pro5 *trans*-peptide bond of peptide **4** which slightly increases in strength (decreases in distance) when the amide group is not protonated. In the case of two deprotonated amide groups, the methyl group of the Ni-coordinating acetylated N-terminus is slightly orientated away from the oxygen atom resulting in weaker hydrogen bonds than in all other model peptides. Supporting Information Table S5 and Supporting Information Fig. S33 (Supporting Information) provides the bond length of the hydrogen bonds formed with the ligands coordinated to Ni(II).

Due to the relatively small structural differences between **1** and **4** and with respect to our LC-MS analysis of the oxidation products, test calculations of the S-oxidation of the Cys6 unit towards the sulfoxide (Cys6-SO) and sulfone (Cys6-SO_2_) for peptides **1** and **4** were performed. Here, we found that for all cases the sulfur oxidation was energetically preferred (Supporting Information Table S6). Moreover, the sulfur oxidation to sulfoxide and sulfone for peptides **1** and **4** was energetically (reaction energy) comparable within the accuracy of the method with a slight preference of the oxidation to sulfoxide or sulfone for peptide **4**. Therefore, the sulfone formation of **4** must be somehow hindered to agree with experimental data and the determined half-life of peptide **1** and **4**.

## Discussion

### Stability of the NiSOD mimics

As indicated by our LC-MS analysis, the addition of KO_2_ or O_2_ to the NiSOD model peptides **1**–**8** leads to a significant sulfur-based oxidation. Similar oxidation products were observed for numerous inorganic NiSOD model complexes^33–36^. The fast superoxide decay in the presence of the Ni(II)-peptides indicates that O_2_ and/or H_2_O_2_ produced by the Ni(II)-peptides and not KO_2_ is responsible for their degradation. Regarding the exclusive observation of disulfides upon air exposure of **7** (Supporting Information Fig. S10) and KO_2_ treatment of the linear, Ni(II)-free peptide **1** (Supporting Information Fig. S27) the oxygenation products are most likely caused by hydrogen peroxide and further depend on the presence of Ni(II) in solution. The strongly reduced amounts of oxygenated products observed upon KO_2_-induced Ni(II)-peptide degradation in the presence of the radical scavenger ascorbic acid might be indicative for a radical-based Fenton-like oxygenation mechanism. In this case, hydroxyl radicals which are formed by the decomposition of a Ni(II)-peroxo complex would be quenched by ascorbic acid leaving only oxygen-induced peptide degradation products. Alternatively, ascorbic acid would also be able to directly reduce hydrogen peroxide preventing the formation of the afore-mentioned Ni(II)-peroxo complex.

According to our stability measurements peptide **4** was indicated to be far more stable against oxidation than the other model peptides. This was rather surprising, considering a recent study in the literature, which reported that the bis-amidate Ni-peptide [Ni^II^(SOD^M1^-Ac)] was found to quickly oxidize in contrast to the fully stable amine/amide NiSOD mimic [Ni^II^(SOD^M1^)]^13,20^. Based on these findings the authors concluded that the amine/amide Ni(II)-coordination must be the key factor for the stability and catalytic activity of the model peptides as well as for the enzyme identifying the degradation of [Ni^II^(SOD^M1^-Ac)] to be the reason for its inactivity^13,20^.

Our DFT calculations of the native-like amine/amide NiSOD mimic and the bis-amidate Nim^7^SOD AcHis might explain their different stabilities against oxidation by a slightly more symmetric square planar coordination geometry of the later one. Furthermore, electronic changes resulting from the more symmetric Ni(II) coordination environment of **4** might reduce the ability to coordinate any axial ligands, such as superoxide or oxygen, thus being less susceptible for oxidation, but catalytically inactive. The HOMO, HOMO-1 and HOMO-2 (which are energetically similar and can change in order for different peptide models) for peptides **1** and **4** mainly contain sulfur p-orbitals and Ni 3d-orbital contributions. Compared to peptide **1**, the sulfur orbital contribution slightly decreases, whereas the Ni orbital contribution slightly increases for the sum of the three orbitals for peptide **4**.

These findings are in line with DFT calculations performed by Neupane and Shearer comparing the two analogous NiSOD model peptides [Ni^II^(SOD^M1^)] (analogous to **1**) and [Ni^II^(SOD^M1^-Ac)] (analogous to **4**) unveiling a similar decrease of S-character and increase of Ni-character in the HOMO when the Ni(II)-coordination was changed from the amine/amide to the bis-amidate motif^26^. Furthermore and in line with experimental observations, Fiedler *et al*. found for some Ni(II)-S_2_N_2_ complexes that the exchange of neutral amine ligands to anionic amide ligands had a similar effect with respect to the S- and Ni-contribution to the HOMO resulting in protection from S-oxidation^37^.

Besides the amine vs. amide coordination, the hydrogen bond network around the NiSOD active site is considered to play an important role in preventing sulfur-based ligand oxidation in various studies on non-peptidic model systems^21,36,38^. Especially, hydrogen bonds to the Ni(II)-ligating sulfur atoms are present in some Ni(II)-N_2_S_2_ complexes. They were shown to result in stabilization of S-based MOs relative to Ni-based AOs and protection against S-oxidation^21,36^. However, the peptidic NiSOD models **1**–**8** did not appear to oxidize slower than the non-peptide based compounds^21,36^ even though the hydrogen bond networks of the peptide backbone in **1** and **4** resemble that of the NiSOD active site unveiling hydrogen bonds between backbone amides and the sulfur atoms of Cys2 and Cys6 (Supporting Information Fig. S33). Consequently, our results indicate that the nature of the nitrogen donor (amine/amide vs. bis-amidate) mainly dictates sulfur reactivity in the model peptides. Furthermore, the H-bond network to the sulfur atoms of the thiolate ligands might be less significant in preventing S-oxidation in the NiSOD enzyme and point towards more sterical reasons. Otherwise, a much higher stability of the peptidic NiSOD model systems than for the non-peptidic NiSOD model systems should be observed.

The catalytic inactivity of **4** observed in this work confirms earlier results^26^. Regarding our DFT-optimized structures of **1** and **4** the only structural difference which could potentially explain the inactivity of **4** might be associated with the absence of the N-terminal protons (Fig. 5) in **4**. This suggestion is further supported by the work of Nakane and coworkers who isolated a mixed amine/amide Ni(II)-N_2_S_2_-superoxo complex, in which the coordinating superoxide was found to be significantly stabilized by the proton of the amine ligand^38^. Moreover, Nakane’s Ni-complex showed some superoxide reactivity in the presence of a proton source^38^. Interestingly, it appears to be the only non-peptidic NiSOD model complex exhibiting such a single protonated amine ligand within a mixed amine/amide Ni(II) environment with respect to the Ni-complexes reviewed for this study^19,33–35,38^. This clearly implies a key role of the N-terminal protons for catalytic activity which possibly explains the inactivity of the non-peptidic NiSOD models with just one exception.

With respect to the highly unstable NiSOD mimetic peptides we optimized the sample preparation procedure and data analysis of the kinetic measurements resulting in high quality kinetic data. As a result, our activity data revealed very similar catalytic activities for amine/amide NiSOD mimics which were about three orders of magnitude smaller than for the wild type enzyme^8,39^.

As outlined in Table 2 the catalytic activities for NiSOD^M1^ and mimics derived thereof are more than an order of magnitude higher than the value reported herein^15^. Moreover, the catalytic activity for Nim^12^SOD^M1^-tos was found to be almost 2 orders of magnitude higher  compare to our slightly shorter Nε-tosylated Ni-peptide (**6**, Table 2)^15^. Only, the Nε-methylated Ni(II)-peptides NiSOD^M1^ and Nim^7^SOD H1H′ reported in this work were about equally active^9^. As described above, the instability of the NiSOD model peptides which was not considered in previous studies strongly influences the fitting routine of the superoxide decay traces and can significantly alter the resulting catalytic activities, thus most likely being the reason for the observed differences to previous works. Nevertheless, the use of phosphate buffer in our studies rather than NEM buffer could have a potential effect on the catalytic activities of the model peptides even though it seems to be un-likely, that this effect will increase the activities for some orders of magnitude.

With respect to the similar catalytic activities of the Ni(II)-peptides **3** and **5** compared to **1** it seems that an axial imidazole ligand is not required in order to catalytically degrade superoxide by the NiSOD model peptides. As already mentioned earlier, the H1Q and H1A mutants of the wild type NiSOD enzyme showed residual catalytic activity which was in the range of the NiSOD model peptides^17^. As indicated by our MD simulations NiSOD H1Q also lacks an axial ligand as well as the NiSOD H1A mutant^17^ and would therefore be analogous to the Ni(II)-peptides **3** and **5**. Therefore, we postulate that an alternative superoxide degradation pathway might exist, if no axial imidazole ligand is present.

Also, the modifications of the imidazole ring with different Lewis basic substituents at Nε (-methyl **2**, -tosyl **6**, Table 2) of H1 had no impact on the catalytic activity, thus supporting our conclusion that the imidazole ring is not involved in the catalytic superoxide degradation by the NiSOD model peptides. Moreover, the NiSOD model peptides are not suitable to probe for electronic effects on the axial imidazole introduced by the hydrogen bond network between His1, Glu17 and Arg47 in the native enzyme (Fig. 4b). Consequently, this supports our hypothesis of two possible superoxide degradation pathways depending on the presence of an imidazole moiety. Interestingly, Shearer *et al*. also recently suggested a different mechanism of superoxide degradation for the Ni(II)-model peptides^12,40^.

The remaining question is, whether the hydrogen bond network indeed fine-tunes the electronic properties of His1 thus being responsible for the much higher catalytic activity of the native NiSOD. Our observations along with the enzyme mutation data^8,9,17^ indicate that the hydrogen bond network mainly orients the imidazole ring of His1 enforcing a square pyramidal-like coordination environment which then helps to stabilize the Ni(III) oxidation state.

### Mechanistic Implications

**Figure 6:**
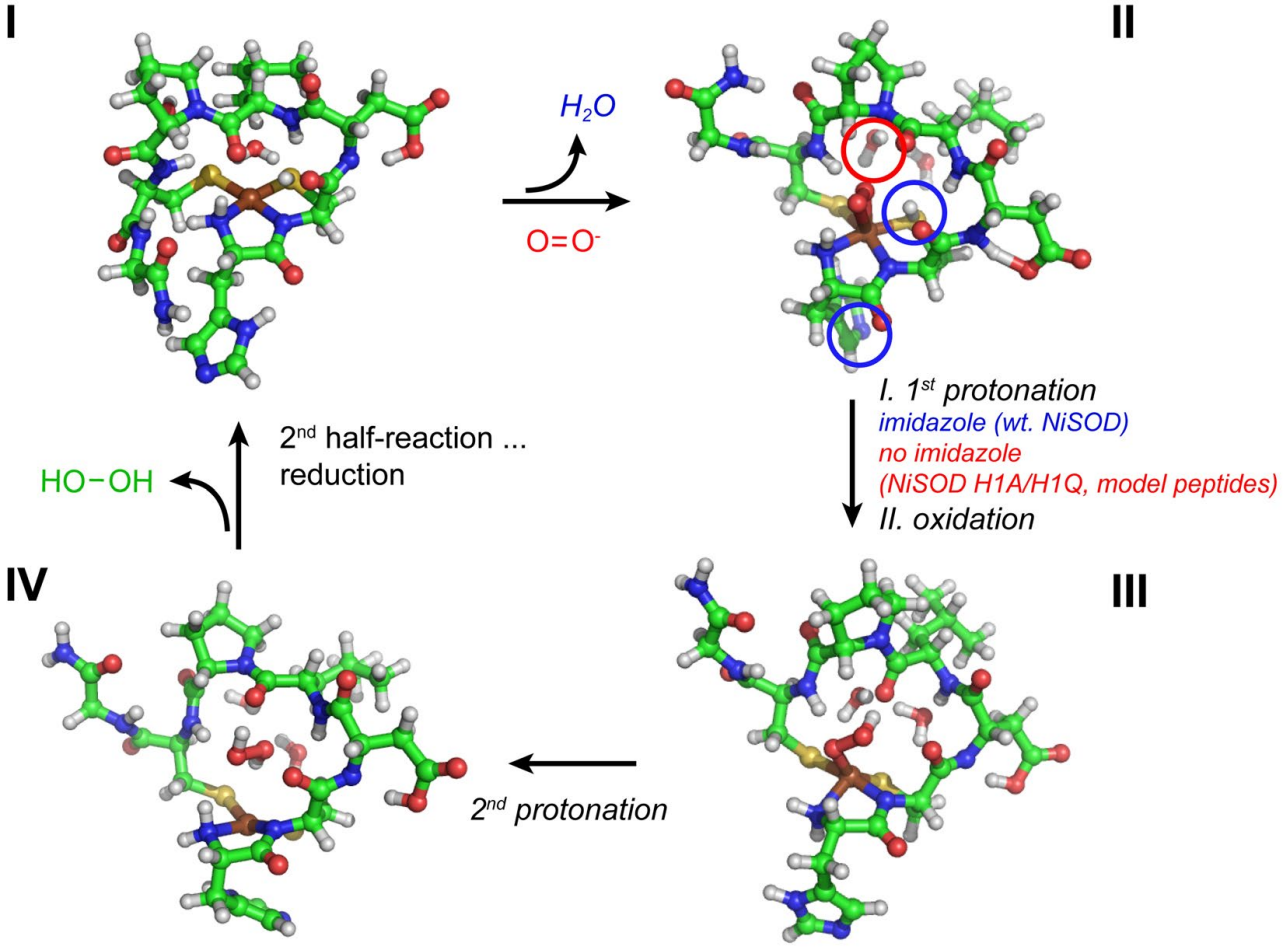
Proposed mechanism of the first half-reaction of superoxide degradation by NiSOD and peptide-based model system. Possible proton sources are highlighted (step II) whether a pre-oriented imidazole (towards Ni(II)) is present (indicated in blue) or not (indicated in red), indicating two superoxide degradation pathways with a significantly lower performance for the model peptides and His1-deficient NiSOD mutants, which lack the pre-oriented imidazole moiety (color code according to Fig. 5).

Regarding the role of the N-terminal histidine, our data unambiguously show, that His1 is not required to yield catalytically active peptides. However, His1 is important for the high catalytic activity of the native enzyme, which decreases to a similar level as for the NiSOD model peptides when His1 is mutated to Gln or Ala suggesting that the mechanism of superoxide degradation might proceed via different mechanisms. Besides the stabilizing effect on the Ni(III) oxidation state through the imidazole side chain of His1 in the wild type NiSOD, another possible reason for the lower performance of the NiSOD model peptides and some His1 deficient NiSOD mutants (H1Q and H1A) might be associated with the source of the protons for hydrogen peroxide formation. As earlier concluded by Bryngelson and co-workers from pH dependent measurements of the catalytic superoxide degradation of wild type NiSOD, the proton is provided through the protein rather than the bulk water^8,17^. Interestingly and in contrast to the wild type enzyme their NiSOD H1Q mutant showed an almost linear pH dependent catalytic performance, which might point towards bulk water as the proton source^8^. Moreover, water as the source of the proton in the NiSOD model would also be in line with earlier first principle calculations by us on a possible substrate position, which only showed agreement to experimental REDOR distances in the presence of one water molecule at a specific position to stabilize the substrate (Fig. 6). Regarding the proton source of the enzyme, recent literature data suggest a protonation site on either of the two Ni-coordinating sulfur atoms of Cys2 or Cys6 (Fig. 6) which must exhibit an unusually high pk_a_ of >8.5 with respect to the pH dependence of the catalytic superoxide degradation, thus not completely ruling out His1 as a proton source^40–42^.

## Conclusion

For the first time, the oxidation stability of different NiSOD model peptides, which vary in length and the Ni(II) coordination environment were systematically studied and correlated with their catalytic activities unveiling their high oxidation sensitivity. In contrast to literature reports, our stopped-flow kinetic data revealed about an order of magnitude higher stability of the catalytically inactive bis-amidate NiSOD model peptide in comparison to the catalytically active amine/amide NiSOD mimics in the presence of KO_2_. Therefore, the amine/amide Ni(II) coordination is clearly the key factor for catalytic activity as concluded in an earlier study^26^ but at the same time results in a much higher oxidation sensitivity at least for the model peptides. In depth LC/MS analysis on the nature of the peptide degradation products might point towards a Fenton-like degradation mechanism. Our stopped-flow data on the catalytic activity of a set of NiSOD model peptides confirmed earlier findings obtained with other methods and solves a current dispute in the literature^13,15–17^, regarding the role of the N-terminal histidine (H1). Finally, our data clearly demonstrate that the N-terminal histidine is not required for the catalytic activity of the NiSOD model peptides suggesting a different catalytic mechanism for the superoxide degradation.

## Methods

### Syntheses of m^7^SOD, m^9^SOD, m^12^SOD, m^7^SOD AcHis, m^7^SOD H1X (X = A, Q, H′, H-tos where H′ = L-Nε-methyl-His, H-tos = L-Nε-tosyl-His)

If not otherwise stated all amino acid derivates, coupling reagents and resins were purchased from Orpegen^®^ Pharma and Novabiochem, respectively, and used without further purification. The peptides were synthesized using a standard Fmoc-SPPS protocol on Rink amide MBHA resin (4-(2′,4′-dimethoxyphenyl-Fmoc-aminomethyl)-phenoxyacetamido-norleucyl-4-methylbenzhydrylamine) with a loading capacity of 0.58 mmol/g and 1-[Bis(dimethylamino)methylene]-1H-1,2,3-triazolo[4,5-b]pyridinium 3-oxid hexafluorophosphate (HATU, 4 equiv. relative to loading capacity) or diisopropylcarbodiimide (DIC, 4 equiv. relative to loading capacity) as coupling reagents. The base diisopropylethyl amine (DIEA) was used in 2-fold excess compared to amino acids and coupling reagents. Cleavage from the resin was achieved with 95% trifluoracetic acid (TFA), 2.5% triisopropyl silane and 2.5% water (95% TFA, 2.5% triisopropyl silane, 2.5% ethanedithiol for **6**) for three hours. The peptides were precipitated in cold diethyl ether, washed several times and freeze-dried prior to purification by semi-preparative HPLC and stored at −28 °C. Peptides were characterized by HPLC and ESI-MS (Table 4, see Supporting Information Figs S1–S8).

**Table 4:** Overview of peptides synthesized in this work.

peptide		sequence
m SOD	**1**	HCDLPCG-NH_2_
m^7^SOD H1H′	**2**	(Nε-methyl-His)CDLPCG-NH_2_
m SOD H1A	**3**	ACDLPCG-NH_2_
m SOD AcHis	**4**	(Nα-acetyl-His)CDLPCG-NH_2_
m^7^SOD H1Q	**5**	QCDLPCG-NH_2_
m^7^SOD H1H-tos	**6**	(Nε-tosyl-His)CDLPCG-NH_2_
m^9^SOD	**7**	HCDLPCGVY-NH_2_
m^12^SOD	**8**	HCDLPCGVYDPA-NH_2_

### Analytical data of the linear peptides

**1** m^7^SOD: HPLC: R_t_ = 3.2 min (analytical), MS ESI (pos. ion), m/z: 743.29629 [M + H]^+^, 372.15204 [M + 2H]^2+^, calc. monoisotopic mass (m.i.): 743.29634 [M + H]^+^

**2** m^7^SOD H1H′: HPLC: R_t_ = 3.2 min (analytical), MS ESI (pos. ion), m/z: 757.31192 [M + H]^+^, 379.15986 [M + 2H]^2+^, calc. m.i.: 757.31199 [M + H]^+^

**3** m^7^SOD H1A: HPLC: R_t_ = 3.0 min (analytical), MS ESI (pos. ion), m/z: 677.27401 [M + H]^+^, 339.14076 [M + 2H]^2+^, calc. m.i.: 677.27454 [M + H]^+^

**4** m^7^SOD AcHis: HPLC: R_t_ = 3.4 min (analytical), MS ESI (pos. ion), m/z: 785.30632 [M + H]^+^, 393.15704 [M + 2H]^2+^, calc. m.i.: 785.30691 [M + H]^+^

**5** m^7^SOD H1Q: HPLC: R_t_ = 3.1 min (analytical), MS ESI (pos. ion), m/z: 734.29568 [M + H]^+^, 367.65165 [M + 2H]^2+^, calc. m.i.: 734.29601 [M + H]^+^

**6** m^7^SOD H1H-tos: HPLC: R_t_ = 6.2 min (analytical), MS ESI (pos. ion), m/z: 897.30321 [M + H]^+^, 449.15618 [M + 2H]^2+^, calc. m.i.: 897.30519 [M + H]^+^

**7** m^9^SOD: HPLC: R_t_ = 4.9 min (analytical), MS ESI (pos. ion), m/z: 1005.42767 [M + H]^+^, 503.21800 [M + 2H]^2+^, calc. m.i.: 1005.42808 [M + H]^+^

**8** m^12^SOD: HPLC: R_t_ = 5.3 min (analytical), MS ESI (pos. ion), m/z: 1288.54436 [M + H]^+^, 644.77633 [M + 2H]^2+^, calc. m.i.: 1288.54490 [M + H]^+^

### Synthesis of Nim^7^SOD, Nim^9^SOD, Nim^12^SOD, Nim^7^SOD H1X, Nim^7^SOD AcHis

All aqueous solutions were degassed through at least three freeze-pump-thaw cycles under Ar atmosphere. Peptide and Ni-peptide stock solutions were generally prepared under inert conditions.

Prior to the addition of Ni(II), peptide solutions were prepared from phosphate buffer (150 mM KH_2_PO_4_/K_2_HPO_4_, pH 8.0). Ni(II) (NiCl_2_ × 6H_2_O, 99.999%, Sigma Aldrich, St. Louis, MO, USA) was added from an aqueous stock solution (0.254 M) according to 75% of the peptide sample weight and the pH of the resulting Ni(II) peptide solution was re-adjusted if required using 1 M NaOH. Formation of the Ni(II)-peptide complex was assessed through mass spectrometry and UV-Vis spectroscopy on a TIDAS S spectrometer (J&M Analytik, Essingen, Germany).

**1** Nim^7^SOD: MS ESI (pos. ion), m/z: 799.21437 [M + H]^+^, 400.11072 [M + 2H]^2+^, calc. monoisotopic mass (m.i.): 799.21637 [M + H]^+^, UV/Vis (150 mM KH_2_PO_4_/K_2_HPO_4_, pH 8.0): λ_max_ (ε), 260 (17682 M^−1^ cm^−1^), 460 (280)

**2** Nim^7^SOD H1H′: MS ESI (pos. ion), m/z: 813.23123 [M + H]^+^, 407.11962 [M + 2H]^2+^, calc. m.i.: 813.23168 [M + H]^+^, UV/Vis (150 mM KH_2_PO_4_/K_2_HPO_4_, pH 8.0): λ_max_ (ε), 260 (14553 M^−1^ cm^−1^), 460 (500)

**3** Nim^7^SOD H1A: MS ESI (pos. ion), m/z: 733.19263 [M + H]^+^, 367.09989 [M + 2H]^2+^, calc. m.i.: 733.19424 [M + H]^+^, UV/Vis (150 mM KH_2_PO_4_/K_2_HPO_4_, pH 8.0): λ_max_ (ε), 260 (15948 M^−1^ cm^−1^), 460 (533)

**4** Nim^7^SOD AcHis: MS ESI (pos. ion), m/z: 841.22630 [M + H]^+^, 421.11703 [M + 2H]^2+^, calc. m.i.: 841.22660 [M + H]^+^, UV/Vis (150 mM KH_2_PO_4_/K_2_HPO_4_, pH 8.0): λ_max_ (ε), 290 (7626 M^−1^ cm^−1^), 318 (sh), 507 (927)

**5** Nim^7^SOD H1Q: MS ESI (pos. ion), m/z: 790.21500 [M + H]^+^, 395.61172 [M + 2H]^2+^, calc. m.i.: 790.21570 [M + H]^+^, UV/Vis (150 mM KH_2_PO_4_/K_2_HPO_4_, pH 8.0): λ_max_ (ε), 260 (11627 M^−1^ cm^−1^), 460 (167)

**6** Ni m^7^SOD H1H-tos: MS ESI (pos. ion), m/z: 953.22406 [M + H]^+^, 477.11620 [M + 2H]^2+^, calc. m.i.: 953.2249 [M + H]^+^, UV/Vis (150 mM KH_2_PO_4_/K_2_HPO_4_, pH 8.0): λ_max_ (ε), 260 (21180 M^−1^ cm^−1^), 473 (184)

**7** Nim^9^SOD: MS ESI (pos. ion), m/z: 1061.34602 [M + H]^+^, 531.17757 [M + 2H]^2+^, calc. m.i.: 1061.34778 [M + H]^+^, UV/Vis (150 mM KH_2_PO_4_/K_2_HPO_4_, pH 8.0): λ_max_ (ε), 260 (16857 M^−1^ cm^−1^), 460 (242)

**8** Nim^12^SOD: MS ESI (pos. ion), m/z: 1366.44654 [M + Na]^+^, 694.71813 [M + 2Na]^2+^, calc. m.i.: 1366.44654 [M + Na]^+^, UV/Vis (150 mM KH_2_PO_4_/K_2_HPO_4_, pH 8.0): λ_max_ (ε), 260 (11986 M^−1^ cm^−1^), 460 (204)

### LC/MS sample preparation

A 1 mM solution of Ni(II)-peptide complex was prepared under inert conditions in phosphate buffer (150 mM, pH 8.0) and treated with a 10fold excess of solid KO_2_. For the radical scavenger experiments a 10fold excess of ascorbic acid was added to the peptide solution prior to the 10fold KO_2_ addition. The solution was left for about 10 to 15 min before LC/MS analysis. Prior to the LC/MS measurements a small sample was taken from the Ni(II)-peptide/KO_2_/buffer solution and diluted (1:10, v-v) with aqueous TFA (0.1%, v-v).

### Analytical characterization of the peptides

#### HPLC

All crude peptides were purified by semi-preparative RP-HPLC using a Waters 600 system (Waters, Milford, MA, USA) equipped with a C18 column (MultoKrom 100–5 C18, 5 µm particle size, 100 Å pore size, 250 × 20 mm, CS Chromatographie Service, Langerwehe, Germany). The gradient elution system was 0.1% trifluoroacetic acid (TFA) in water (eluent A) and 0.1% TFA in acetonitrile (eluent B). The peptides were eluted with a gradient of 5–40% eluent B in 60 min and a flow rate of 8 mL/min. The peaks were detected at 220 nm. Collected fractions were combined, freeze-dried and stored at −28 °C. Purity was confirmed by analytical RP-HPLC on a Waters XC e2695 system (Waters, Milford, MA, USA) employing a Waters PDA 2998 diode array detector or a Waters 600 system employing a Waters 996 PDA detector. Both systems were equipped with a Multospher 120 RP 18 HP column (C18, 3.1 µm particle size, 120 Å pore size, 60 × 4.6 mm, CS Chromatographie Service, Langerwehe, Germany). The peptides were eluted with a gradient of 5–40% eluent B in 10 min at a flow rate of 2 mL/min. Chromatograms were extracted at 214 nm.

In order to avoid sample oxidation during HPLC sample preparation and measurements Ni(II)-peptide/buffer solution was acidified by diluting the sample 1:10 with aqueous TFA (1% or 0.1%, v-v), which effectively slows down the oxidation process allowing for reliable HPLC analysis.

#### Mass and LC/MS spectrometry

The molecular weight of the purified and oxidized peptides was confirmed by ESI mass spectrometry on a micrOTOF-Q III or TOF-Q impact II spectrometer (Bruker Daltonik GmbH, Bremen, Germany) and calibrated using Bruker’s ESI-Tune-Mix. LC/MS analysis was performed on a TOF-Q impact II spectrometer (Bruker Daltonik GmbH, Bremen, Germany) equipped with a Agilent 1200 HPLC system using a C18 column (Reprosil Gold 120, C18, 3.0 µm particle size, 120 Å pore size, 100 × 2 mm, Dr. Maisch GmbH, Ammerbuch-Entringen, Germany). The oxidized peptides were eluted with a gradient of 5–95% eluent B in 20 min at a flow rate of 0.2 mL/min. Chromatograms were extracted at 210–220 nm.

### Stopped-flow experiments

To assess the stability and catalytic activity of the Ni(II)-peptide complexes stopped-flow measurements were conducted on a HI-TECH SF-61 DX2 stopped flow instrument (TgK Scientific Ltd., Bradford-on-Avon, UK). Peptide/buffer and superoxide/DMSO solutions (50 mM KO_2_, 75 mM 18-crown-6) were mixed in a 10:1 ratio in single mixing mode. 18-crown-6 was used to increase the solubility of KO_2_ in DMSO. The mixing process between buffer and DMSO was measured as a background process and subtracted from all measurements. All measurements were performed at 25.0 °C (±0.2 °C).

To measure the stability of the NiSOD model peptides, the stopped-flow system was coupled to a photo diode array detector (TIDAS S, J&M Analytik, Essingen, Germany). UV/Vis spectra were recorded within the range of 200–800 nm and at an interval of 1 s for about 20 min (50 min for Nim^7^SOD AcHis). Integration time for each single spectrum (N = 3) was 33 ms (99 ms for Nim^7^SOD AcHis). The resulting spectra were processed with Panorama 3.1 (LabCognition – Analytical Software GmbH, Cologne, Germany).

The catalytic activity of the NiSOD model peptides was determined by observing the superoxide degradation at 250 nm (ε = 2000 l mol^−1^ cm^−1^) according to the method originally described by Riley *et al*.^30^. Therefore, the stopped-flow system was used in single wavelength mode employing a xenon lamp as the light source. The superoxide decay was typically observed for 1 to 4 seconds and five to ten superoxide decay traces were averaged and further analyzed according to the procedure described by Bolann *et al*.^31^. The first 5 ms of each decay curve were disregarded to further correct for optical disturbances resulting from the mixing of buffer and DMSO. Superoxide decay was measured at five different peptide concentrations (b) in phosphate buffer (150 mM, pH 8.0). Prior to each activity measurement, uncatalyzed superoxide decay was recorded in order to derive the 2^nd^ order rate constant of the self-disproportionation (*k*_2_) and the catalytic background activity (*k*_1_). The catalytic in-activity of NiCl_2_ was verified in a control experiment. Catalytic activity of CuZnSOD (Sigma Aldrich, St. Louis, MO, USA) was determined in order to test the overall performance of the setup.

Kinetic Studio 4.0 (TgK Scientific Ltd., Bradford-on-Avon, UK) was used for data acquisition, processing and kinetic analysis.

Catalytic rate constants *k*_*cat*_ for the NiSOD model peptides were calculated according to the rate law (eq. 2, a – superoxide concentration, *k*_*2*_ – 2^nd^ order rate constant for the self-disproportionation of superoxide, *k*_*obs*_ – observed rate constant of the catalyzed superoxide decay) of the catalyzed superoxide degradation (eq. 1) as described by Bolan *et al*.^31^. The first term in eq. 2 is the second order self-disproportionation of the superoxide, whereas the second term is the first order catalytic superoxide degradation. In praxis, the rate constant k_obs_ of the second term in eq. 2 consists of two rates *k*_*1*_ and *k*_3_ (eq. 4), whereas *k*_1_ is the 1^st^ order background superoxide decay induced by catalytically active impurities and *k*_3_ (=*k*_*cat*_) is the 2^nd^ order rate constant of NiSOD model peptide-induced superoxide decay which scales linearly with the concentration (*b*) of the SOD active model compound. According to eq. 4 *k*_3_ results from the slope of the linear fit when plotting *k*_*obs*_ and the concentration of the model peptide. The experimental superoxide decay can then be fitted by the integrated form of eq. 2 which is given as eq. 3 (C – offset).

1 $$2{{{\rm{O}}}_{2}}^{.-}+2{{\rm{H}}}^{+}\to {{\rm{H}}}_{2}{{\rm{O}}}_{2}+{{\rm{O}}}_{2}$$

2 $$-\,\frac{da}{dt}={k}_{2}{a}^{2}+{k}_{obs}a$$

3 $$a(t)=\frac{{a}_{0}{e}^{-{k}_{obs}t}}{(\frac{{k}_{2}}{{k}_{obs}}){a}_{0}(1-{e}^{-{k}_{obs}t})+1}+C$$

4 $${k}_{obs}={k}_{1}+b{k}_{3}$$

### DFT structure calculations

Density Functional Theory (DFT) calculations were performed using the Turbomole 6.6 software package^43^. Structure optimizations were done employing the BP86 functional and D3 Grimme dispersion correction as well as the Dunning triple-zeta basis set cc-pVTZ^44–49^. In order to speed the calculations up the multipole accelerated resolution of identity approximation (MARI-J) was used^50–52^. Test structure optimization calculations without this approximation resulted in negligible structural changes, this was also found for the total energies. Test single point calculations were carried out using a B3LYP and B3LYP* functional plus dispersion correction for B3LYP^48,53–56^. The B3LYP-D3 result differed from the BP86-D3 result by less than 1 kcal/mol in relative energy, the B3LYP* result by less than 4 kcal/mol. The qualitative results were not changed. Solvent effects (water) were simulated via the continuum solvation model (COSMO) using a dielectric constant of 80^57^. Structural changes of the optimized model peptides with and without considering solvent effects were very small, mostly total energies were shifted leading to small changes in relative energetics and reaction energies. Frequency calculations for the gas phase were performed and confirmed the calculated structures as minimum structures.

The DFT calculations were performed for the Nim^7^SOD (**1**) and Nim^7^SOD AcHis (**4**) model systems with seven amino acids, the active site Ni(II)-ion as well as two water molecules. The two water molecules were found to be important for stabilizing the cyanide anion in a peptide substrate model, thus these were added to this study at the position, where they were found to stabilize the cyanide ion to serve next to the solvent model as explicit solvent molecules^14^. As starting point for the calculations the solution NMR structure saturated with hydrogen atoms was employed. The amino and carboxyl groups in the peptide backbone have been protonated with respect to the acidity of the aqueous buffer solution with a pH value of 8.0 as used in the experiments. The side chain of aspartic acid is anionic, the coordinating amino group of His1 as well as the amino group of Gly7 is neutral, the imidazole ring of His1 is neutral and the cysteine’s 2 and 6 is anionic. Depending on the amide protonation the total charge results to be −1, −2 or −3. Due to the expected mobility of His1, the side chain was considered in an inside and outside configuration. Because there was no experimental evidence for a paramagnetic nature of the model systems in the NMR measurements, we considered all systems to be closed shell complexes.

### Molecular dynamics simulations

The crystal structure of *S. coelicolor* NiSOD (pdb ID 1T6U) was used as a starting structure where H1 was mutated to Q in one of the six identical subunits.

Energy minimization was done prior to the molecular dynamics simulation in explicit water. Charged amino acids were assigned according to the predicted pKa of the amino acid side chains by Ewald summation^58^ and were neutralized by adding counter ions (NaCl). Ni^2+^ was modeled according to the procedure described by Li *et al*.^59^.

To gain performance, a multiple time step algorithm together with a simulation time step interval of 2.5 fs was chosen with a dodecahedral simulation cell^60,61^.

Unless otherwise stated TIP3P water model and the Yasara2 force field^62^ were used for energy minimization and molecular dynamics simulations^63,64^ employing the PME method^65^ to describe long-range electrostatics at a cut-off distance of 8 Å at physiological conditions (0.9% NaCl, 298 K, pH 7.4^58^).

Molecular graphics were created with YASARA (www.yasara.org) and POVRay (www.povray.org).

### Data availability

The datasets generated during and/or analyzed during the current study are available from the corresponding author on reasonable request. xyz coordinates of the DFT optimized structural models of peptide **1** and **4** generated in this work are provided in *Supporting Information* Table S7.

## Electronic supplementary material

Supplementary Information
