# Supplementary material for: New insights into the mechanism of nickel superoxide degradation from studies of model peptides
Source: Sci Rep. 2017 Dec 8;7:17194. doi: 10.1038/s41598-017-17446-3 (PMC5722923; doi:10.1038/s41598-017-17446-3)
Supplement: Supplementary file 1 — Supplementary Information [file 41598_2017_17446_MOESM1_ESM.pdf]

Supporting information

New insights into the mechanism of nickel superoxide degradation from studies of model peptides

Daniel Tietze,[1]* Jana Sartorius,[1] Banabithi Koley Seth,[1] Kevin Herr,[1] Pascal Heimer,[2] Diana Imhof,[2] Doreen Mollenhauer[3] and Gerd Buntkowsky[1]*

[1]Eduard-Zintl Institute for Physical and Inorganic Chemistry, Darmstadt University of Technology, Alarich-Weiss-Str. 8, 64287 Darmstadt, Germany, [2]Pharmaceutical Biochemistry and Bioanalytics, Pharmaceutical Institute, University of Bonn, An der Immenburg 4, D-53119 Bonn, Germany, [3]Institute of Physical Chemistry, Justus Liebig University Giessen, Heinrich-Buff-Ring 17, D-35392 Giessen, Germany

1. Analytical data for the NiSOD model peptides

HPLC chromatograms and mass spectra of the linear peptides

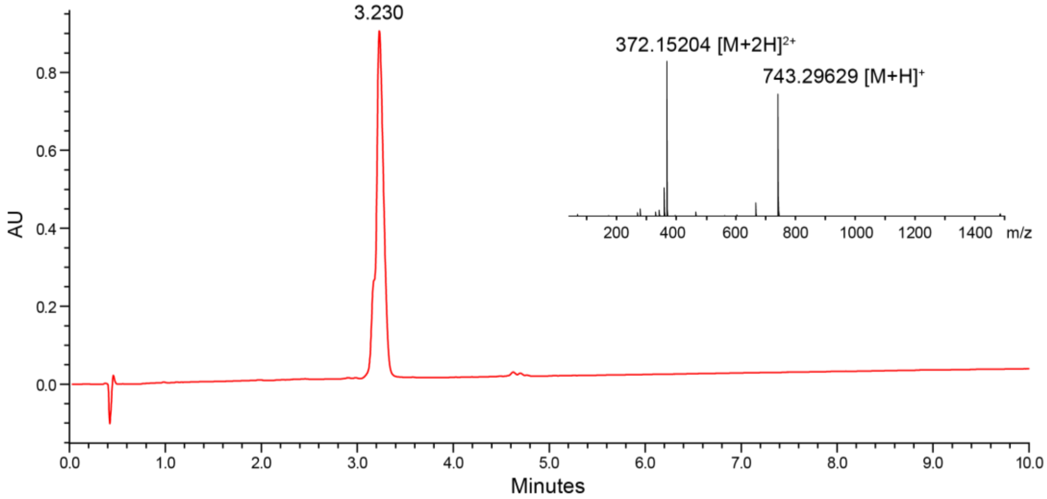

Figure S1: HPLC chromatogram and ESI mass spectrum (inset) of m[7]SOD

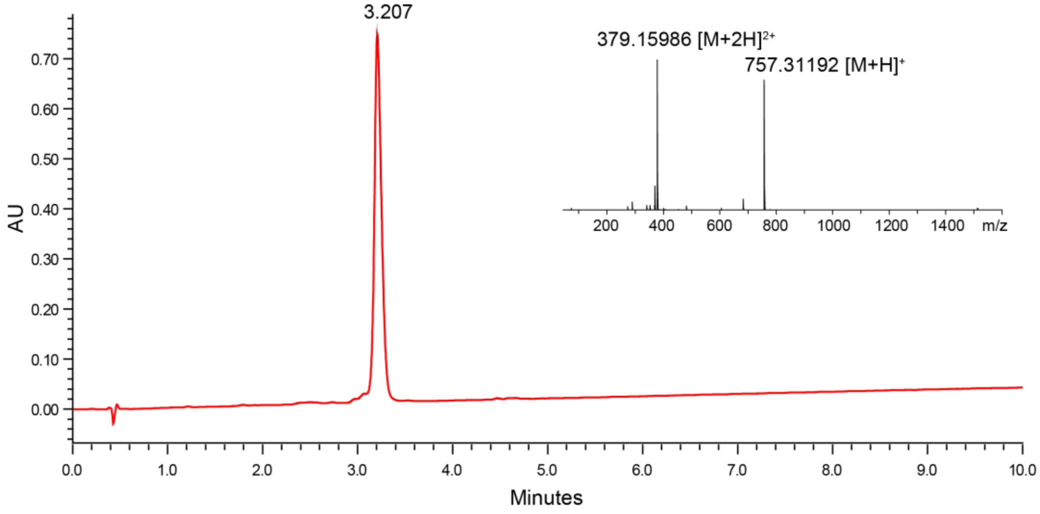

Figure S2: HPLC chromatogram and ESI mass spectrum (inset) of m^{7}SOD H1H'

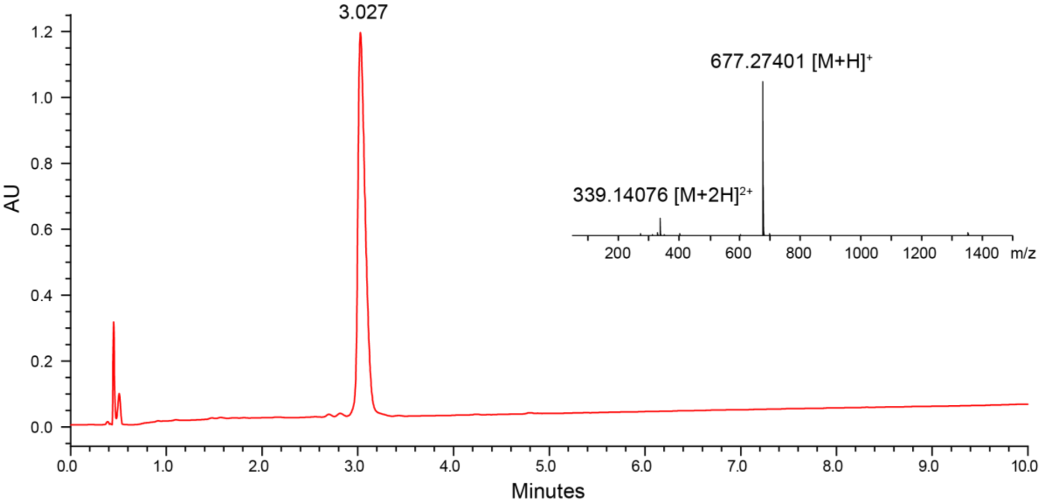

Figure S3: HPLC chromatogram and ESI mass spectrum (inset) of m^{7}SOD H1A

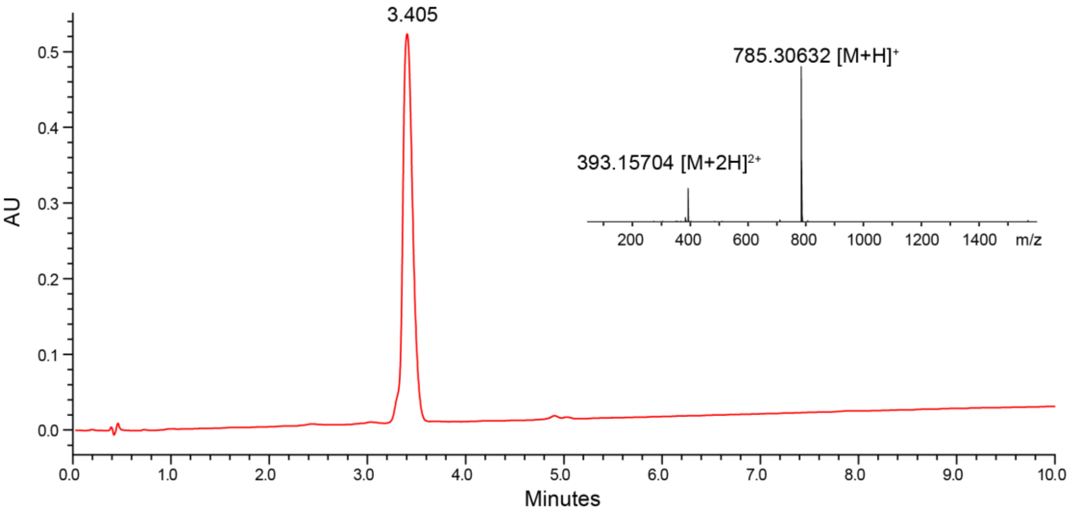

Figure S4: HPLC chromatogram and ESI mass spectrum (inset) of m^{7}SOD AcHis

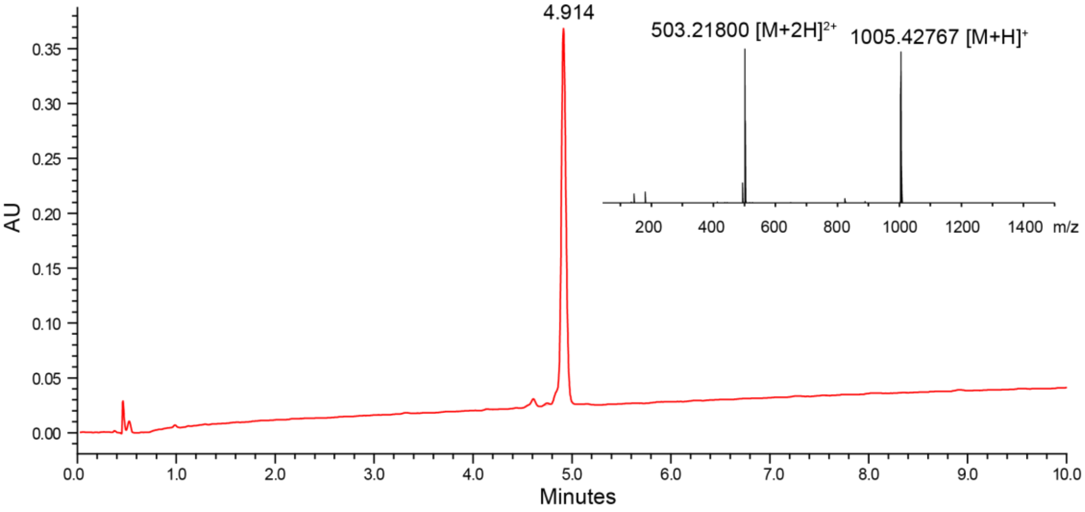

Figure S5: HPLC chromatogram and ESI mass spectrum (inset) of m^{9}SOD

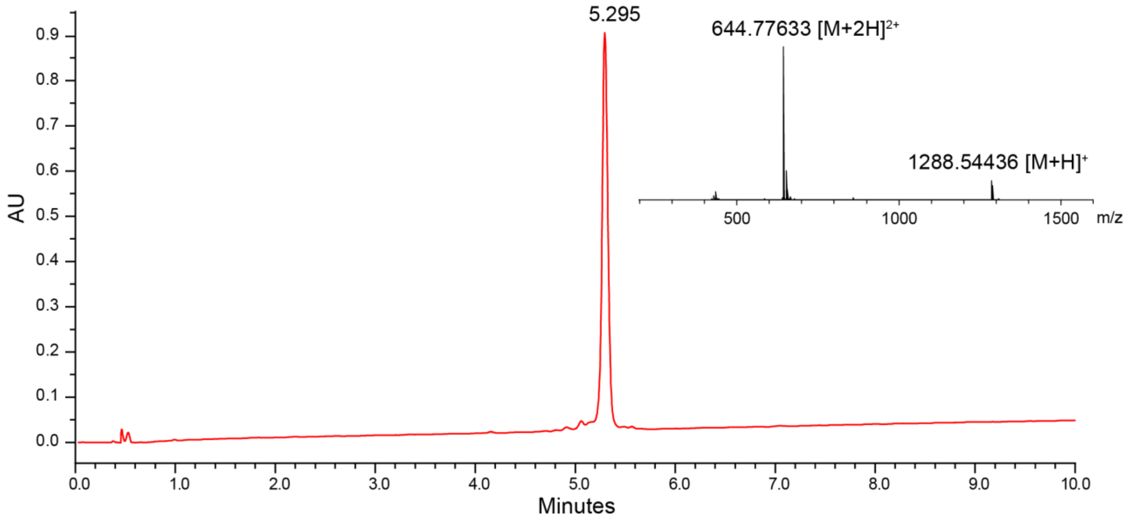

Figure S6: HPLC chromatogram and ESI mass spectrum (inset) of m[12]SOD

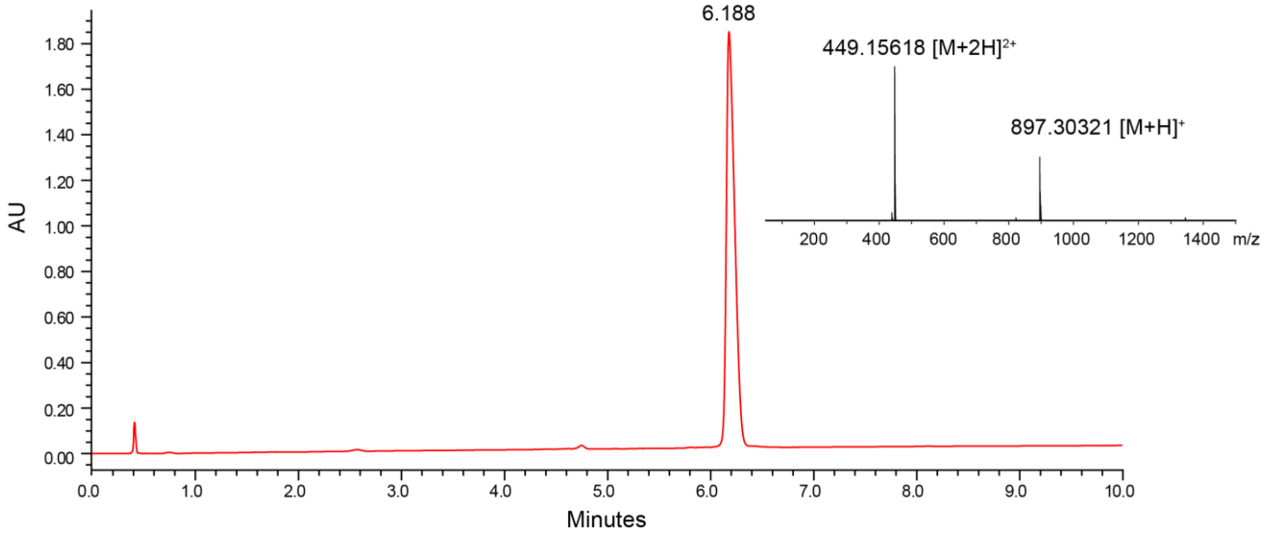

Figure S7: HPLC chromatogram and ESI mass spectrum (inset) of m[7]SOD TosHis

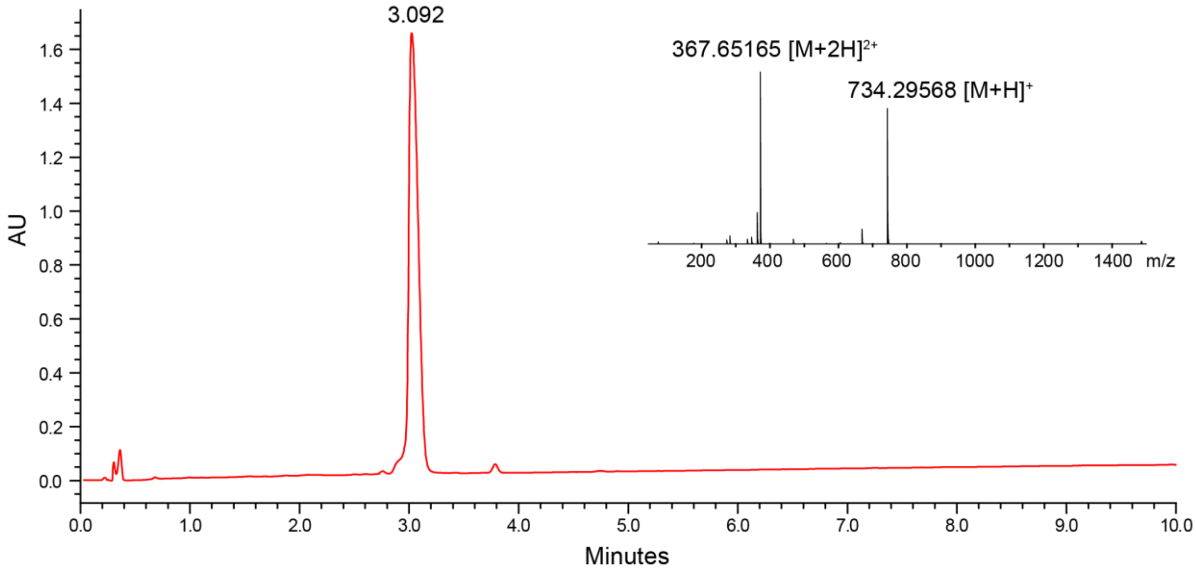

Figure S8: HPLC chromatogram and ESI mass spectrum (inset) of m^{7}SOD H1Q

UV-Vis spectra of NiSOD model peptides

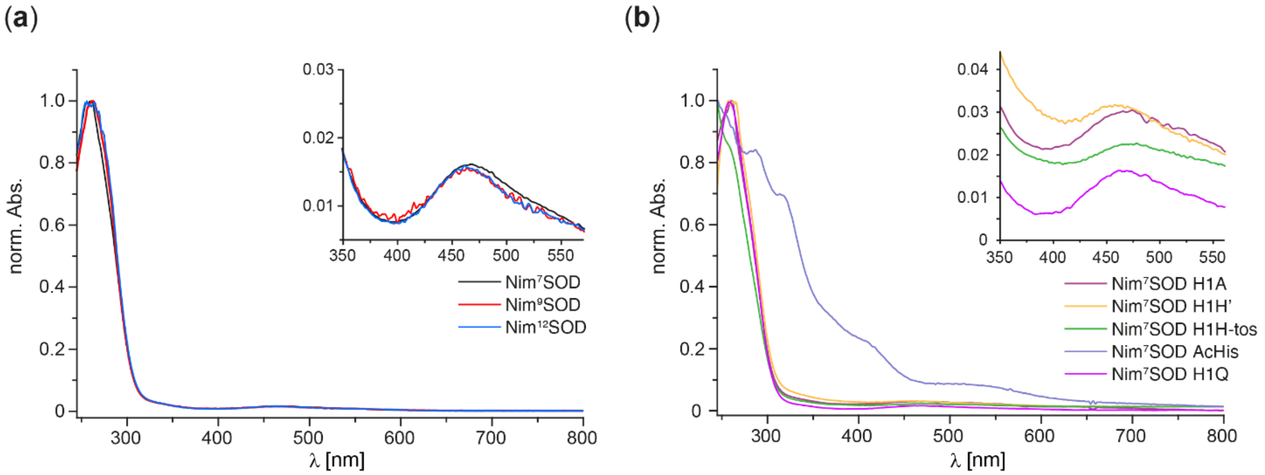

Figure S9: normalized UV-Vis spectra of the NiSOD mimics a) **1** (black trace), **7** (red trace) and **8** (light blue trace) and b) of **4** (blue trace), **3** (purple trace), **2** (orange trace), **5** (magenta trace) and **6** (green trace) in buffer (150 mM phosphate buffer, pH 8, 25°C). The inset shows the characteristic sulfur-to-Ni(II) ligand field transition at 460 nm.

2. Determination of the stability of the Ni-peptides

Peptide oxidation in air shown for Nim⁹SOD

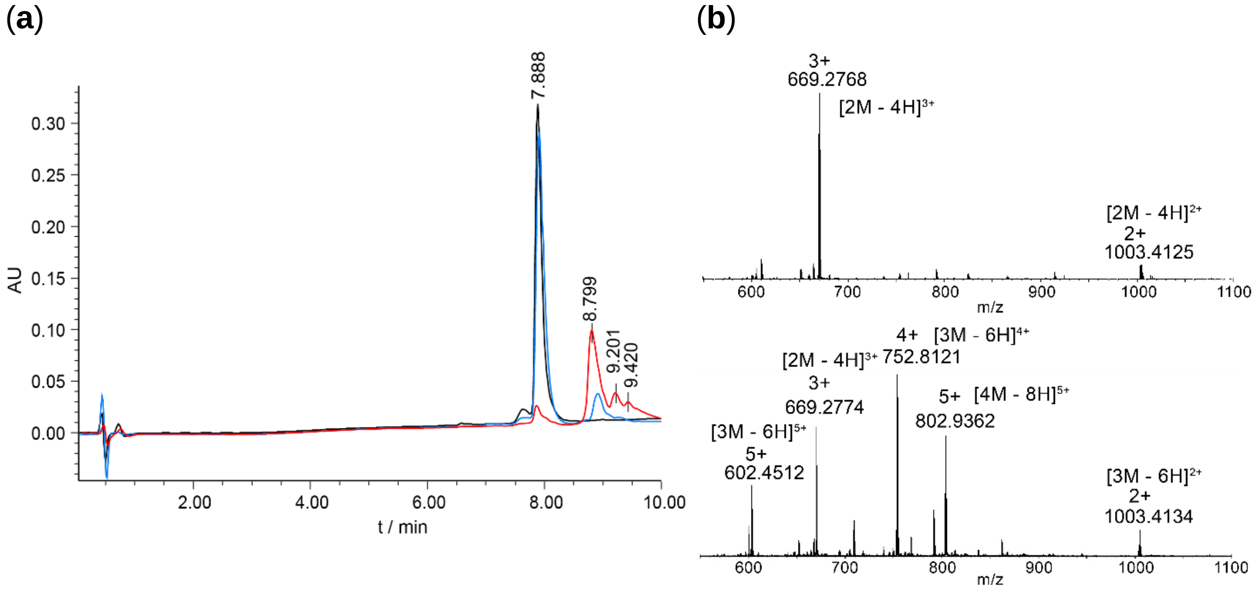

Figure S10: Oxidation process mediated by air exemplarily shown for Nim⁹SOD. a) overlay of HPLC chromatograms of Nim⁹SOD under various conditions. HPLC samples were prepared from 20 µL of the respective peptide solution in 80 µL water (0.1% TFA). black trace – m⁹SOD in water pH 3 (not degassed), blue trace – Nim⁹SOD in buffer (250 mM, phosphate, pH 7.8). The chromatogram was recorded immediately after Ni addition. red trace – sample from blue trace was aged for 24 hours on air. b) ESI mass spectra recorded from the peaks ranging from 8.799 to 9.420 min from the aged Nim⁹SOD sample (red trace). Peaks were identified as dimeric (m/z = 669.2768 {calc. m.i. 669.2774} $[2M-4H]^{3+}$; 1003.4125 {calc. m.i. 1003.4124} $[2M-4H]^{2+}$), trimeric (m/z = 602.4512 {calc. m.i. 602.4504} $[3M-6H]^{5+}$; 752.8121

{calc. m.i. 752.8111} $[3M\text{-}6H]^{4+}$; 1003.4134 {calc. m.i. 1003.4124} $[3M\text{-}6H]^{2+}$) and tetrameric (m/z = 802.9362 {calc. m.i. 802.9314} $[4M\text{-}8H]^{4+}$) m^9SOD. Please note, that the acidic conditions of the HPLC eluent system remove the Ni-ion from the peptide, thus the Ni-peptide complex is not observable under these conditions.

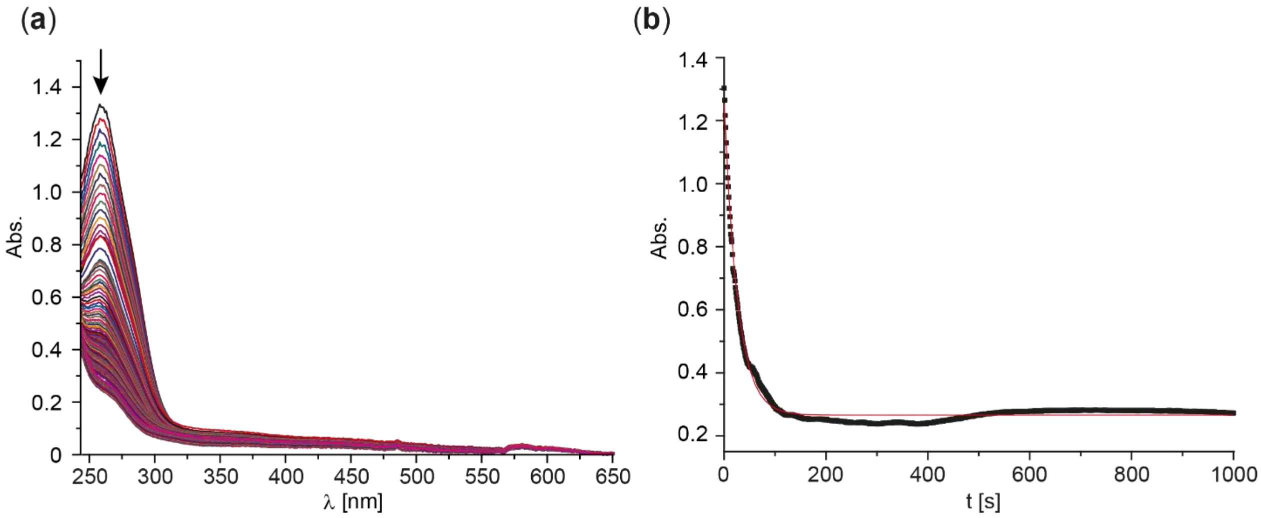

Figure S11: KO_2 (5 mM) induced decomposition of Nim^7SOD H1H' (**2**) (158 µM, 150 mM phosphate buffer, pH 8, 25°C) a) monitored via UV-Vis/stopped flow experiments (for clarity, spectra are shown at a 1 s interval for 1 to 100 s and at a 10 s interval from 101 to 620 s). b) time trace (black circles) of Ni-peptide decomposition extracted at 262 nm. The data were fitted via a 1st order decay (red line).

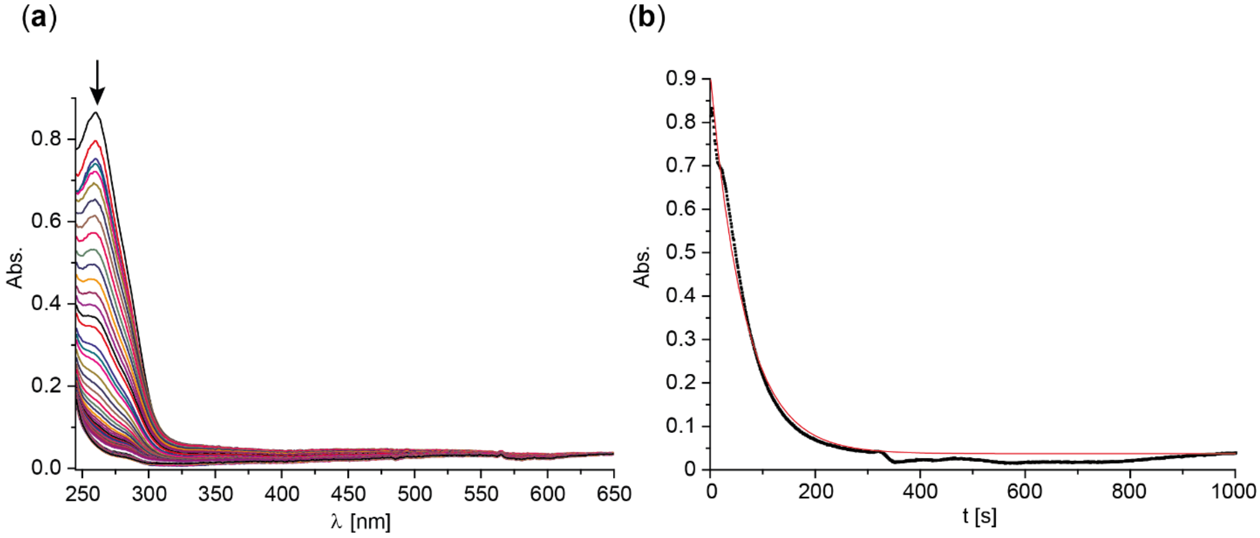

Figure S12: KO_2 (5 mM) induced decomposition of Nim^7SOD H1A (**3**) (40 µM, 150 mM phosphate buffer, pH 8, 25°C) a) monitored via UV-Vis/stopped flow experiments (for clarity, spectra are shown at a 5 s interval for 1 to 100 s and at a 10 s interval from 101 to 620 s). b) time trace (black circles) of Ni-peptide decomposition extracted at 262 nm. The data were fitted via a 1st order decay (red line).

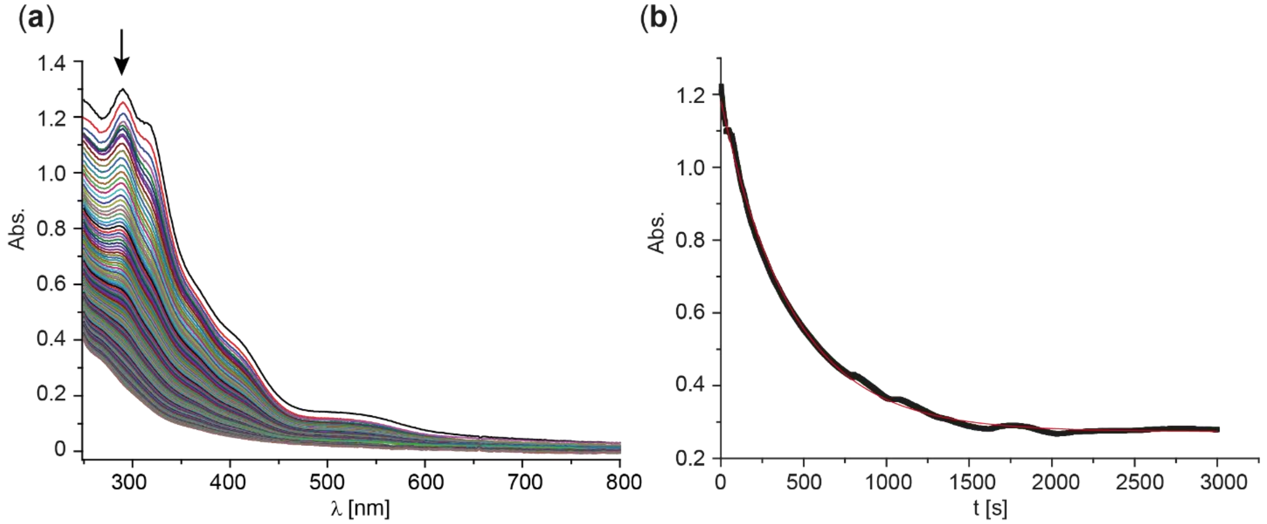

Figure S13: KO_2 (5 mM) induced decomposition of Nim[7]SOD AcHis (**4**) (158 µM, 150 mM phosphate buffer, pH 8, 25°C) a) monitored via UV-Vis/stopped flow experiments (for clarity, spectra are shown at a 10 s interval for 1 to 1800 s). b) time trace (black circles) of Ni-peptide decomposition extracted at 280 nm. The data were fitted via a 1st order decay (red line).

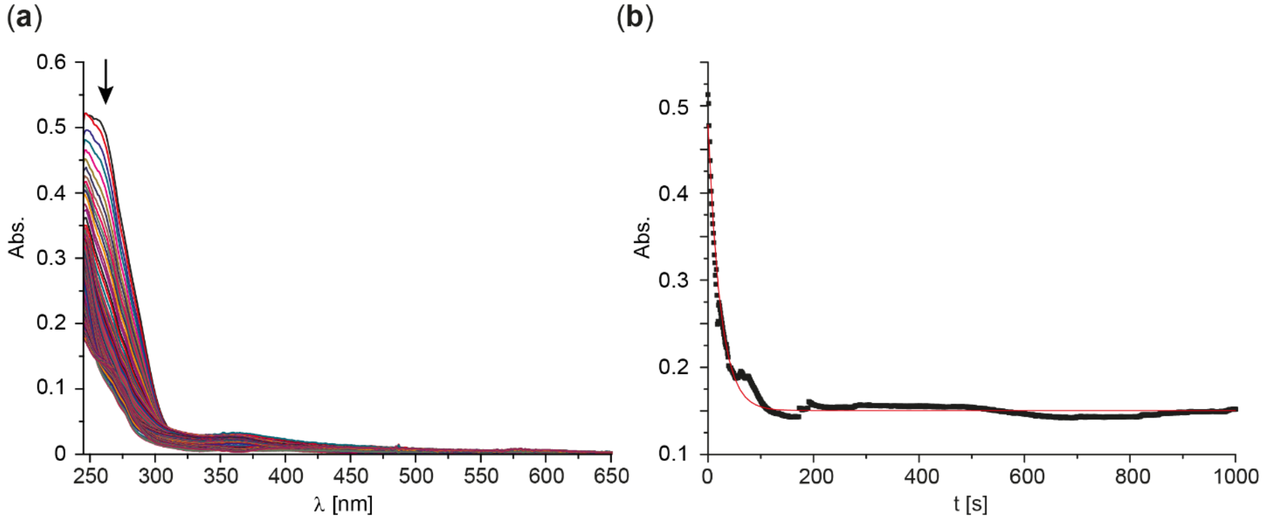

Figure S14: KO_2 (5 mM) induced decomposition of Nim[7]SOD H1H-tos (**6**) (40 µM, 150 mM phosphate buffer, pH 8, 25°C) a) monitored via UV-Vis/stopped flow experiments (for clarity, spectra are shown at a 1 s interval for 1 to 100 s and at a 10 s interval from 101 to 620 s). b) time trace (black circles) of Ni-peptide decomposition extracted at 255 nm. The data were fitted via a 1st order decay (red line).

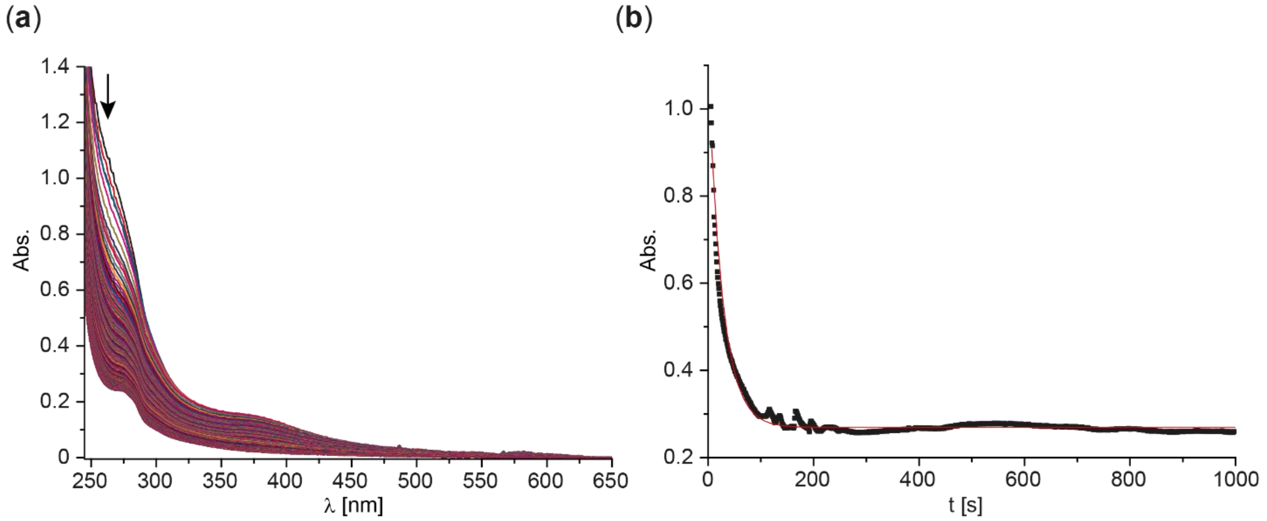

Figure S15: KO_2 (5 mM) induced decomposition of Nim^{9}SOD (**7**) (40 µM, 150 mM phosphate buffer, pH 8, 25°C) a) monitored via UV-Vis/stopped flow experiments (for clarity, spectra are shown at a 1 s interval for 6 to 1000 s. b) time trace (black circles) of Ni-peptide decomposition extracted at 262 nm. The data were fitted via a 1st order decay (red line).

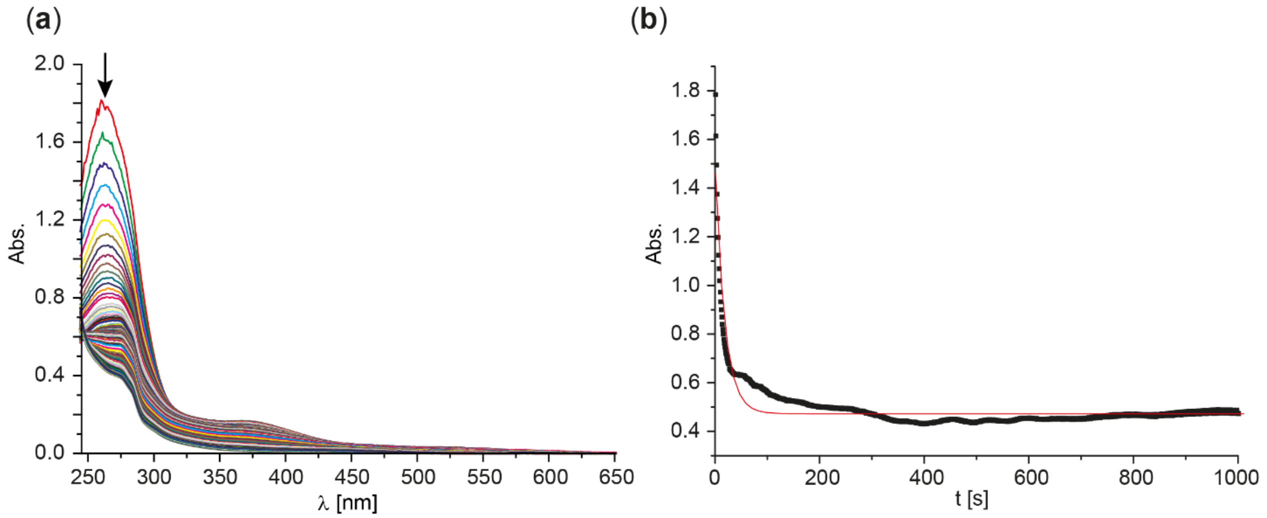

Figure S16: KO_2 (5 mM) induced decomposition of Nim^{12}SOD (**8**) (0.158 mM, 150 mM phosphate buffer, pH 8, 25°C) a) monitored via UV-Vis/stopped flow experiments (for clarity, spectra are shown at a 11 s interval for 1 to 100 s and at a 10 s interval from 101 to 620 s). b) time trace (black circles) of Ni-peptide decomposition extracted at 262 nm. The data were fitted via a 1st order decay (red line).

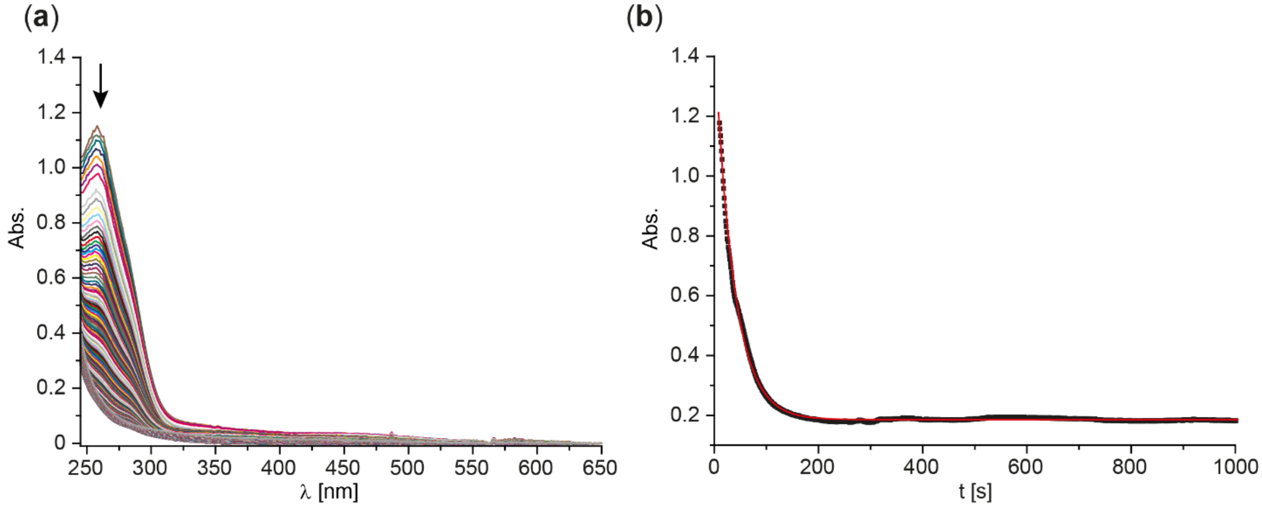

Figure S17: KO_2 (5 mM) induced decomposition of Nim[7]SOD H1Q (**5**) (0.158 mM, 150 mM phosphate buffer, pH 8, 25°C) a) monitored via UV-Vis/stopped flow experiments (for clarity, spectra are shown at a 1 s interval for 10 to 620 s). b) time trace (black circles) of Ni-peptide decomposition extracted at 262 nm. The data were fitted via a 1st order decay (red line).

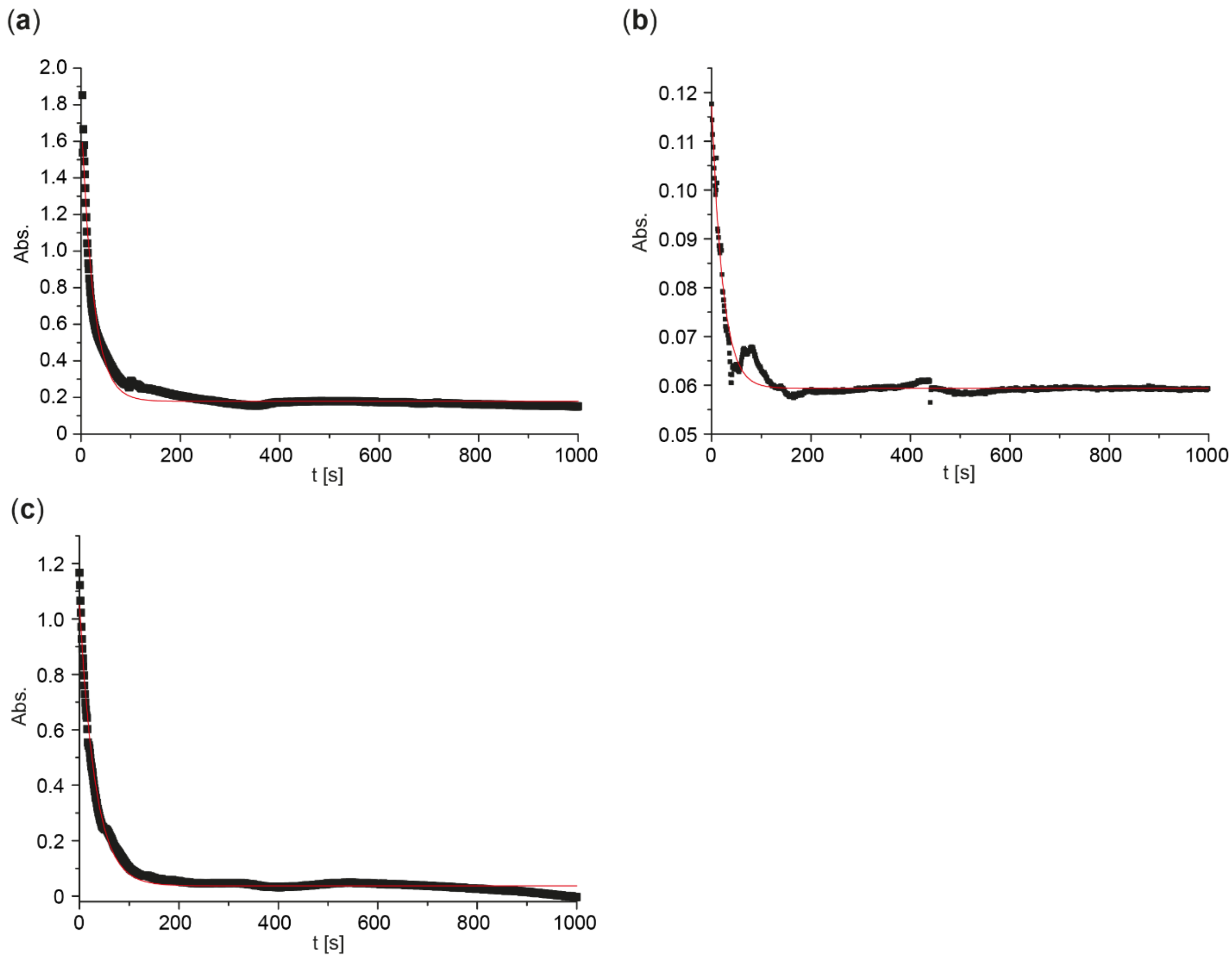

Figure S18: Time traces of KO_2 (5 mM) induced decomposition of Nim^{7}SOD (**1**) in 150 mM phosphate buffer (pH 8, 25°C) monitored at a) 262 nm (0.158 mM peptide concentration) b) 460 nm (0.158 mM peptide concentration) and c) 262 nm (40 µM peptide concentration). The data were fitted via a 1st order decay (red line).

Peptide treatment with KO_2

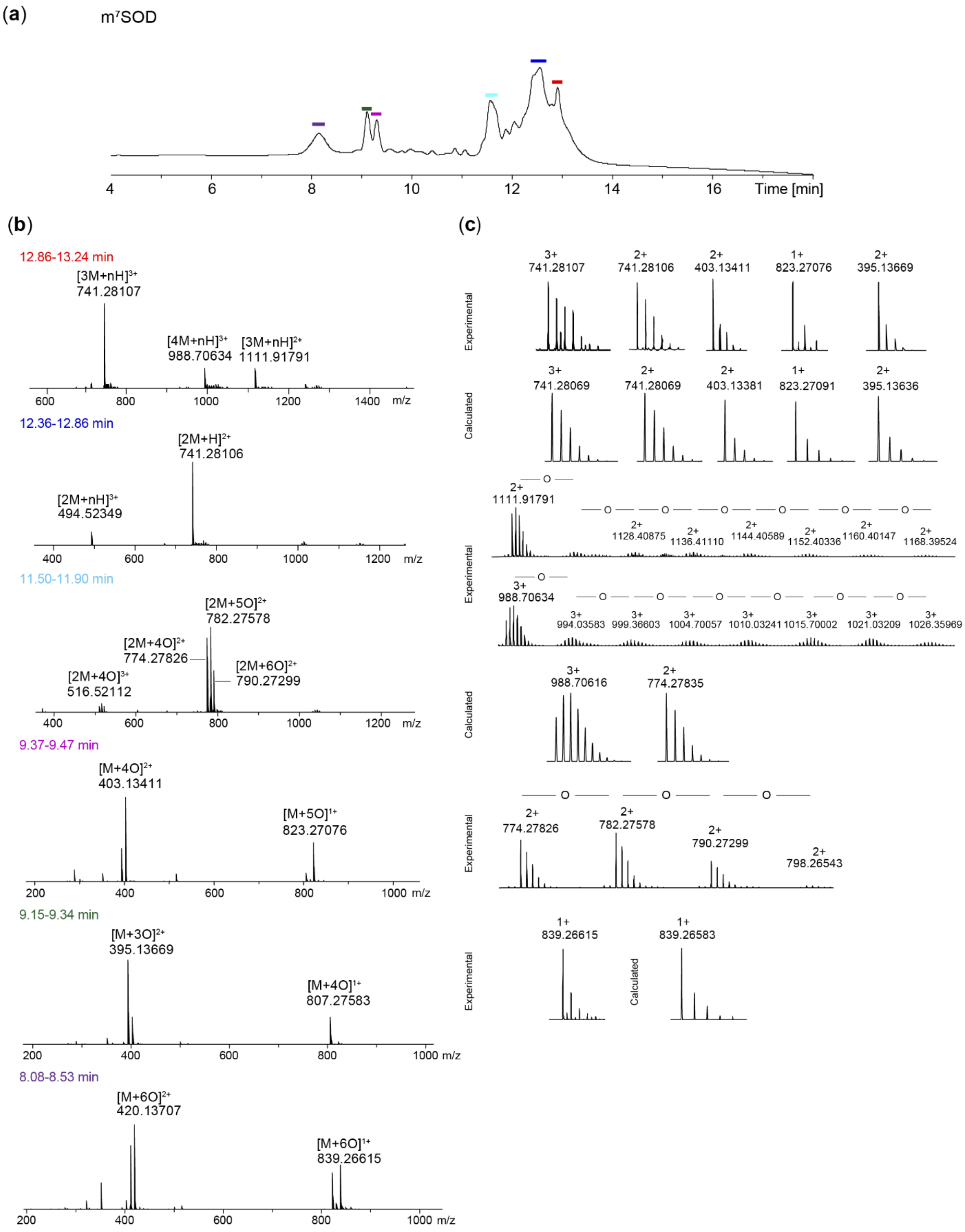

Figure S19: LC-MS analysis of Nim7SOD (1 mM, phosphate buffer 150 mM, pH 8.0) treated with KO_2. a) resulting HPLC chromatogram of the peptide after treatment with KO_2 b) Mass spectra for the peptide peaks indicated (colored lines) in a). c) experimental and simulated isotope patterns of the peptide species, which

were identified in the KO_2/peptide solution. [M] corresponds to the mass of the linear peptide minus two protons.

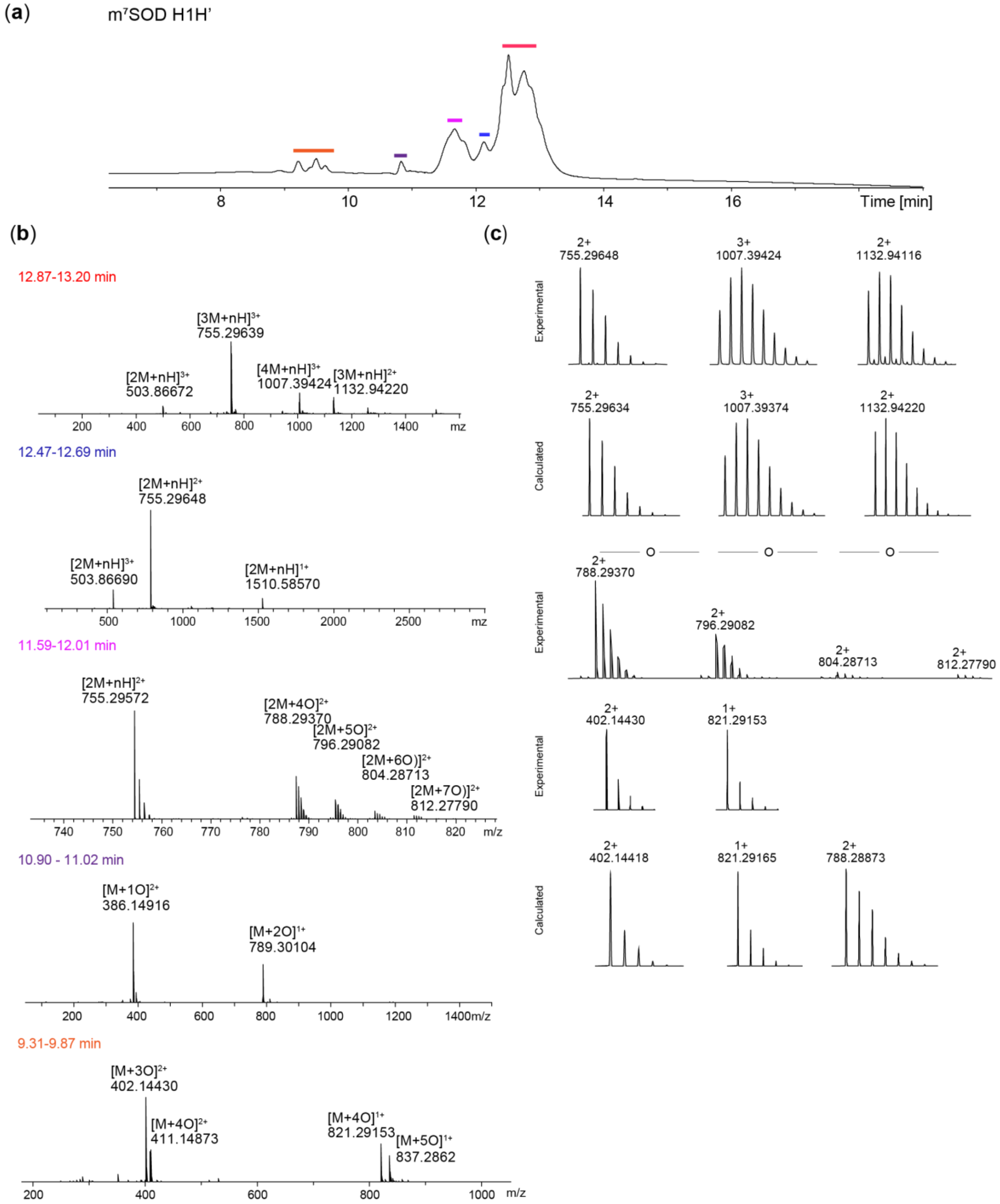

Figure S20: LC-MS analysis of Nim7SOD H1H' (1 mM, phosphate buffer 150 mM, pH 8.0) treated with KO_2. a) resulting HPLC chromatogram of the peptide after treatment with KO_2 b) Mass spectra for the peptide peaks indicated (colored lines) in a). c) experimental and simulated isotope patterns of the peptide species, which were identified in the KO_2/peptide solution. [M] corresponds to the mass of the linear peptide minus two protons.

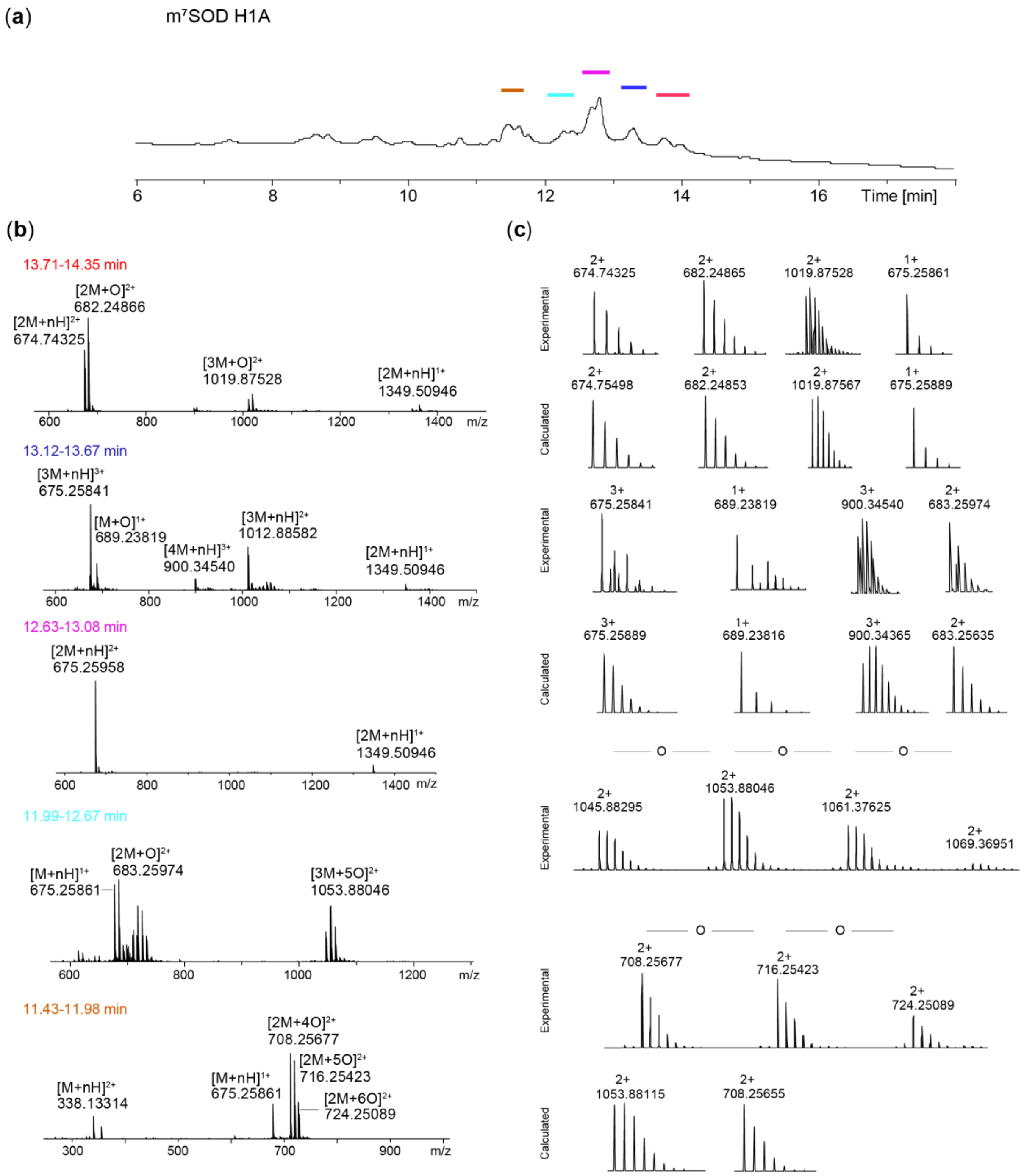

Figure S21: LC-MS analysis of Nim^{7}SOD H1A (1 mM, phosphate buffer 150 mM, pH 8.0) treated with KO_2. a) resulting HPLC chromatogram of the peptide after treatment with KO_2 b) Mass spectra for the peptide peaks indicated (colored lines) in a). c) experimental and simulated isotope patterns of the peptide species, which were identified in the KO_2/peptide solution. [M] corresponds to the mass of the linear peptide minus two protons.

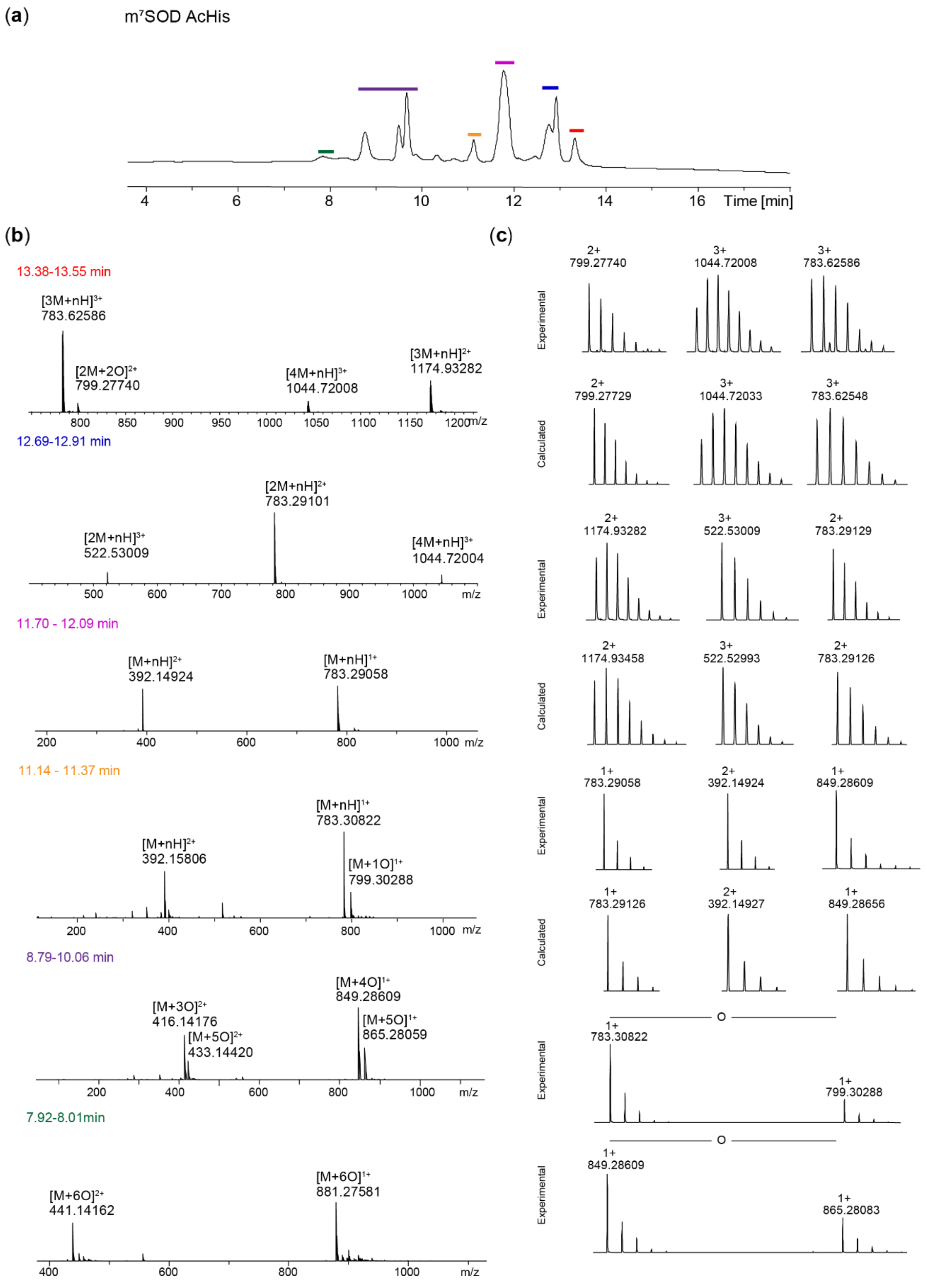

Figure S22: LC-MS analysis of Nim7SOD AcHis (1 mM, phosphate buffer 150 mM, pH 8.0) treated with KO_2. a) resulting HPLC chromatogram of the peptide after treatment with KO_2 b) Mass spectra for the peptide peaks indicated (colored lines) in a). c) experimental and simulated isotope patterns of the peptide species, which were identified in the KO_2/peptide solution. [M] corresponds to the mass of the linear peptide minus two protons.

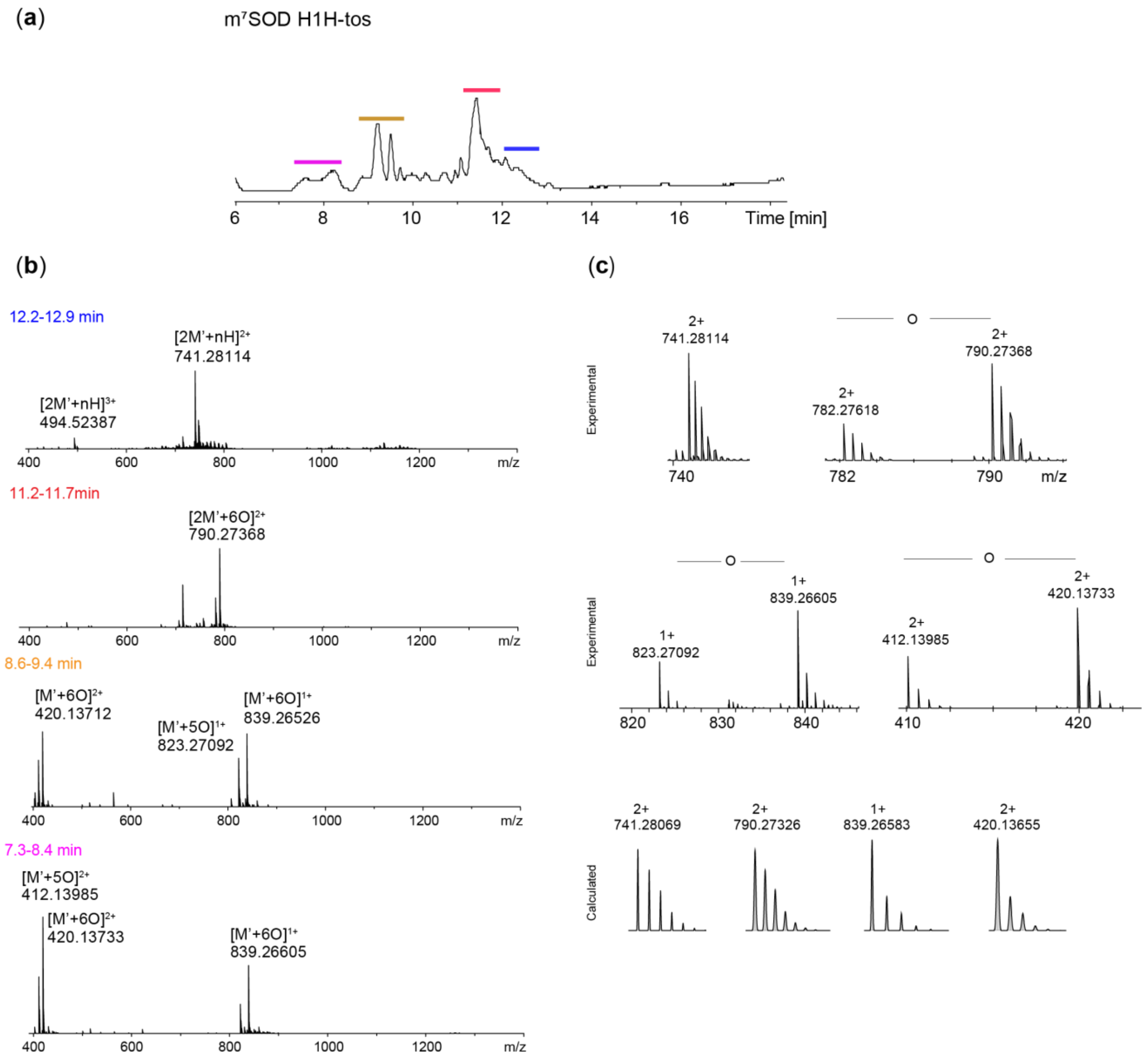

Figure S23: LC-MS analysis of Nim7SOD H1H-tos (1 mM, phosphate buffer 150 mM, pH 8.0) treated with KO_2. a) resulting HPLC chromatogram of the peptide after treatment with KO_2 b) Mass spectra for the peptide peaks indicated (colored lines) in a). c) experimental and simulated isotope patterns of the peptide species, which were identified in the KO_2/peptide solution. [M] corresponds to the mass of the linear peptide minus two protons.

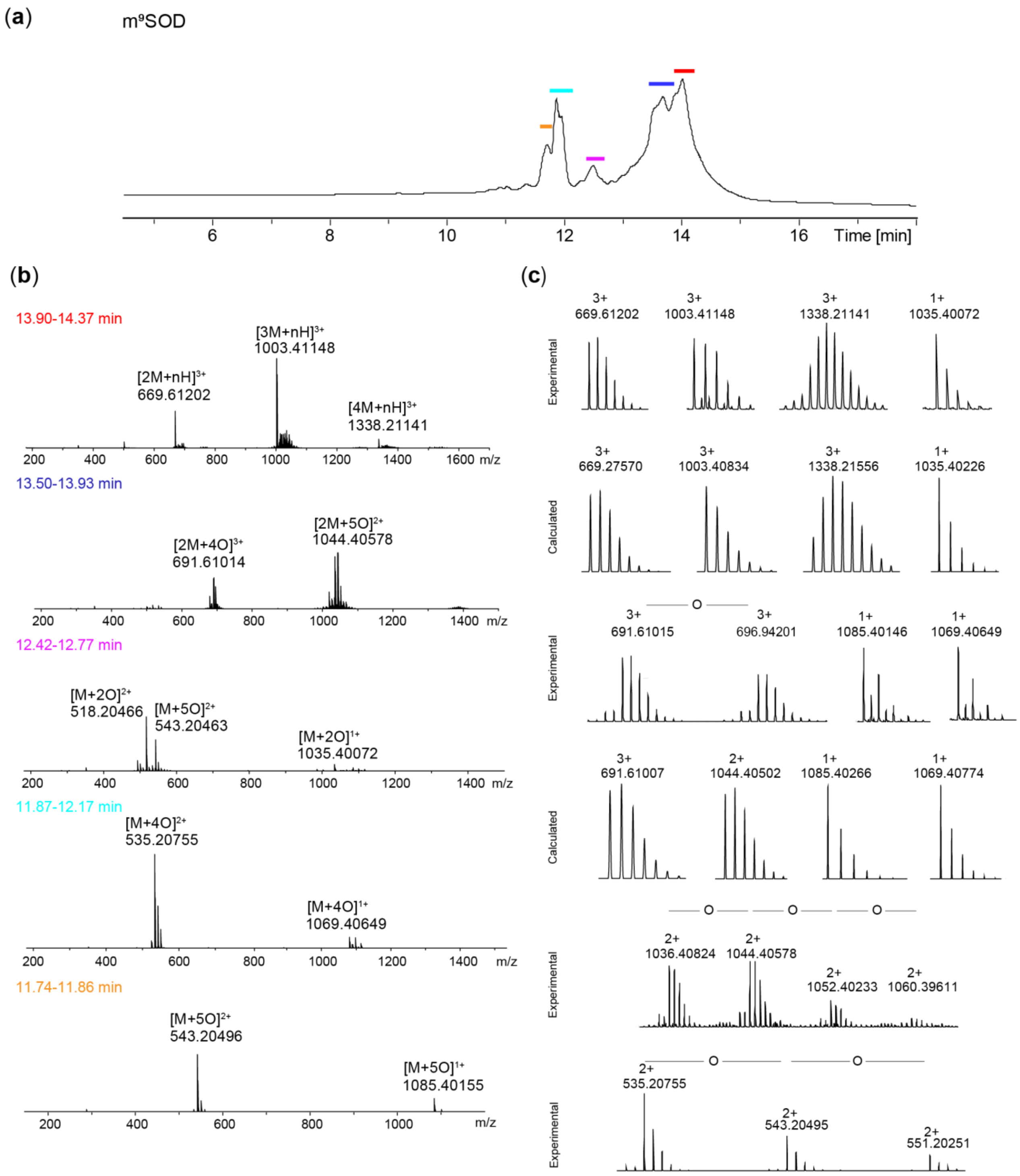

Figure S24: LC-MS analysis of Nim9SOD (1 mM, phosphate buffer 150 mM, pH 8.0) treated with KO_2. a) resulting HPLC chromatogram of the peptide after treatment with KO_2 b) Mass spectra for the peptide peaks indicated (colored lines) in a). c) experimental and simulated isotope patterns of the peptide species, which were identified in the KO_2/peptide solution. [M] corresponds to the mass of the linear peptide minus two protons.

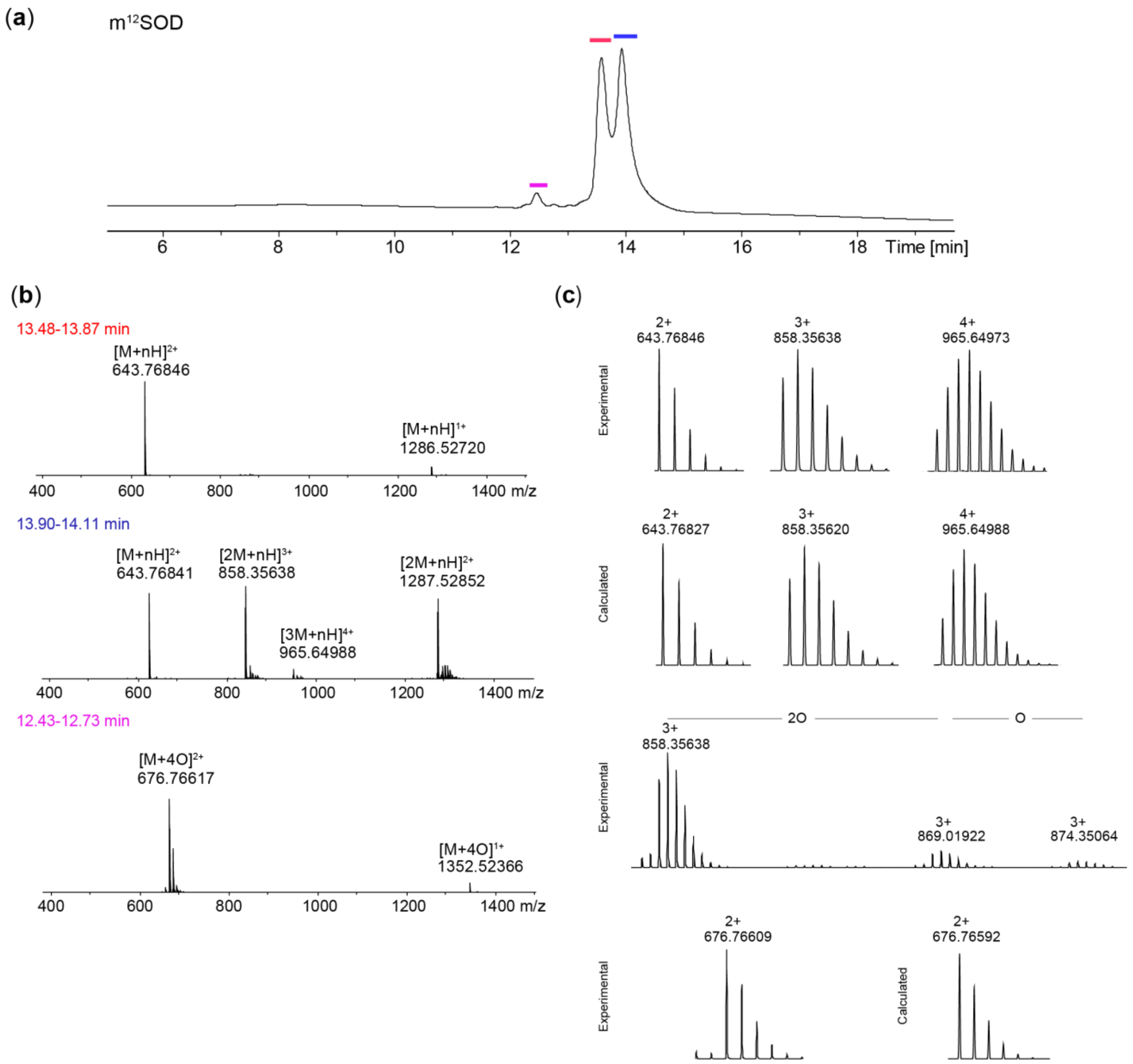

Figure S25: LC-MS analysis of Nim12SOD (1 mM, phosphate buffer 150 mM, pH 8.0) treated with KO_2. a) resulting HPLC chromatogram of the peptide after treatment with KO_2 b) Mass spectra for the peptide peaks indicated (colored lines) in a). c) experimental and simulated isotope patterns of the peptide species, which were identified in the KO_2/peptide solution. [M] corresponds to the mass of the linear peptide minus two protons.

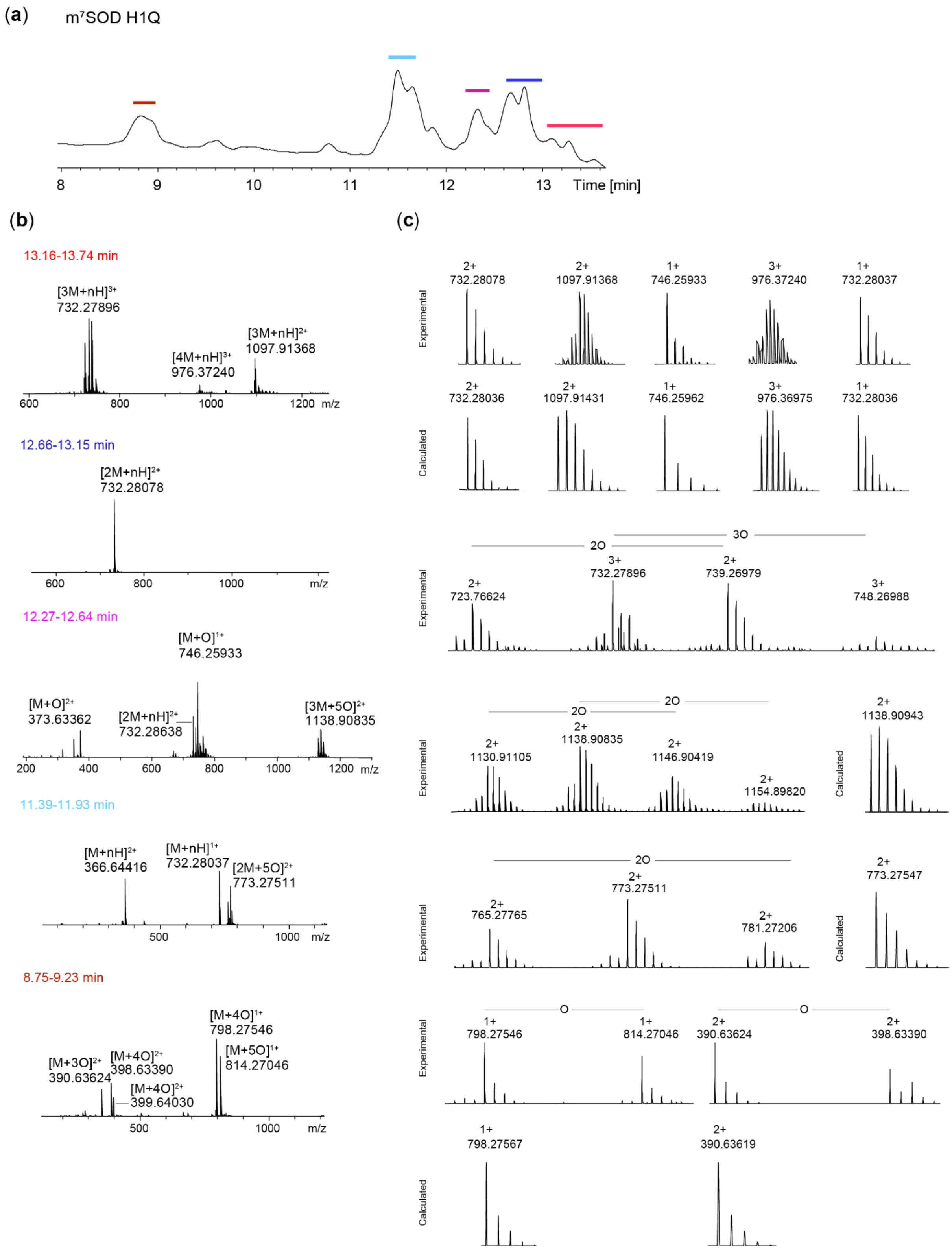

Figure S26: LC-MS analysis of Nim7SOD H1Q (1 mM, phosphate buffer 150 mM, pH 8.0) treated with KO_2. a) resulting HPLC chromatogram of the peptide after treatment with KO_2 b) Mass spectra for the peptide peaks indicated (colored lines) in a). c) experimental and simulated isotope patterns of the peptide species, which were identified in the KO_2/peptide solution. [M] corresponds to the mass of the linear peptide minus two protons.

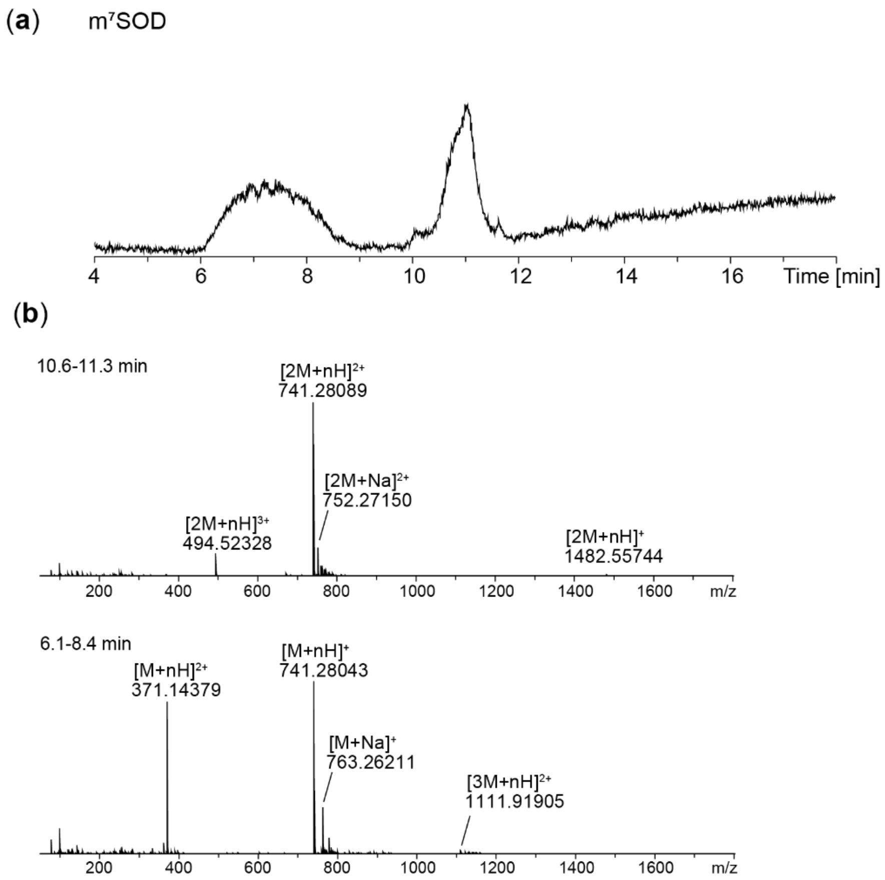

Figure S27: LC-MS analysis of m^{7}SOD (1 mM, phosphate buffer 150 mM, pH 8.0) without Ni(II) treated with KO_2. a) resulting HPLC chromatogram of the peptide after treatment with KO_2 b) Mass spectra for the peptide peaks

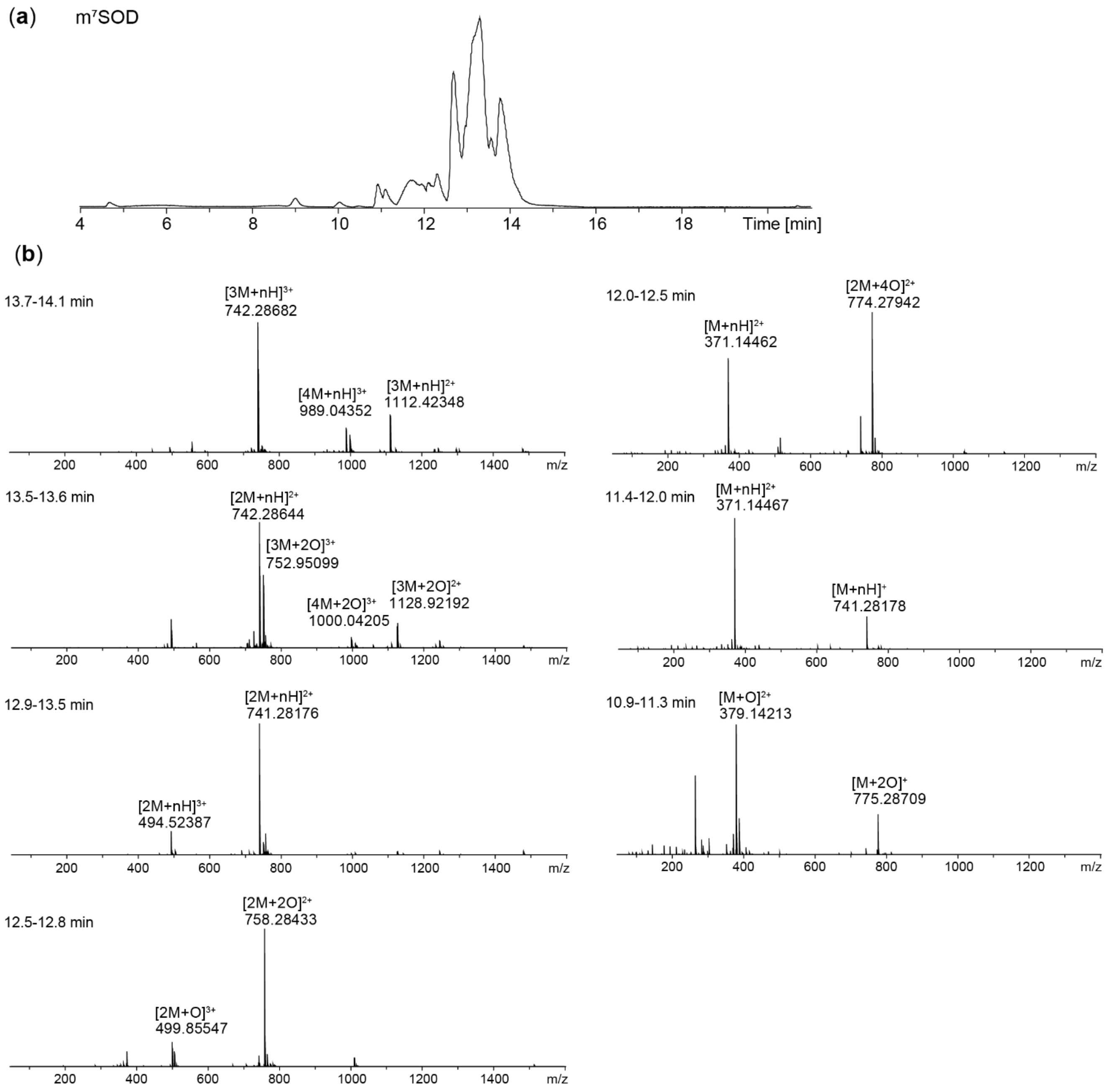

Figure S28: LC-MS analysis of Nim7SOD (1 mM, phosphate buffer 150 mM, pH 8.0) treated with KO_2 and ascorbic acid. a) resulting HPLC chromatogram of the peptide after treatment with KO_2 and ascorbic acid b) Mass spectra for the peptide peaks

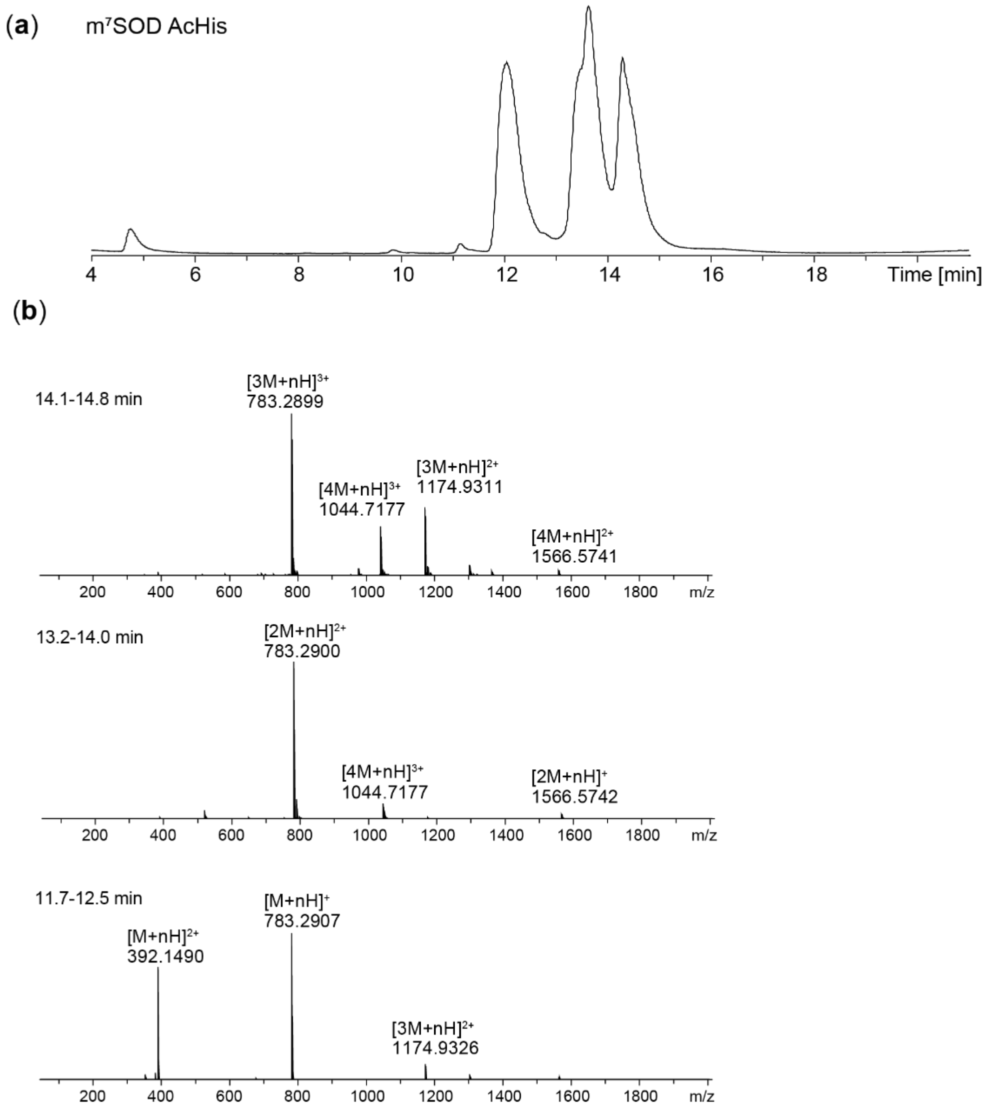

Figure S29: LC-MS analysis of Nim^{7}SOD AcHis (1 mM, phosphate buffer 150 mM, pH 8.0) treated with KO_2 and ascorbic acid. a) resulting HPLC chromatogram of the peptide after treatment with KO_2 and ascorbic acid b) Mass spectra for the peptide peaks

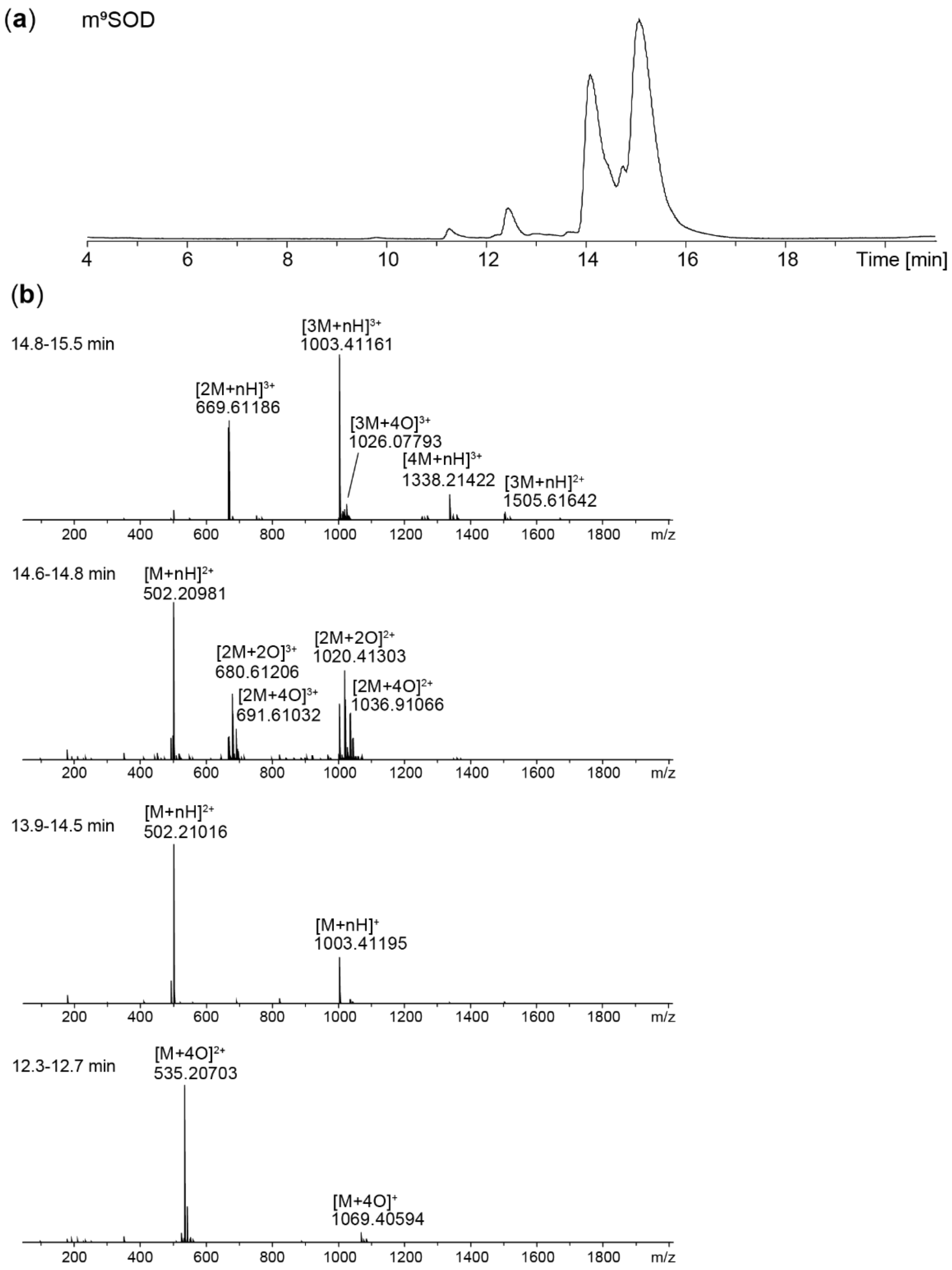

Figure S30: LC-MS analysis of Nim[9]SOD (1 mM, phosphate buffer 150 mM, pH 8.0) treated with KO_2 and ascorbic acid. a) resulting HPLC chromatogram of the peptide after treatment with KO_2 and ascorbic acid b) Mass spectra for the peptide peaks

3. Determination of the catalytic activity of the Ni-peptides

Stopped-Flow experiments

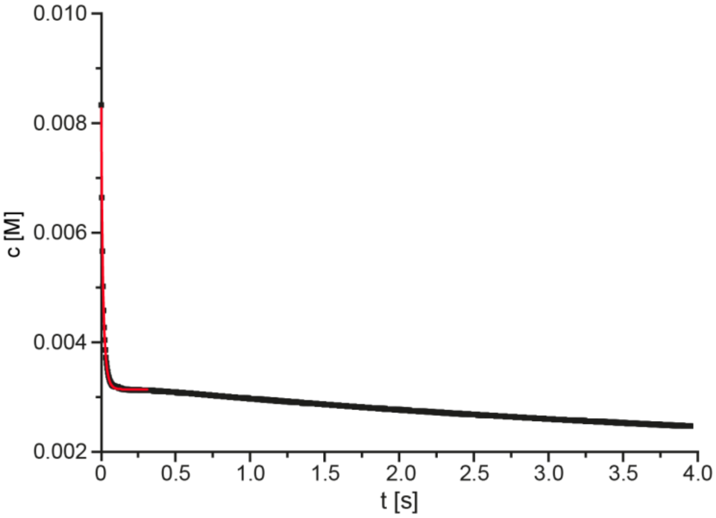

Figure S31: Superoxide decay observed at 250 nm catalysed by Nim[7]SOD H1H'. Superoxide (50 mM, DMSO) was mixed with Nim[7]SOD H1H' (0.033 mM, 150 mM phosphate buffer, pH 8, 25.0°C) in a 1:10 (v:v) ratio. Data points (black squares) were fitted according to eq. 3 (red trace).

Table S1: Summary of the observed 1st order superoxide decay rates k_{obs} (value indicated with an # was identified as an outlier) and the catalytic background activity k_1. K_1 values were determined independently from the uncatalyzed superoxide decay experiments, which were performed prior to the measurements of each model peptide.

	k_1 [s^{-1}]	k_{obs} [s^{-1}]	k_{obs} [s^{-1}]	k_{obs} [s^{-1}]	k_{obs} [s^{-1}]	k_{obs} [s^{-1}]	k_{cat} x 10^6 [M^{-1} s^{-1}]	Linearity R^2
		0.9 µM	*13.5 µM*	*21.6 µM*	*29.7 µM*	*36 µM*		
Nim^7SOD	(2.0 ± 0.5) x 10^{-3}	7.8 ± 0.6	31.0 ± 1.9	52.5 ± 3.6	56.4 ± 3.7	61.2 ± 4.1	1.7 ± 0.3	0.97
Nim^7SOD H1H‘	(1.0 ± 0.5) x 10^{-3}	5.5 ± 0.71	26.4 ± 1.5	52.6 ± 3.2	65.6 ± 3.8	64.1 ± 4.1#	2.8 ± 0.4	0.94
Nim^7SOD AcHis	10.1 ± 0.1	24.6 ± 1.1	22.8 ± 1.2	7.2 ± 1.1	6.0 ± 1.1	2.5 ± 1.1	n.d.	
$Nim^{12}SOD$	0.16 ± 0.1	22.2 ± 1.3	49.6 ± 2.8	75.5 ± 4.4	92.0 ± 5.7	122.7 ± 9.6	2.5 ± 0.4	0.98
		4.5 µM	*13.5 µM*	*21.6 µM*	*29.7 µM*	*36 µM*		
Nim^9SOD	2.2 ± 0.2	13.5 ± 0.8	46.3 ± 2.4	88.5 ± 5.1	109.1 ± 6.0	131.8 ± 8.9	3.5 ± 0.6	0.99
Nim^7SOD H1H-tos	2.6 ± 0.3	37.2 ± 2.2	56.2 ± 2.8	74.6 ± 2.1	92.2 ± 5.4	106.0 ± 7.5	2.2 ± 0.4	0.99
Nim^7SOD H1A	2.4 ± 0.2	27.2 ± 1.5	39.6 ±2.1	51.0 ± 2.7	61.0 ± 3.4	70.2 ± 3.9	1.2 ± 0.3	0.99
Nim^7SOD H1Q	10.0 ± 0.5	23.7 ± 1.3	34.1 ±1.8	55.7 ± 2.9	67.2 ± 3.7	90.7 ± 5.1	1.9 ± 0.3	0.95
$NiCl_2$	3.4 ± 0.01	3.9 ± 0.02	1.9 ± 0.03	2.1 ± 0.03	0.6 ± 0.07	0.5 ± 0.09	n.d.	
		0.9 nM	*13.5 nM*	*21.6 nM*	*29.7 nM*	*36 nM*		
CuZnSOD	5.0 ± 0.3	8.9 ± 1.0	17.0 ± 1.7	23.4 ± 2.4	33.2 ± 3.4	38.5 ± 4.0	910 ± 30	0.98

4. Structure and Energy calculations

MD simulations

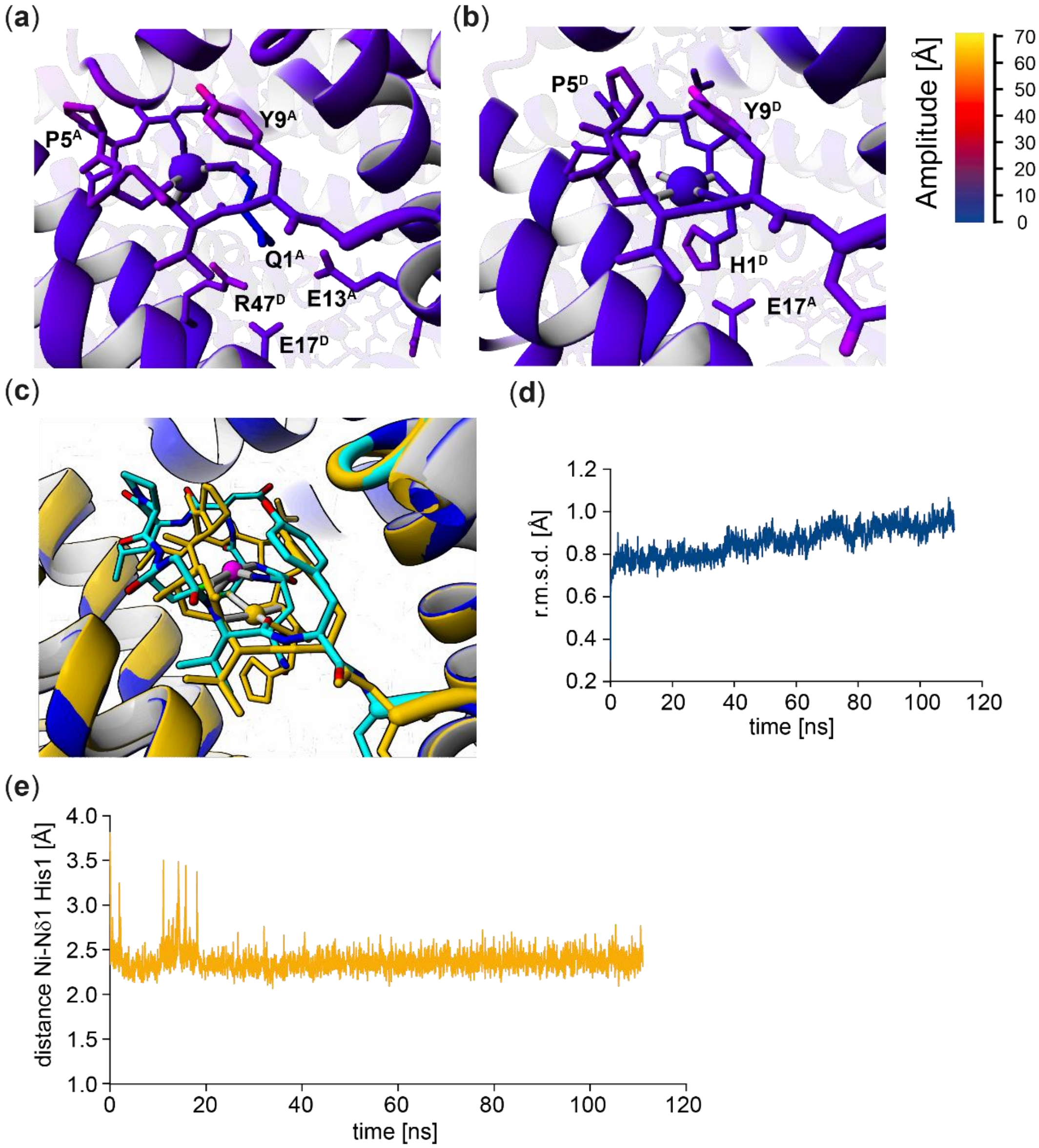

Figure S32: Color coded amplitude of flexibility at the NiSOD active site according to the crystallographic b-factor calculated from average root mean square fluctuations. Comparison of a) NiSOD H1Q and b) wt. NiSOD. c) superposition of the active site of wt. NiSOD (orange) and NiSOD H1Q (colored according to Figure 6) d) root mean square deviation (r.m.s.d.) of NiSOD with respect to the starting structure. e) structural mobility of the imidazole side chain of His1 with respect to the imidazole Nδ1 – Ni(II) distance as a function of simulation time.

First principles calculations (DFT)

Table S2: Description of the different peptide model systems for the DFT calculations of peptides **1** and **4**. The second column descripts the orientation of the imidazole of His1 towards (inside) the Ni-ion or away (outside) from the Ni-ion.

peptide model	His1 orientation	protonation Cys2 amide	protonation His1 amine/amide
**1_m1**	**outside**	NH_{Cys2}	
**1_m2**	**inside**	NH_{Cys2}	
**1_m3**	**outside**	N_{Cys2}	
**1_m4**	**inside**	N_{Cys2}	
**4_m1**	**outside**	NH_{Cys2}	NH_{His1}
**4_m2**	**inside**	NH_{Cys2}	NH_{His1}
**4_m3**	**outside**	N_{Cys2}	NH_{His1}
**4_m4**	**inside**	N_{Cys2}	NH_{His1}
**4_m5**	**outside**	N_{Cys2}	N_{His1}
**4_m6**	**inside**	N_{Cys2}	N_{His1}

Table S3: Relative energies of the different peptides calculated in vacuum (vac) and solvent (sol) water at BP86-D(MARIJ)/cc-pVTZ level of theory.

peptide model	rel. $energy_{vac}$ (kcal/mol)	rel. $energy_{sol}$ (kcal/mol)
**1_m1**	4.9	5.4
**1_m2**	0	0
**1_m3**	7.2	3.5
**1_m4**	0	0
**4_m1**	11.4	2.0
**4_m2**	0	0
**4_m3**	4.3	9.6
**4_m4**	0	0
**4_m5**	0.3	0.6
**4_m6**	0.0	0.0

Table S4: Bond length (Å) of Ni(II) to the coordinating ligands at BP86-D(MARIJ)/cc-pVTZ level of theory.

peptide model	NH2/N(H)C	NH/N	S(Cys2)	S(Cys6)	O(Leu4)	Nδ1(His1)
**1_m1**	1.993	1.922	2.186	2.232	3.085	
**1_m2**	2.035	1.897	2.187	2.245	2.972	3.790
**1_m3**	1.998	1.910	2.183	2.248	3.368	
**1_m4**	2.029	1.890	2.183	2.262	3.261	3.747
**4_m1**	2.107	1.911	2.185	2.197	2.787	
**4_m2**	2.112	1.904	2.191	2.231	2.965	3.654
**4_m3**	2.077	1.900	2.183	2.217	2.949	
**4_m4**	2.086	1.885	2.188	2.239	3.311	3.597
**4_m5**	2.074	1.916	2.207	2.216	2.989	
**4_m6**	2.109	1.898	2.209	2.241	3.222	3.721

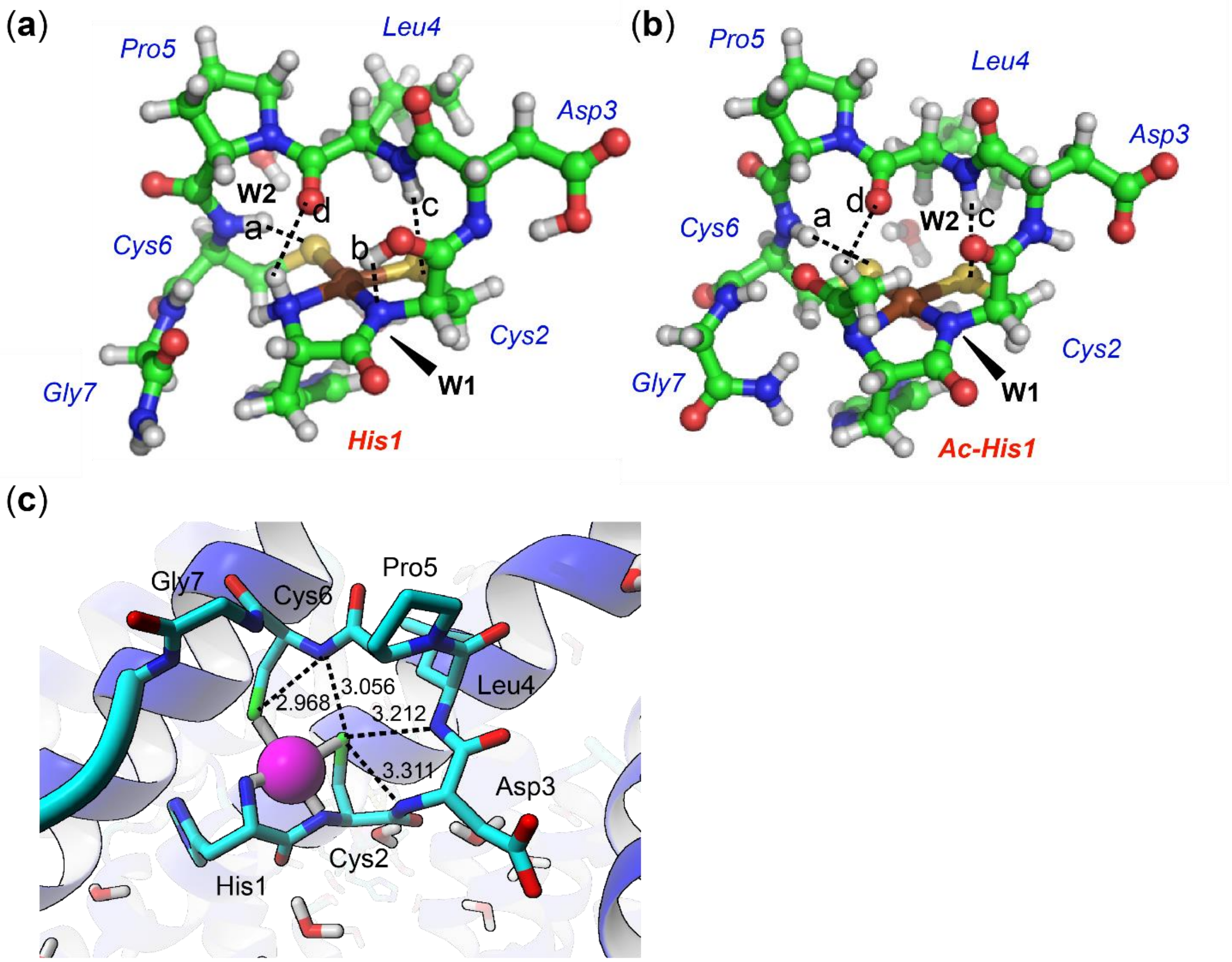

Figure S33: Structural optimized peptides with indicated hydrogen bonds (dashed lines) of a) **1** and b) **4** with His1 oriented towards Ni(II) and the amide group deprotonated at BP86-D(MARIJ)/cc-pVTZ level of theory (Color code: Ni: brown, C: green, S: yellow, O: red, N: blue). c) H-bond pattern and distances (Å) between the respective heavy atoms within the active site of the NiSOD enzyme indicating a similar H-bond network to 1 (H-bonds a and c).

Table S5: Hydrogen bonds (Å) of the coordinating ligands to the peptide or water molecules at BP86-D(MARIJ)/cc-pVTZ level of theory labelled according to Figure S33a,b.

peptide	a	b	c	d	S(Cys2)-W1	S(Cys6)-W1	S(Cys6)-W2
**1_m1**	1.995	2.083	2.459	2.256	2.431	2.392	2.261
**1_m2**	2.015	2.008	2.593	2.270	2.394	2.374	2.248
**1_m3**	2.241	-	2.573	2.332	2.514	2.278	2.259
**1_m4**	1.941	-	2.764	2.402	2.365	2.310	2.233
**4_m1**	2.396	1.939	2.403	2.711	-	2.148	-
**4_m2**	2.585	1.908	2.471	2.732	2.271	2.489	2.483
**4_m3**	2.312	-	2.452	2.518	2.364	2.333	2.298
**4_m4**	2.600	-	2.616	2.722	2.386	2.243	2.249
**4_m5**	3.272	-	2.454	2.723	2.240	2.422	2.466
**4_m6**	3.028	-	2.259	2.155	2.259	2.155	2.509

Table S6: Reaction (electronic) energies (kcal/mol) for the oxidation of peptides **1** and **4** to the sulfoxide (SO) or sulfone (SO_2) calculated at BP86-D(MARIJ)/cc-pVTZ [in vacuum (vac) or solvent (sol) water] level of theory.

reaction	reaction energy$_{vac}$	reaction energy$_{sol}$
**1_m2** + 0.5 O_2 → **1_m2**_SO	-30.5	-22.9
**1_m2** + O_2 → **1_m2**_SO_2	-77.4	-72.8
**4_m2** + 0.5 O_2 → **4_m2**_SO	-29.8	-30.6
**4_m2** + O_2 → **4_m2**_SO_2	-67.9	-69.5
**4_m6** + 0.5 O_2 → **4_m6**_SO	-33.9	-42.7
**4_m6** + O_2 → **4_m6**_SO_2	-78.6	-78.3

Table S7: Structures (xyz coordinates) of all model peptides are given in Å (BP86-D(MARIJ)/cc-pVTZ).

1_m1:

```
N    -4.1587869    1.5020574   -3.6167253
C    -3.6754609    0.2202572   -3.7613339
C    -4.0837658   -0.1805837   -5.0216781
N    -4.7931723    0.8287391   -5.6497265
C    -4.8147079    1.8244291   -4.7706341
C    -2.7913053   -0.4066681   -2.7346913
C    -1.8036472    0.6145072   -2.1179679
N    -0.6107155   -0.0949528   -1.6123028
Ni   -0.9567505   -0.7351741    0.2428433
S     0.1528594   -2.6675780    0.1226982
C     0.1078791   -3.2282858   -1.6302129
C     1.2738699   -2.7204514   -2.5705731
C     0.6115695   -2.4322457   -3.9322856
N     0.5445130   -1.1161884   -4.2871500
C    -0.2328529   -0.6337666   -5.4052912
C    -0.3673163    0.8870753   -5.2585935
O     0.4636270    1.5375283   -4.6210193
```

S	-1.4409254	-0.9310505	2.3650244
C	-2.7697561	0.3584571	2.4533020
C	-2.4037242	1.4936340	1.5056809
N	-1.9835607	0.8898312	0.2352426
C	-2.4437667	1.3592017	-0.9382030
O	-3.2898738	2.2702987	-1.1209982
C	-1.2299413	2.3244921	2.0342062
O	-0.4284508	2.8888647	1.1126271
N	-0.9823001	2.4438083	3.2864677
C	0.2979381	3.0443930	3.6802008
C	0.4270182	2.9793734	5.2178895
C	-0.7264547	3.5968308	6.0127160
O	-1.9629340	3.3102230	5.5521884
C	1.5455414	2.3427040	3.0777979
O	2.6342500	2.9233620	3.0717179
N	1.3672424	1.0625319	2.6680969
C	2.4297566	0.2741103	2.0879288
C	2.2739752	-1.2034948	2.5052944
C	2.8641582	-1.5732319	3.8737236
C	2.1781507	-0.8391397	5.0338371
C	2.4621090	0.4357900	0.5478246
O	1.5621622	1.0154918	-0.0831712
N	3.5305638	-0.1177001	-0.0951011
C	3.7286877	0.0584104	-1.5474210
C	5.2520939	0.0922039	-1.6922754
C	5.6975420	-0.9178967	-0.6262409
C	4.7570579	-0.6377981	0.5521138
C	3.1144171	-1.0666305	-2.4237288
N	1.9487879	-1.5859877	-1.9631772
O	3.6146076	-1.3812232	-3.5070691
C	2.7652708	-3.0954698	4.0526883
O	-0.5551454	4.2600906	7.0181036
O	0.0470436	-3.3276312	-4.5609397
O	-0.5106681	-3.9965980	3.0498401
O	3.2727742	-3.5558973	0.1132003
H	0.1788170	0.5474158	-1.4417443
H	-1.5150942	1.3515811	-2.8812976
H	-2.2218429	-1.2128547	-3.2204914
H	-3.3574806	-0.8824410	-1.9176838
H	-3.9108176	-1.1404035	-5.5006970
H	-5.2932206	2.7871475	-4.9182841
H	-3.2552000	2.1723490	1.3235558
H	-2.8400062	0.7037688	3.4910872
H	-3.7253102	-0.0827221	2.1452465
H	-1.8435668	2.8174970	4.6712057
H	0.3706971	4.0907861	3.3477376
H	0.4808008	1.9178567	5.5112022
H	1.3569338	3.4626651	5.5314926
H	0.4352952	0.6284625	2.6749168
H	3.3795600	0.6810488	2.4725160
H	1.2036955	-1.4511855	2.4958168
H	2.7251078	-1.8493774	1.7461264
H	3.9327566	-1.2883338	3.8694979

H	1.0939208	-1.0238332	5.0115943
H	2.3345687	0.2454469	4.9794077
H	2.5655585	-1.1903280	6.0013210
H	1.7183585	-3.4281191	3.9807486
H	3.1670623	-3.4095117	5.0270340
H	3.3337082	-3.6187027	3.2682031
H	3.2370799	0.9916212	-1.8579602
H	5.5548136	-0.1782222	-2.7087389
H	5.6227767	1.1003713	-1.4588352
H	5.5262848	-1.9405979	-0.9905788
H	6.7527509	-0.8225565	-0.3434563
H	4.5410267	-1.5482247	1.1205414
H	5.1683693	0.1252563	1.2311331
H	1.6648768	-1.4154432	-0.9882402
H	1.9950564	-3.5336505	-2.7288387
H	-0.8614295	-2.9411607	-2.0519131
H	0.1309980	-4.3219009	-1.6206631
H	1.2028453	-0.4426524	-3.8992534
H	0.2519654	-0.8473115	-6.3739934
H	-1.2107447	-1.1344037	-5.4231267
N	-1.4164420	1.4522322	-5.9155044
H	-0.4733483	2.3217935	0.3100621
H	-0.2874043	-0.7402826	-2.3345594
H	-3.9798844	2.0563923	-2.7490752
H	3.3011523	-4.2705407	0.7690052
H	2.3429372	-3.2230912	0.1927551
H	-0.3163524	-3.9422801	2.0911346
H	-1.0546756	-3.1919656	3.1712874
H	-2.2546764	0.9062298	-6.1106382
H	-1.5629472	2.4428707	-5.7499019

1_m2:

N	-3.0967670	-2.3301942	-3.1858494
C	-3.3551011	-1.2750813	-2.3202613
C	-3.6164269	-1.7676320	-1.0565507
N	-3.5285171	-3.1367117	-1.1505729
C	-3.2049830	-3.4305682	-2.4442432
C	-3.1554365	0.1461281	-2.7358993
C	-2.0623720	0.8699358	-1.9094191
N	-0.9344588	-0.0373924	-1.5381406
Ni	-1.1050457	-0.6633827	0.3910458
S	0.0820778	-2.5684892	0.3412541
C	0.0611364	-3.1682457	-1.3942831
C	1.2856966	-2.7580929	-2.2714322
C	0.7290860	-2.7999902	-3.6962661
N	0.3718007	-1.5880616	-4.2005750
C	-0.5119869	-1.4578237	-5.3282677
C	-0.9380302	0.0014254	-5.4471797
O	-0.4114699	0.9042807	-4.7978902
S	-1.5690805	-1.0082334	2.5005056
C	-2.8659763	0.3044430	2.7026121

C	-2.5156258	1.4735597	1.7964836
N	-2.1201486	0.9357989	0.4890662
C	-2.6563060	1.4807864	-0.6353141
O	-3.5122004	2.3769753	-0.6967462
C	-1.3419999	2.2938479	2.3316734
O	-0.5584866	2.8697760	1.4061372
N	-1.0795182	2.3887452	3.5851739
C	0.1889995	3.0069245	3.9816956
C	0.3332593	2.9079249	5.5171155
C	-0.8331257	3.4647979	6.3379843
O	-2.0625816	3.1603710	5.8747568
C	1.4476001	2.3504506	3.3595643
O	2.5331494	2.9378296	3.4060986
N	1.2883411	1.0948549	2.8746570
C	2.3692512	0.3486513	2.2751072
C	2.3148492	-1.1178445	2.7529167
C	3.0402097	-1.3864102	4.0804719
C	2.5199461	-0.5278926	5.2412685
C	2.3370337	0.4708547	0.7334119
O	1.3718917	0.9556063	0.1199203
N	3.4302840	-0.0021259	0.0698280
C	3.5748798	0.1722233	-1.3911736
C	5.0856938	0.3261182	-1.5715018
C	5.6370886	-0.6383061	-0.5116354
C	4.7034504	-0.4307146	0.6881398
C	3.0360195	-1.0343371	-2.2107386
N	1.7914839	-1.4588836	-1.8490429
O	3.6677807	-1.5259349	-3.1452199
C	2.9356315	-2.8811198	4.4130202
O	-0.6718868	4.0959366	7.3663140
O	0.4707691	-3.8685552	-4.2511066
O	-2.6183655	-3.9799886	1.3599985
O	3.2378500	-3.3032663	0.2651240
H	-0.0465613	0.4765069	-1.5475892
H	-1.6824256	1.7015818	-2.5190897
H	-4.0675000	0.7528292	-2.6331811
H	-2.8790186	0.1484498	-3.7964823
H	-3.7974510	-1.2693762	-0.1130996
H	-3.0346710	-4.4428427	-2.7936935
H	-3.3718804	2.1570058	1.6550619
H	-2.8963407	0.5999283	3.7577324
H	-3.8418068	-0.1034256	2.4095299
H	-1.9338460	2.7024369	4.9729686
H	0.2352064	4.0633040	3.6754092
H	0.4239263	1.8414652	5.7822580
H	1.2501870	3.4116558	5.8363169
H	0.3623823	0.6554227	2.8463932
H	3.3037483	0.8239788	2.6156761
H	1.2559516	-1.4048405	2.8402979
H	2.7364950	-1.7787647	1.9891786
H	4.1079988	-1.1427070	3.9289337
H	1.4356193	-0.6662552	5.3645010
H	2.7042979	0.5410804	5.0755628

H	3.0095697	-0.8117380	6.1845343
H	1.8846923	-3.1644470	4.5747064
H	3.4962442	-3.1292568	5.3259137
H	3.3242537	-3.4990483	3.5903881
H	2.9990554	1.0589484	-1.6921655
H	5.3905193	0.0666859	-2.5901420
H	5.3788637	1.3626694	-1.3524630
H	5.5415280	-1.6705881	-0.8746617
H	6.6871040	-0.4543630	-0.2538092
H	4.5649395	-1.3548757	1.2596602
H	5.0740265	0.3589128	1.3605582
H	1.4269090	-1.1556094	-0.9376859
H	2.0859902	-3.5046747	-2.1873730
H	-0.8632619	-2.8049731	-1.8556236
H	0.0009533	-4.2613367	-1.3944442
H	0.7261164	-0.7294707	-3.7847975
H	-0.0371926	-1.7748250	-6.2728543
H	-1.3998458	-2.0923288	-5.1643224
N	-1.9331737	0.2321261	-6.3594481
H	-0.6752031	2.3263235	0.5879132
H	-0.8551897	-0.7876672	-2.2305985
H	3.2233963	-4.1968954	0.6412874
H	2.2988192	-3.0006562	0.3715019
H	-1.6995100	-3.8122725	1.0271307
H	-2.7258106	-3.1527924	1.8829929
H	-2.5321343	-0.5349939	-6.6433554
H	-2.3427019	1.1602398	-6.3549257
H	-3.4130246	-3.7193936	-0.3004914

1_m3:

N	-4.3335813	1.3207734	-3.4226917
C	-3.6779471	0.1416735	-3.6947949
C	-4.0969762	-0.2251825	-4.9658904
N	-4.9920832	0.7032539	-5.4726589
C	-5.1016245	1.6156079	-4.5106834
C	-2.6665840	-0.4267576	-2.7515762
C	-1.8717833	0.6787017	-2.0197322
N	-0.6235057	0.1245203	-1.4587673
Ni	-1.0315959	-0.7248225	0.3029677
S	0.2154480	-2.5817418	0.0827816
C	0.0804780	-3.0650937	-1.6868997
C	1.2714067	-2.6164347	-2.6210513
C	0.6318035	-2.2660523	-3.9725185
N	0.6043807	-0.9381174	-4.2849916
C	-0.1540636	-0.4228689	-5.4039621
C	-0.2966214	1.0926253	-5.2272015
O	0.6378476	1.7652017	-4.7922171
S	-1.4526154	-0.9938041	2.4283792
C	-2.7714094	0.2989184	2.6344227
C	-2.5650393	1.3860585	1.5849664
N	-2.2854418	0.7153135	0.3179157
C	-2.6581589	1.2658690	-0.8357466

O	-3.5600719	2.1366200	-1.0253551
C	-1.3388280	2.3332078	1.8283412
O	-0.7902959	2.8941743	0.8717721
N	-0.9005435	2.3919124	3.1023947
C	0.3176219	3.1013855	3.4546271
C	0.4515716	3.0926167	5.0002895
C	-0.8550287	3.3979343	5.8123082
O	-1.9009827	2.7466686	5.4405213
C	1.5789995	2.4412302	2.8619838
O	2.6699456	3.0250478	2.8352732
N	1.4172354	1.1434659	2.4879207
C	2.4914816	0.3139523	2.0099732
C	2.2697448	-1.1443246	2.4672972
C	2.7867473	-1.4934974	3.8693020
C	2.0875274	-0.6855009	4.9696399
C	2.6284292	0.4064955	0.4714682
O	1.8126734	0.9943021	-0.2442564
N	3.7079682	-0.2332014	-0.0890480
C	3.9743643	-0.1168272	-1.5354227
C	5.4976062	-0.2495859	-1.6295570
C	5.8120398	-1.2252047	-0.4868983
C	4.8756020	-0.7683248	0.6364690
C	3.2640047	-1.1790724	-2.4158940
N	2.0149666	-1.5283252	-2.0117880
O	3.7765459	-1.6025157	-3.4576394
C	2.6088158	-3.0032573	4.0924423
O	-0.7585062	4.1892150	6.7712147
O	0.0554214	-3.1235587	-4.6467384
O	-0.5999952	-4.1088310	2.8677008
O	3.2741852	-3.6180260	0.1578714
H	0.0009904	0.8727636	-1.1178786
H	-1.6508946	1.4961647	-2.7236764
H	-1.9782561	-1.0636416	-3.3259638
H	-3.1227632	-1.0774903	-1.9874030
H	-3.8162419	-1.1137722	-5.5252187
H	-5.7236138	2.5043179	-4.5565875
H	-3.4621298	2.0195866	1.4815079
H	-2.7168458	0.6813512	3.6610823
H	-3.7499625	-0.1759276	2.4819270
H	-1.5207240	2.2935705	3.9732633
H	0.3065658	4.1344530	3.0731216
H	0.7623052	2.0801176	5.3067188
H	1.2417085	3.7903871	5.2942427
H	0.4774659	0.7357129	2.5003722
H	3.4281852	0.7160817	2.4352432
H	1.1897112	-1.3437921	2.4274410
H	2.7187761	-1.8334964	1.7452632
H	3.8683623	-1.2599614	3.9025310
H	0.9972087	-0.8131702	4.9034657
H	2.2957502	0.3875252	4.8788342
H	2.4150378	-1.0143298	5.9677809
H	1.5566404	-3.2993494	3.9587036
H	2.9299517	-3.2994082	5.1028451

H	3.2053106	-3.5796026	3.3668199
H	3.5980255	0.8578044	-1.8813364
H	5.7971730	-0.6145883	-2.6175300
H	5.9648032	0.7290178	-1.4467865
H	5.5377110	-2.2473580	-0.7842359
H	6.8657940	-1.2191214	-0.1791364
H	4.5870249	-1.6024790	1.2848887
H	5.3317736	0.0239691	1.2534110
H	1.7071251	-1.3026916	-1.0545045
H	1.9456964	-3.4660566	-2.7950630
H	-0.8621987	-2.6601916	-2.0688887
H	0.0021459	-4.1550859	-1.7437729
H	1.2588320	-0.2936659	-3.8454096
H	0.3566581	-0.6040641	-6.3666550
H	-1.1223169	-0.9384823	-5.4548449
N	-1.4850878	1.6218407	-5.6354482
H	-0.1065087	-0.3832247	-2.1779354
H	-4.1583517	1.8414447	-2.5017200
H	3.1864682	-4.2043557	0.9264501
H	2.3791960	-3.1884922	0.1296808
H	-0.3331489	-3.9614477	1.9347546
H	-1.1107342	-3.2859954	3.0306285
H	-2.3239725	1.0366888	-5.6469830
H	-1.6275031	2.6029059	-5.4156262

1_m4:

N	-3.0798066	-1.9664450	-3.1301207
C	-3.3334381	-0.9095581	-2.2627826
C	-3.7050167	-1.4157094	-1.0326085
N	-3.6836321	-2.7867017	-1.1504081
C	-3.2931016	-3.0754906	-2.4236323
C	-3.0444187	0.5128406	-2.6324636
C	-2.0980854	1.2201425	-1.6377163
N	-0.9359622	0.3701356	-1.2574668
Ni	-1.2492988	-0.5008438	0.5479374
S	-0.0419665	-2.3973217	0.2960251
C	-0.1460055	-2.8438743	-1.4882052
C	1.1573053	-2.6062588	-2.2831645
C	0.8226450	-2.7762267	-3.7664910
N	0.5380085	-1.6191225	-4.4456222
C	0.4040338	-1.6057820	-5.8891196
C	-0.9163170	-1.1020069	-6.4849795
O	-0.9268423	-0.6963381	-7.6537256
S	-1.7167660	-1.0031478	2.6203085
C	-2.9606321	0.3373067	2.9735860
C	-2.6925826	1.5272389	2.0594565
N	-2.3911587	0.9942060	0.7300005
C	-2.8254649	1.6605661	-0.3589426
O	-3.7152816	2.5290496	-0.4221729
C	-1.4885342	2.4383330	2.4497591
O	-0.9839657	3.1945396	1.6126750
N	-0.9988360	2.2587280	3.6998583

C	0.2050553	2.9426546	4.1365579
C	0.3767599	2.7104416	5.6603059
C	-0.9192751	2.8556220	6.5320849
O	-1.9493210	2.2209554	6.0969813
C	1.4651374	2.4096561	3.4269931
O	2.5662336	2.9673172	3.5368497
N	1.2885636	1.2290015	2.7755235
C	2.3604824	0.4604319	2.2060031
C	2.2555918	-1.0110656	2.6730730
C	3.0342772	-1.3315917	3.9568181
C	2.5999276	-0.4696652	5.1477674
C	2.3897587	0.5797572	0.6677777
O	1.5096558	1.1577512	0.0193299
N	3.4537303	-0.0024002	0.0263754
C	3.6299899	0.1675661	-1.4291100
C	5.1508361	0.1970365	-1.5943457
C	5.6125828	-0.8018550	-0.5229974
C	4.6796969	-0.5212657	0.6633366
C	2.9994765	-0.9865612	-2.2555237
N	1.7107759	-1.3008586	-1.9243131
O	3.6074732	-1.5469022	-3.1694825
C	2.8872355	-2.8267501	4.2720866
O	-0.8243747	3.5150375	7.5869824
O	0.7350299	-3.8978872	-4.2665200
O	-2.7960760	-3.7671844	1.2845263
O	3.0410437	-3.3154943	0.2338446
H	-0.1160707	0.9463911	-1.0192616
H	-1.7476810	2.1503401	-2.1101553
H	-3.9564372	1.1294502	-2.6598901
H	-2.6073586	0.5226751	-3.6417488
H	-3.9153939	-0.9227278	-0.0928904
H	-3.1412854	-4.0868689	-2.7834812
H	-3.5799741	2.1788413	1.9884239
H	-2.9047484	0.5859448	4.0406831
H	-3.9605485	-0.0648830	2.7589876
H	-1.5901989	1.9992132	4.5522045
H	0.1581379	4.0202077	3.9127602
H	0.7208897	1.6742577	5.8090687
H	1.1565798	3.3792327	6.0372763
H	0.3463269	0.8335895	2.7347389
H	3.2976090	0.9153110	2.5746422
H	1.1873805	-1.2352217	2.8185597
H	2.5936183	-1.6892379	1.8839976
H	4.1036150	-1.1278366	3.7574688
H	1.5210141	-0.5807427	5.3275031
H	2.7972634	0.5962957	4.9767464
H	3.1323226	-0.7688920	6.0636607
H	1.8332506	-3.0708962	4.4728369
H	3.4746715	-3.1105573	5.1584401
H	3.2172068	-3.4451468	3.4237198
H	3.1309391	1.1008909	-1.7282094
H	5.4459830	-0.0917076	-2.6083823
H	5.5230547	1.2084302	-1.3757774

H	5.4424906	-1.8252839	-0.8847108
H	6.6709498	-0.6978575	-0.2515374
H	4.4671603	-1.4327259	1.2328034
H	5.0977355	0.2379758	1.3438127
H	1.3633171	-0.9592235	-1.0188118
H	1.8937130	-3.3865044	-2.0442431
H	-0.9614235	-2.2647228	-1.9318742
H	-0.4111298	-3.9019011	-1.5915853
H	0.8001512	-0.7585647	-3.9734930
H	1.1989507	-1.0053670	-6.3523905
H	0.5323955	-2.6462959	-6.2242030
N	-2.0168963	-1.1569293	-5.7004344
H	-0.6735485	-0.2507606	-2.0265227
H	2.9474464	-4.1872301	0.6481772
H	2.1250165	-2.9346249	0.3294162
H	-1.8754826	-3.6473307	0.9366302
H	-2.8243422	-2.9403681	1.8358275
H	-2.1090970	-1.5707879	-4.7543661
H	-2.8854852	-0.8685706	-6.1378283
H	-3.5911094	-3.3822287	-0.2998481

4_m1:

C	4.9560343	-0.3756145	0.9115363
N	3.7005067	-0.0180931	0.2185288
C	3.8633542	0.0010371	-1.2460747
C	5.3705065	0.1826198	-1.4199623
C	5.9584602	-0.5985305	-0.2325965
C	2.5345686	0.3394072	0.8019052
O	1.5637650	0.7435157	0.1309743
C	3.3574735	-1.3242283	-1.8731560
O	4.1144371	-2.1134670	-2.4553952
C	2.4282272	0.1046107	2.3170519
C	2.1357090	-1.3967552	2.5263782
C	2.3920095	-1.9306699	3.9452639
C	2.2448013	-3.4591715	3.9462734
N	1.4023848	0.9464003	2.8947051
C	1.6926441	2.1877268	3.3593463
O	2.8378717	2.6430134	3.4419074
C	0.5007427	3.0117685	3.9112424
C	0.5158115	2.8740485	5.4497410
C	-0.6065118	3.5979177	6.1983797
O	-0.4243708	4.1957341	7.2423359
N	-0.8131682	2.5976072	3.4105522
C	-1.0020809	2.5852976	2.1430710
O	-0.0862080	3.0558709	1.2797588
C	-2.2531148	1.9872880	1.5052103
N	-1.9159098	1.5594971	0.1378033
C	-2.6078554	2.0564465	-0.8994227
O	-3.4582455	2.9788262	-0.8706980
C	-2.7173893	0.7498779	2.2618755
S	-1.4800969	-0.5868642	1.9563739
Ni	-1.0685378	-0.1311411	-0.1406093

S	-0.7089516	-2.2893700	-0.3424786
C	-0.1309260	-2.7252501	-2.0269629
C	1.3954638	-2.7255771	-2.2997439
N	2.0278067	-1.5403553	-1.7489635
N	-0.9703153	0.6101607	-2.1107536
C	-2.2912994	1.3244282	-2.2174538
C	-3.3876680	0.2749180	-2.5286419
C	-4.6061867	0.8507515	-3.1743137
N	-5.1957309	2.0318416	-2.7718217
C	-6.2717890	2.2479310	-3.5840907
N	-6.4239985	1.2789296	-4.4773233
C	-5.3835969	0.4064118	-4.2283088
C	1.4681221	-2.8071993	-3.8334245
O	1.3775305	-3.8816876	-4.4254887
N	1.4612139	-1.5859502	-4.4586612
C	0.9473867	-1.4688178	-5.7919331
C	-0.5215532	-1.0046991	-5.8008219
N	-1.0741602	-0.8758640	-7.0392867
O	-1.1552078	-0.7410342	-4.7775197
O	-1.8342158	3.4824379	5.6494510
C	1.4784186	-1.3054978	5.0089837
O	4.1821113	-3.4201628	0.2917810
O	1.5456999	-4.2178565	0.6469261
C	0.1990923	1.3846966	-2.4077302
H	-2.2550166	2.0565731	-3.0376242
H	-2.9720999	-0.4671026	-3.2224889
H	-3.6144315	-0.2609990	-1.5911634
H	-5.2463051	-0.5007000	-4.8094944
H	-6.9058331	3.1235152	-3.4856796
H	-3.0340587	2.7654589	1.4601424
H	-2.7645102	0.9337789	3.3423176
H	-3.7029822	0.4378210	1.8948221
H	-1.7154998	3.0160882	4.7533421
H	0.7235542	4.0536873	3.6346734
H	0.4190490	1.8037402	5.6967414
H	1.4738243	3.2259172	5.8434739
H	0.4297588	0.6487074	2.7297666
H	3.3774354	0.3787148	2.7979902
H	1.0935671	-1.5897283	2.2278883
H	2.7567924	-1.9653419	1.8198382
H	3.4393137	-1.6934627	4.2044870
H	0.4227035	-1.4117938	4.7174079
H	1.6778391	-0.2359318	5.1380177
H	1.6188092	-1.7995626	5.9817903
H	1.2069441	-3.7391038	3.7017880
H	2.4708420	-3.8816379	4.9359175
H	2.9192733	-3.9250352	3.2136571
H	3.2621657	0.8200876	-1.6640264
H	5.7105310	-0.2036920	-2.3862519
H	5.6145382	1.2521903	-1.3496019
H	5.9960131	-1.6654437	-0.4793310
H	6.9687207	-0.2695352	0.0398603
H	4.8237755	-1.2900531	1.5021904

H	5.2547009	0.4548515	1.5685186
H	1.4677718	-0.9030946	-1.1743471
H	1.8577980	-3.6330797	-1.8908787
H	-0.6277227	-2.0753458	-2.7585312
H	-0.4904684	-3.7424202	-2.2237147
H	1.3809860	-0.7519664	-3.8657428
H	1.5408795	-0.7613159	-6.3931322
H	1.0146145	-2.4600701	-6.2671500
H	-0.3116306	2.6104936	0.4250325
H	-0.9532278	-0.1735466	-2.7802119
H	-4.7744916	2.6299437	-2.0384092
H	3.2430929	-3.7165227	0.3884665
H	4.2880409	-3.3009675	-0.6694263
H	0.8719604	-3.5019205	0.4613461
H	1.4052766	-4.4283880	1.5833932
H	-0.5944054	-1.2001124	-7.8692134
H	-2.0551585	-0.6290203	-7.1060496
O	1.1043665	0.8922118	-3.0712299
C	0.3217046	2.7955091	-1.9036956
H	-0.6159578	3.2663034	-1.5962796
H	1.0171990	2.7532397	-1.0527367
H	0.7992932	3.3924124	-2.6883800

4_m2:

C	4.8525525	-0.6065785	0.8000585
N	3.6400718	-0.1020483	0.1247520
C	3.8789666	0.1140639	-1.3203874
C	5.3974557	0.2674210	-1.3906664
C	5.8795129	-0.7405703	-0.3357408
C	2.5077462	0.3397842	0.7346051
O	1.5691287	0.8311050	0.0850184
C	3.3931078	-1.0853362	-2.1822546
O	4.1498739	-1.7465024	-2.8940675
C	2.4475281	0.1837597	2.2694188
C	2.2741547	-1.2933671	2.6809293
C	2.8465784	-1.6547373	4.0604332
C	2.6152336	-3.1497355	4.3207997
N	1.3768761	0.9864762	2.8202065
C	1.5880935	2.1923483	3.3970452
O	2.7022266	2.7090062	3.5338971
C	0.3455622	2.8899503	4.0115782
C	0.4855481	2.7876885	5.5469418
C	-0.6608908	3.3837842	6.3664117
O	-0.4816403	4.0257827	7.3839848
N	-0.9409191	2.3141577	3.6101580
C	-1.2622876	2.3495946	2.3691183
O	-0.5163589	2.9815122	1.4502502
C	-2.4874571	1.6261978	1.8111107
N	-2.2086340	1.2854353	0.4062237
C	-3.0613615	1.7344207	-0.5530839
O	-4.0474855	2.4684933	-0.3973205
C	-2.7597931	0.3372488	2.5674347

S	-1.4353386	-0.8572493	2.0914113
Ni	-1.1842678	-0.2658323	-0.0036462
S	-0.1684716	-2.2348328	-0.2671564
C	-0.0002619	-2.6527469	-2.0442287
C	1.4322502	-2.4993811	-2.6264225
N	2.0604611	-1.3120221	-2.0853324
N	-1.3357980	0.5659631	-1.9388798
C	-2.6823650	1.2484704	-1.9567267
C	-3.7632023	0.3212971	-2.5609560
C	-3.7589158	-1.0591912	-1.9894652
N	-3.4252102	-2.1603081	-2.7647649
C	-3.3901307	-3.1939383	-1.9288972
N	-3.7105872	-2.8227980	-0.6604664
C	-3.9234270	-1.4642841	-0.6782203
C	1.1836638	-2.4819717	-4.1413694
O	0.9071753	-3.5300801	-4.7409353
N	1.1034531	-1.2410454	-4.6934911
C	0.2757585	-1.0106806	-5.8763800
C	-1.1806570	-0.9256073	-5.3812291
N	-1.7830186	-2.1252840	-5.1730268
O	-1.6996742	0.1639451	-5.1011553
O	-1.9022053	3.0998973	5.9154316
C	2.2742434	-0.8051162	5.2031708
O	-2.3241840	-3.9043642	1.4733942
O	2.8811728	-3.3396926	0.1372355
C	-0.2125391	1.4063501	-2.2634549
H	-2.6304434	2.1344269	-2.6046451
H	-4.7198274	0.8358058	-2.3922076
H	-3.5862523	0.2509385	-3.6417230
H	-4.1445116	-0.9044407	0.2196339
H	-3.1330062	-4.2128175	-2.1963972
H	-3.3510103	2.3130337	1.8436388
H	-2.7149356	0.4838852	3.6532674
H	-3.7454056	-0.0594098	2.2922549
H	-1.7908236	2.6330385	5.0205361
H	0.4264374	3.9448886	3.7059604
H	0.5411945	1.7191993	5.8133136
H	1.4192482	3.2606383	5.8633007
H	0.4278834	0.6144771	2.7054761
H	3.3842907	0.5865749	2.6854253
H	1.1995655	-1.5300728	2.6478403
H	2.7391592	-1.9446395	1.9319567
H	3.9372293	-1.4784809	4.0208561
H	1.1756362	-0.8629218	5.2128671
H	2.5535574	0.2514442	5.1064285
H	2.6433818	-1.1623849	6.1758751
H	1.5376407	-3.3667773	4.3740761
H	3.0698314	-3.4667428	5.2705060
H	3.0390926	-3.7652181	3.5140362
H	3.3163043	1.0045223	-1.6339705
H	5.7740593	0.0388298	-2.3928765
H	5.6778375	1.2943069	-1.1160960
H	5.8329262	-1.7502369	-0.7632788

H	6.9027015	-0.5537084	0.0124341
H	4.6501754	-1.5688749	1.2858534
H	5.1814848	0.1154822	1.5634460
H	1.5270904	-0.7622064	-1.4058807
H	2.0444948	-3.3780397	-2.3850603
H	-0.7058860	-2.0333702	-2.6121858
H	-0.3200304	-3.6885539	-2.1991828
H	1.1468725	-0.4552011	-4.0355397
H	0.5530108	-0.0577008	-6.3374743
H	0.4295145	-1.8352263	-6.5833548
H	-0.8198027	2.5800840	0.5936524
H	-1.3349908	-0.1950758	-2.6289476
H	2.6365056	-4.2045616	0.5016587
H	2.0118612	-2.8682573	0.0883755
H	-1.5729798	-3.7222146	0.8536251
H	-2.2845240	-3.0613383	1.9867287
H	-1.1876875	-2.9511417	-5.2300046
H	-2.5550199	-2.1490799	-4.4775735
H	-3.4567713	-3.3584150	0.1962000
O	0.6716934	0.9959565	-2.9983231
C	-0.1228581	2.7842451	-1.6695911
H	-1.0670424	3.1925456	-1.2979776
H	0.6081556	2.7136068	-0.8510119
H	0.2926816	3.4518883	-2.4318316

4_m3:

C	5.2527882	0.1145133	0.6524191
N	3.9331703	0.2911949	0.0233465
C	4.0309575	0.4504421	-1.4404440
C	5.5080084	0.7782643	-1.6573920
C	6.2032510	-0.0256699	-0.5462214
C	2.7438148	0.4604884	0.6655959
O	1.7110472	0.7333916	0.0359481
C	3.6091018	-0.8527812	-2.1685279
O	4.3872317	-1.4950610	-2.8860184
C	2.7219815	0.1651994	2.1708053
C	2.5183525	-1.3578737	2.3255507
C	2.6166109	-1.8846021	3.7738704
C	3.1018426	-3.3430612	3.7665258
N	1.6961511	0.9300904	2.8289059
C	1.9443643	2.1861052	3.3037608
O	3.0709171	2.6980703	3.3126464
C	0.7339124	2.8724905	3.9569330
C	0.7725530	2.5844416	5.4815106
C	-0.5512496	2.8505970	6.2793333
O	-0.4408281	3.3825814	7.3993446
N	-0.5256223	2.3768142	3.4314652
C	-0.8489325	2.5287081	2.1347034
O	-0.1454178	3.1392886	1.3120719
C	-2.1608121	1.8221680	1.7032750
N	-2.0480086	1.4034947	0.3036781
C	-2.7383891	2.0254769	-0.6412149

O	-3.5253563	3.0074159	-0.5137010
C	-2.4550848	0.5467228	2.4877328
S	-1.2140388	-0.7224262	1.9622267
Ni	-1.0878107	-0.1734328	-0.1467098
S	-0.3527051	-2.2168439	-0.5925135
C	0.2046402	-2.4132825	-2.3314947
C	1.7339222	-2.3859077	-2.5559350
N	2.3187337	-1.2008591	-1.9602190
N	-1.2258371	0.6531738	-2.0468296
C	-2.5445346	1.3697177	-2.0230372
C	-3.6584472	0.3382328	-2.3133050
C	-4.9680677	0.9487789	-2.7062388
N	-5.4543131	2.1084382	-2.1413400
C	-6.6605902	2.3652879	-2.7220800
N	-6.9943689	1.4442497	-3.6200384
C	-5.9355337	0.5582230	-3.6158668
C	1.9651628	-2.4579246	-4.0725435
O	2.1740088	-3.5433036	-4.6446527
N	1.8020258	-1.2865324	-4.7320974
C	2.0879365	-1.1283896	-6.1481141
C	1.0037496	-1.6384178	-7.1220566
N	0.5866317	-2.9039159	-6.8586868
O	0.5979635	-0.9516095	-8.0612309
O	-1.6339644	2.4330294	5.7182793
C	1.2800974	-1.7890270	4.5238568
O	0.3625872	-4.3131402	1.9176680
O	-2.4352596	-3.6496392	1.4820863
C	-0.0459901	1.4307756	-2.3507057
H	-2.5607887	2.1511528	-2.7978668
H	-3.3379177	-0.3171191	-3.1387828
H	-3.7451829	-0.3067208	-1.4218328
H	-5.9205777	-0.3153012	-4.2616815
H	-7.2544532	3.2356489	-2.4602670
H	-2.9838459	2.5501224	1.7972951
H	-2.3697057	0.6772809	3.5733687
H	-3.4625901	0.1854547	2.2440098
H	-1.2172317	2.2296259	4.2550909
H	0.8551927	3.9498471	3.7642170
H	0.9959874	1.5136127	5.6209071
H	1.5897548	3.1548723	5.9340537
H	0.7263919	0.6335188	2.6729709
H	3.6771537	0.4663016	2.6236814
H	1.5502008	-1.6344477	1.8816267
H	3.2834820	-1.8540439	1.7065416
H	3.3638843	-1.2670810	4.3044900
H	0.5255544	-2.3988918	4.0071852
H	0.9090888	-0.7596252	4.5758004
H	1.3857653	-2.1666645	5.5524294
H	2.3784650	-3.9735390	3.2289638
H	3.1952203	-3.7299438	4.7930930
H	4.0841265	-3.4396989	3.2773451
H	3.3432312	1.2481972	-1.7565943
H	5.8367948	0.4835231	-2.6595189

H	5.6686025	1.8570053	-1.5191556
H	6.2666431	-1.0768029	-0.8587341
H	7.2151258	0.3304405	-0.3143215
H	5.2677899	-0.7699523	1.3040870
H	5.4938083	0.9969758	1.2662099
H	1.7517384	-0.6314115	-1.3168434
H	2.1857375	-3.2966838	-2.1371347
H	-0.2500139	-1.6439965	-2.9706888
H	-0.1627574	-3.3832161	-2.6907251
H	1.5342570	-0.4610993	-4.1880413
H	2.2317433	-0.0653774	-6.3629721
H	3.0218040	-1.6675742	-6.3777930
H	-1.2491844	-0.1175109	-2.7214649
H	-4.8804519	2.6637124	-1.4573060
H	-0.6167676	-4.2269976	1.9721438
H	0.5531618	-3.7132147	1.1690614
H	-2.1174884	-3.5368758	0.5613292
H	-2.2526468	-2.7375884	1.8265320
H	1.0586344	-3.4404343	-6.1165601
H	-0.1075920	-3.3174839	-7.4692392
O	0.7762650	0.9550765	-3.1319318
C	0.0948274	2.7571943	-1.6943794
H	0.0993315	2.6545413	-0.5909020
H	1.0220707	3.2210293	-2.0406951
H	-0.7618026	3.4078478	-1.9228400

4_m4:

C	5.0858294	-0.1126838	0.7165375
N	3.8215436	0.1724002	0.0178574
C	3.9963693	0.2174396	-1.4483176
C	5.5086867	0.3755226	-1.6107265
C	6.0623547	-0.4314769	-0.4250425
C	2.6379934	0.5258171	0.6002789
O	1.6613793	0.8558968	-0.0843655
C	3.4797504	-1.0882695	-2.1174673
O	4.2318071	-1.8702127	-2.7084099
C	2.5647778	0.3809789	2.1293956
C	2.3593748	-1.1180795	2.4410241
C	2.7848150	-1.5893324	3.8406249
C	2.4120280	-3.0725743	3.9891348
N	1.4991940	1.1727748	2.6863764
C	1.7012417	2.4085985	3.2177817
O	2.8071789	2.9663496	3.2362954
C	0.4764751	3.0229429	3.9208261
C	0.6795839	2.8757166	5.4526692
C	-0.5917579	3.0804302	6.3464570
O	-0.4615768	3.7704086	7.3754628
N	-0.7645995	2.3615272	3.5594521
C	-1.2874436	2.4769822	2.3223315
O	-0.7796177	3.1544122	1.4150019
C	-2.5672588	1.6341428	2.0651798
N	-2.5342385	1.1643476	0.6804670

C	-3.3364425	1.7382521	-0.2269798
O	-4.2457964	2.5723202	-0.0434899
C	-2.6951839	0.3926852	2.9383271
S	-1.4593770	-0.8470892	2.3299072
Ni	-1.4306785	-0.2912970	0.2131659
S	-0.2198292	-2.1455795	-0.1237392
C	0.0348877	-2.5612546	-1.8946407
C	1.5042133	-2.5177888	-2.3790742
N	2.1435142	-1.2723706	-1.9914454
N	-1.7966608	0.4332828	-1.7083765
C	-3.0529850	1.2609281	-1.6514060
C	-4.2446635	0.4563433	-2.2382088
C	-4.1531893	-0.9809316	-1.8504228
N	-3.5822499	-1.9042143	-2.7208546
C	-3.3770287	-2.9971288	-1.9875318
N	-3.7998217	-2.8329093	-0.7042582
C	-4.2676769	-1.5430283	-0.5947777
C	1.4120643	-2.7492625	-3.8967562
O	1.2719029	-3.8840411	-4.3544402
N	1.3502709	-1.6150144	-4.6622443
C	0.7675822	-1.6738430	-5.9903830
C	-0.7772316	-1.8130731	-6.0328522
N	-1.4280247	-1.2745844	-4.9704139
O	-1.3281304	-2.3557807	-6.9916006
O	-1.6478134	2.4535896	5.9563016
C	2.1805670	-0.7588207	4.9790212
O	-2.3967253	-3.8332769	1.4447005
O	2.4050121	-3.8594599	0.5308772
C	-0.6135581	1.0871188	-2.1657419
H	-2.9176110	2.1661960	-2.2581329
H	-5.1516449	0.9509411	-1.8645962
H	-4.2391343	0.5228306	-3.3350803
H	-4.5573628	-1.1125584	0.3548022
H	-2.9189750	-3.9131209	-2.3438372
H	-3.4360964	2.3016068	2.1943912
H	-2.4998642	0.5816621	4.0008522
H	-3.7033919	-0.0297292	2.8284343
H	-1.3403780	2.1793369	4.4544137
H	0.4594170	4.0862938	3.6345935
H	1.0176314	1.8464384	5.6540737
H	1.4731329	3.5565272	5.7748590
H	0.5507162	0.7912507	2.6517414
H	3.5018886	0.7528692	2.5720773
H	1.2939883	-1.3410860	2.2814169
H	2.8994435	-1.7220984	1.6996437
H	3.8862302	-1.5005268	3.9011411
H	1.0849565	-0.7224649	4.8947099
H	2.5440324	0.2758134	4.9686818
H	2.4350894	-1.1983950	5.9557031
H	1.3167290	-3.1819809	3.9938084
H	2.7972971	-3.4874943	4.9331041
H	2.7959969	-3.6677553	3.1486280
H	3.4150422	1.0633224	-1.8424253

H	5.8461790	-0.0133775	-2.5772503
H	5.7805421	1.4376550	-1.5237937
H	6.0152260	-1.4996135	-0.6746584
H	7.0979394	-0.1763700	-0.1654941
H	4.9724186	-0.9509231	1.4170705
H	5.4056225	0.7725404	1.2902065
H	1.6216477	-0.6066879	-1.4081477
H	2.0653421	-3.3613478	-1.9545350
H	-0.5689824	-1.8808111	-2.5010995
H	-0.3392215	-3.5731550	-2.0865480
H	1.3439536	-0.7309695	-4.1566867
H	1.0535006	-0.7748620	-6.5544042
H	1.1567694	-2.5520825	-6.5167384
H	-1.9589938	-0.4093973	-2.2748825
H	1.9044609	-4.6898829	0.5543677
H	1.6820262	-3.1832637	0.4823708
H	-1.6191199	-3.5966781	0.8679287
H	-2.4017837	-3.0061450	1.9907165
H	-0.8957424	-0.8285978	-4.2227977
H	-2.3987463	-1.5087678	-4.7647631
H	-3.4685643	-3.3851433	0.1203668
O	0.0773784	0.5226907	-3.0187552
C	-0.3087073	2.4349398	-1.5994487
H	-0.5559102	2.5074148	-0.5278659
H	0.7526919	2.6403832	-1.7542733
H	-0.9066810	3.1915352	-2.1330720

4_m5:

C	5.2797049	-0.1377706	0.7153442
N	3.9643416	0.0673004	0.0974067
C	4.0513379	0.2203382	-1.3681954
C	5.5335586	0.5178815	-1.5960394
C	6.2181061	-0.3040387	-0.4909017
C	2.7919334	0.3466663	0.7513032
O	1.7833016	0.6921256	0.1376720
C	3.5960368	-1.0667643	-2.1061336
O	4.3661657	-1.7216062	-2.8308232
C	2.7673308	0.0839291	2.2670964
C	2.5177041	-1.4276095	2.4565572
C	2.5979123	-1.9358696	3.9108966
C	2.9572187	-3.4308427	3.9157659
N	1.7549501	0.8996199	2.8927682
C	2.0312371	2.1616050	3.3264502
O	3.1771843	2.6338347	3.3734581
C	0.8229060	2.9344502	3.8886461
C	0.8316614	2.7788852	5.4309150
C	-0.4501189	3.2741295	6.1856464
O	-0.2727659	3.9972423	7.1940074
N	-0.4366646	2.4362025	3.3700936
C	-0.7585849	2.5600119	2.0525643
O	-0.0464733	3.1520626	1.2345757
C	-2.0692155	1.8430326	1.6345945

N	-1.9638830	1.3888656	0.2510540
C	-2.7412294	1.9050311	-0.6893860
O	-3.6036706	2.8350600	-0.5493871
C	-2.3736863	0.5903187	2.4566535
S	-1.1434685	-0.7047934	1.9706574
Ni	-1.0407815	-0.2327808	-0.1831657
S	-0.3520641	-2.2988523	-0.5913842
C	0.1037223	-2.3920969	-2.3650797
C	1.6244728	-2.4233135	-2.6443231
N	2.3028477	-1.3751832	-1.9005442
N	-1.1839382	0.5625051	-2.0928416
C	-2.5089314	1.2045787	-2.0426262
C	-3.5839469	0.1130214	-2.2940418
C	-4.9336967	0.6421767	-2.6736352
N	-5.4324202	1.8195067	-2.1591037
C	-6.6650002	2.0144185	-2.7055297
N	-7.0107468	1.0385985	-3.5433225
C	-5.9249716	0.1800707	-3.5263520
C	1.8178278	-2.2692824	-4.1615029
O	2.0255218	-3.2634047	-4.8953725
N	1.6521812	-1.0121841	-4.6199210
C	1.9996485	-0.5959298	-5.9655484
C	1.0729211	-1.0158364	-7.1254550
N	0.6037206	-2.2844663	-7.0271258
O	0.8386726	-0.2568931	-8.0730791
O	-1.5625586	2.8414898	5.7218375
C	1.2883025	-1.7150962	4.6816684
O	0.3739997	-4.4001193	1.8852694
O	-2.3824478	-3.6060744	1.5537556
C	-0.1362597	1.3713001	-2.4093099
H	-2.6189042	1.9586596	-2.8407701
H	-3.2072895	-0.5356789	-3.0968172
H	-3.6282592	-0.5192134	-1.3898989
H	-5.9067065	-0.7252806	-4.1275154
H	-7.2729492	2.8829932	-2.4669742
H	-2.8911357	2.5769289	1.7107201
H	-2.3032068	0.7505971	3.5395750
H	-3.3836367	0.2357232	2.2091485
H	-1.1490321	2.3601530	4.1421393
H	0.9862539	3.9878422	3.6086329
H	0.9284410	1.7036521	5.6590825
H	1.7162323	3.2885581	5.8282598
H	0.7789412	0.6371979	2.6955626
H	3.7288124	0.3698470	2.7183118
H	1.5435576	-1.6874843	2.0156510
H	3.2680212	-1.9586654	1.8488842
H	3.4043648	-1.3753540	4.4200284
H	0.4671504	-2.2411393	4.1746195
H	1.0206492	-0.6541659	4.7343292
H	1.3715132	-2.1043974	5.7094489
H	2.1851330	-3.9994201	3.3749334
H	3.0179777	-3.8188040	4.9459476
H	3.9279906	-3.6123887	3.4264422

H	3.3710776	1.0278203	-1.6796662
H	5.8471886	0.2149649	-2.6010329
H	5.7192944	1.5931709	-1.4562065
H	6.2456737	-1.3559742	-0.8074500
H	7.2435111	0.0219394	-0.2660129
H	5.2834596	-1.0202748	1.3725222
H	5.5552145	0.7394760	1.3252113
H	1.7219800	-0.7151036	-1.3687734
H	2.0452222	-3.4040735	-2.3816697
H	-0.3485510	-1.5074238	-2.8328249
H	-0.3336315	-3.2974858	-2.8075755
H	1.3543929	-0.2731654	-3.9293322
H	2.0457462	0.4976525	-5.9840759
H	3.0016904	-0.9889059	-6.2170320
H	-4.8406671	2.4115167	-1.4959413
H	-0.5952398	-4.2392418	1.9757668
H	0.5699765	-3.8097946	1.1260257
H	-2.0729281	-3.4915181	0.6258024
H	-2.1781409	-2.6896434	1.8877645
H	1.0167067	-2.9005467	-6.3005038
H	0.0453105	-2.6395493	-7.7943394
O	0.9644072	0.9169251	-2.8381458
C	-0.2073546	2.8769184	-2.1954349
H	-0.1224631	3.0426312	-1.1072364
H	0.6317525	3.3576998	-2.7110117
H	-1.1536240	3.3316449	-2.5157647

4_m6:

C	5.2797049	-0.1377706	0.7153442
N	3.9643416	0.0673004	0.0974067
C	4.0513379	0.2203382	-1.3681954
C	5.5335586	0.5178815	-1.5960394
C	6.2181061	-0.3040387	-0.4909017
C	2.7919334	0.3466663	0.7513032
O	1.7833016	0.6921256	0.1376720
C	3.5960368	-1.0667643	-2.1061336
O	4.3661657	-1.7216062	-2.8308232
C	2.7673308	0.0839291	2.2670964
C	2.5177041	-1.4276095	2.4565572
C	2.5979123	-1.9358696	3.9108966
C	2.9572187	-3.4308427	3.9157659
N	1.7549501	0.8996199	2.8927682
C	2.0312371	2.1616050	3.3264502
O	3.1771843	2.6338347	3.3734581
C	0.8229060	2.9344502	3.8886461
C	0.8316614	2.7788852	5.4309150
C	-0.4501189	3.2741295	6.1856464
O	-0.2727659	3.9972423	7.1940074
N	-0.4366646	2.4362025	3.3700936
C	-0.7585849	2.5600119	2.0525643
O	-0.0464733	3.1520626	1.2345757
C	-2.0692155	1.8430326	1.6345945

N	-1.9638830	1.3888656	0.2510540
C	-2.7412294	1.9050311	-0.6893860
O	-3.6036706	2.8350600	-0.5493871
C	-2.3736863	0.5903187	2.4566535
S	-1.1434685	-0.7047934	1.9706574
Ni	-1.0407815	-0.2327808	-0.1831657
S	-0.3520641	-2.2988523	-0.5913842
C	0.1037223	-2.3920969	-2.3650797
C	1.6244728	-2.4233135	-2.6443231
N	2.3028477	-1.3751832	-1.9005442
N	-1.1839382	0.5625051	-2.0928416
C	-2.5089314	1.2045787	-2.0426262
C	-3.5839469	0.1130214	-2.2940418
C	-4.9336967	0.6421767	-2.6736352
N	-5.4324202	1.8195067	-2.1591037
C	-6.6650002	2.0144185	-2.7055297
N	-7.0107468	1.0385985	-3.5433225
C	-5.9249716	0.1800707	-3.5263520
C	1.8178278	-2.2692824	-4.1615029
O	2.0255218	-3.2634047	-4.8953725
N	1.6521812	-1.0121841	-4.6199210
C	1.9996485	-0.5959298	-5.9655484
C	1.0729211	-1.0158364	-7.1254550
N	0.6037206	-2.2844663	-7.0271258
O	0.8386726	-0.2568931	-8.0730791
O	-1.5625586	2.8414898	5.7218375
C	1.2883025	-1.7150962	4.6816684
O	0.3739997	-4.4001193	1.8852694
O	-2.3824478	-3.6060744	1.5537556
C	-0.1362597	1.3713001	-2.4093099
H	-2.6189042	1.9586596	-2.8407701
H	-3.2072895	-0.5356789	-3.0968172
H	-3.6282592	-0.5192134	-1.3898989
H	-5.9067065	-0.7252806	-4.1275154
H	-7.2729492	2.8829932	-2.4669742
H	-2.8911357	2.5769289	1.7107201
H	-2.3032068	0.7505971	3.5395750
H	-3.3836367	0.2357232	2.2091485
H	-1.1490321	2.3601530	4.1421393
H	0.9862539	3.9878422	3.6086329
H	0.9284410	1.7036521	5.6590825
H	1.7162323	3.2885581	5.8282598
H	0.7789412	0.6371979	2.6955626
H	3.7288124	0.3698470	2.7183118
H	1.5435576	-1.6874843	2.0156510
H	3.2680212	-1.9586654	1.8488842
H	3.4043648	-1.3753540	4.4200284
H	0.4671504	-2.2411393	4.1746195
H	1.0206492	-0.6541659	4.7343292
H	1.3715132	-2.1043974	5.7094489
H	2.1851330	-3.9994201	3.3749334
H	3.0179777	-3.8188040	4.9459476
H	3.9279906	-3.6123887	3.4264422

H	3.3710776	1.0278203	-1.6796662
H	5.8471886	0.2149649	-2.6010329
H	5.7192944	1.5931709	-1.4562065
H	6.2456737	-1.3559742	-0.8074500
H	7.2435111	0.0219394	-0.2660129
H	5.2834596	-1.0202748	1.3725222
H	5.5552145	0.7394760	1.3252113
H	1.7219800	-0.7151036	-1.3687734
H	2.0452222	-3.4040735	-2.3816697
H	-0.3485510	-1.5074238	-2.8328249
H	-0.3336315	-3.2974858	-2.8075755
H	1.3543929	-0.2731654	-3.9293322
H	2.0457462	0.4976525	-5.9840759
H	3.0016904	-0.9889059	-6.2170320
H	-4.8406671	2.4115167	-1.4959413
H	-0.5952398	-4.2392418	1.9757668
H	0.5699765	-3.8097946	1.1260257
H	-2.0729281	-3.4915181	0.6258024
H	-2.1781409	-2.6896434	1.8877645
H	1.0167067	-2.9005467	-6.3005038
H	0.0453105	-2.6395493	-7.7943394
O	0.9644072	0.9169251	-2.8381458
C	-0.2073546	2.8769184	-2.1954349
H	-0.1224631	3.0426312	-1.1072364
H	0.6317525	3.3576998	-2.7110117
H	-1.1536240	3.3316449	-2.5157647
